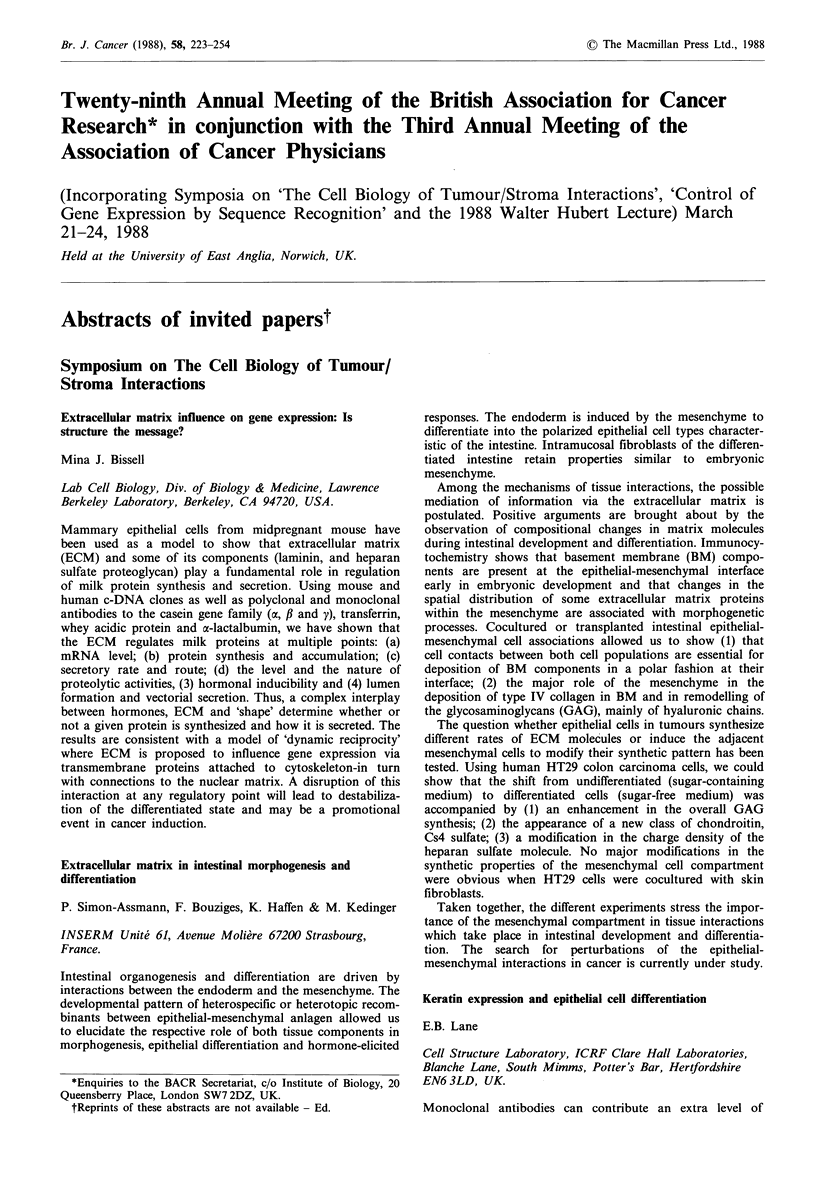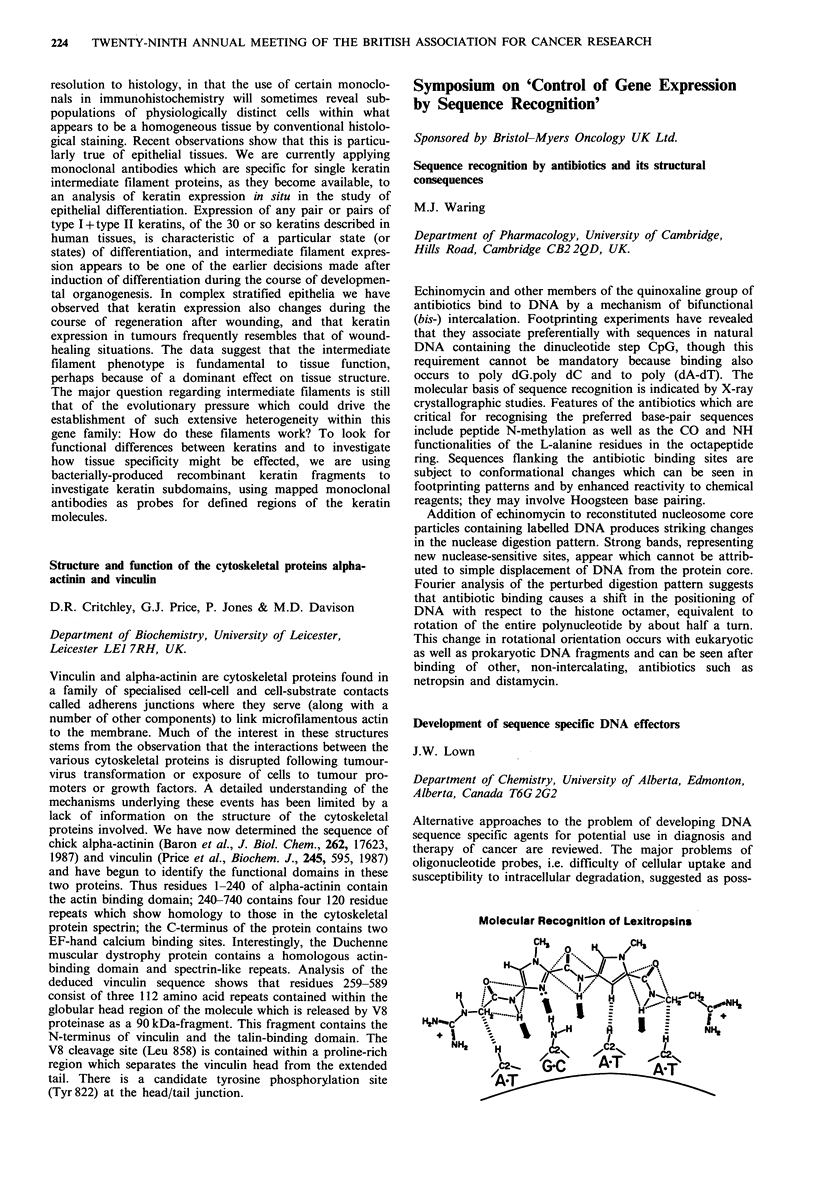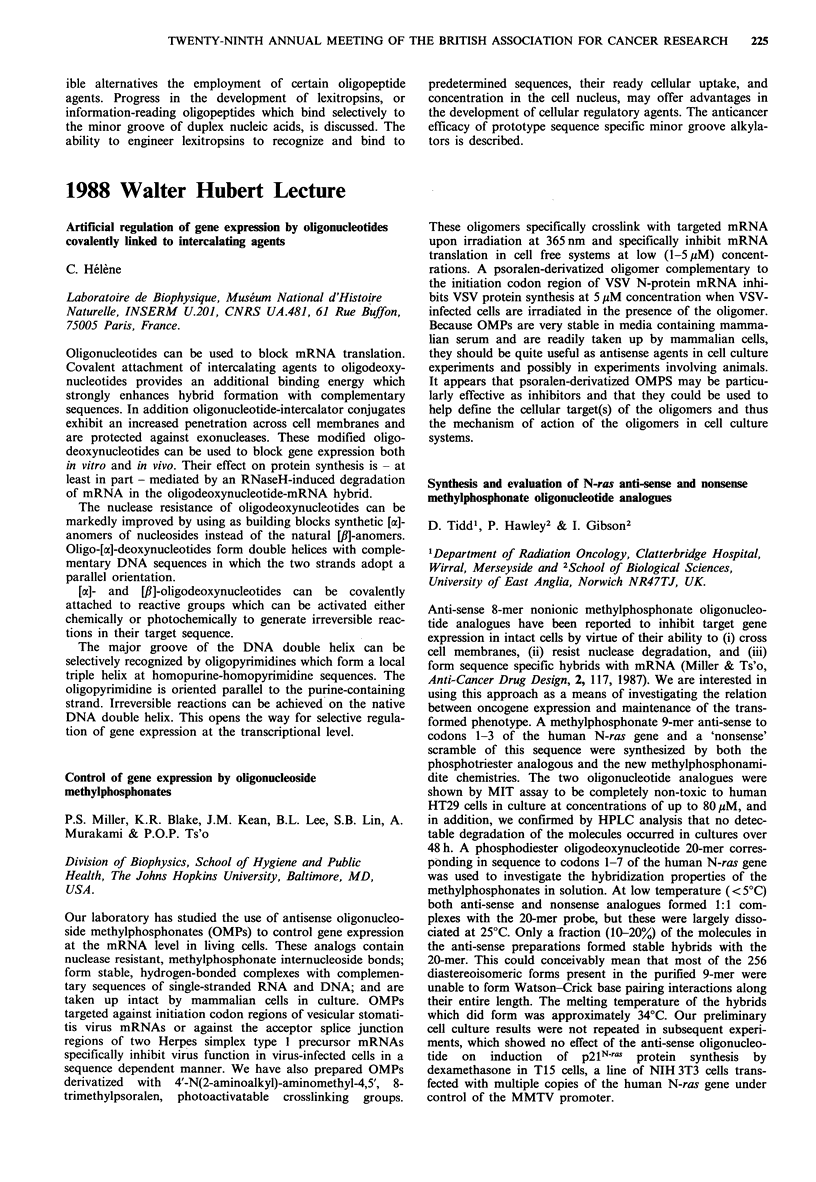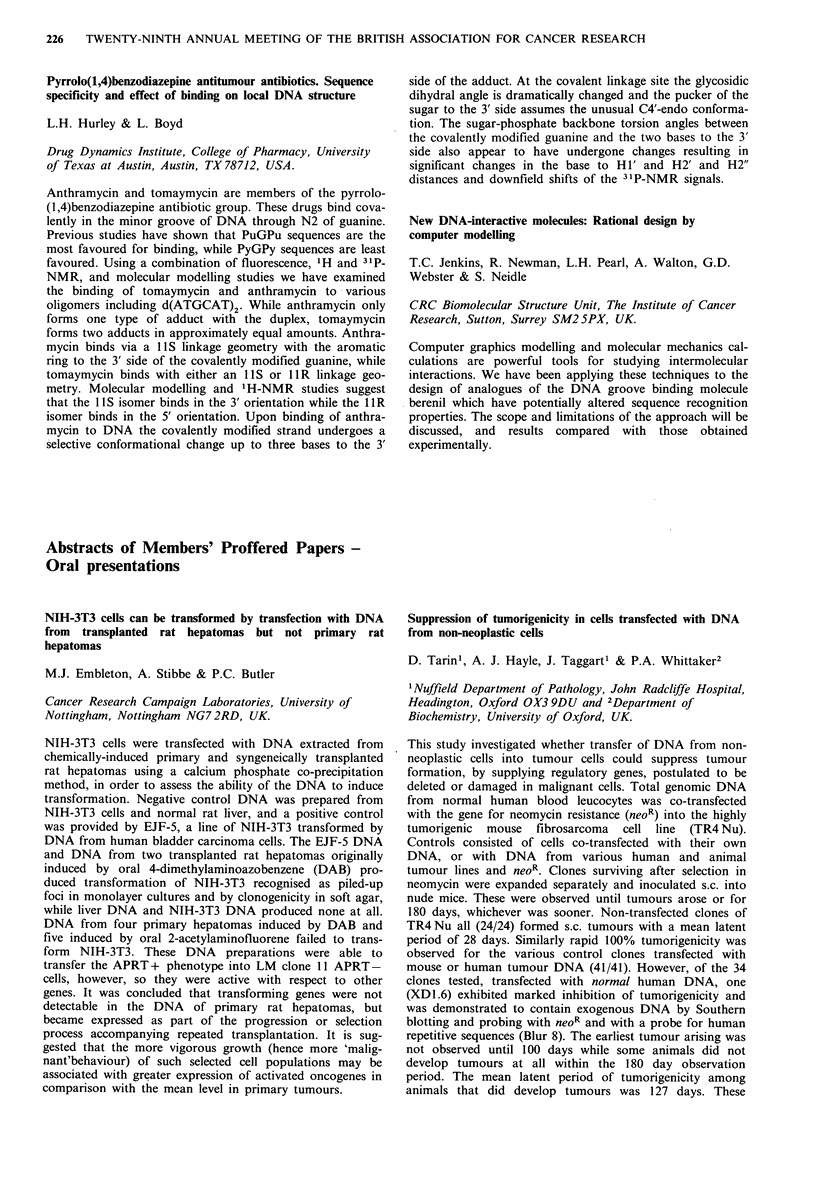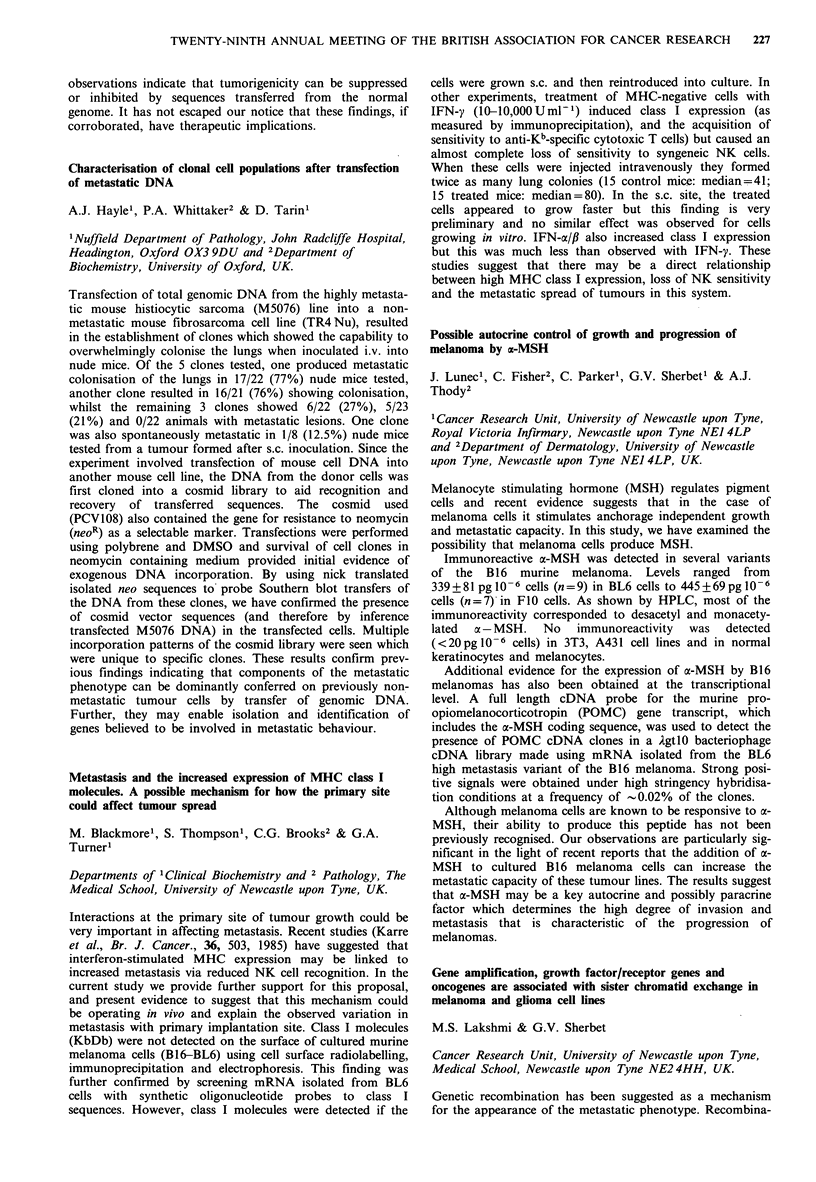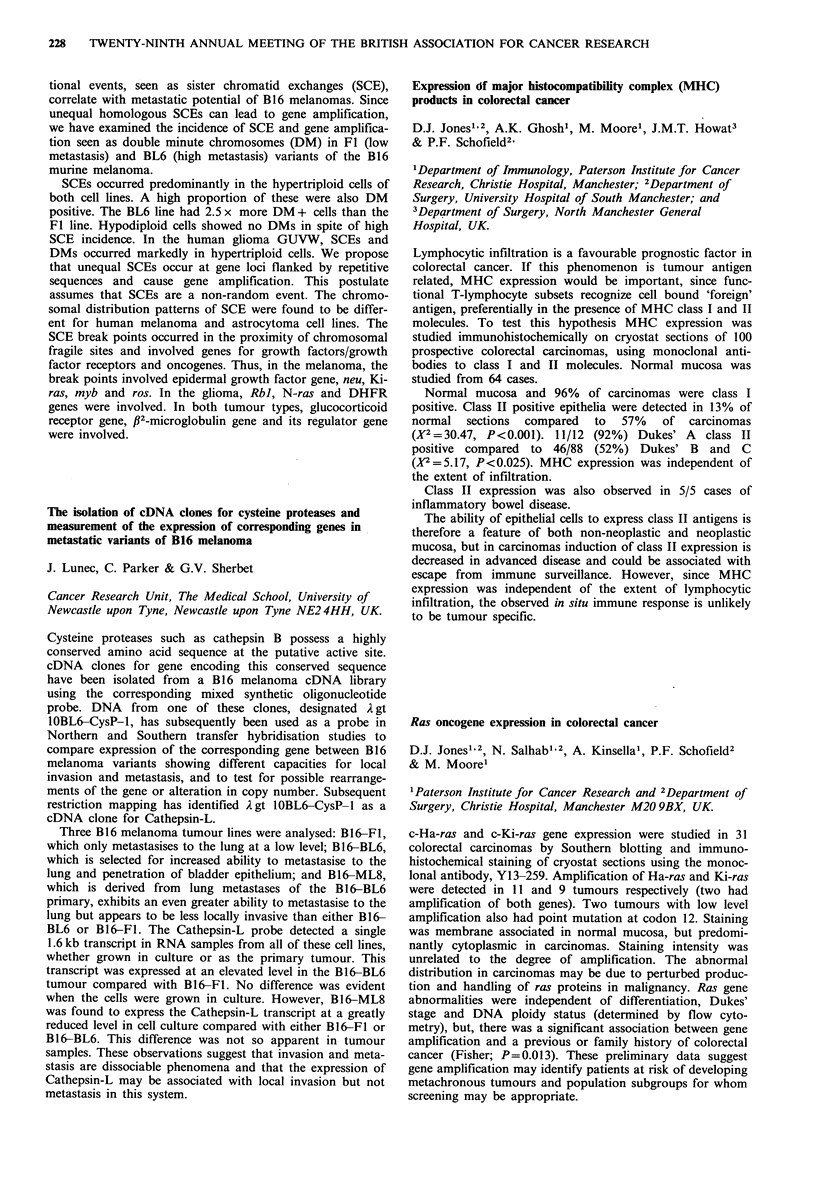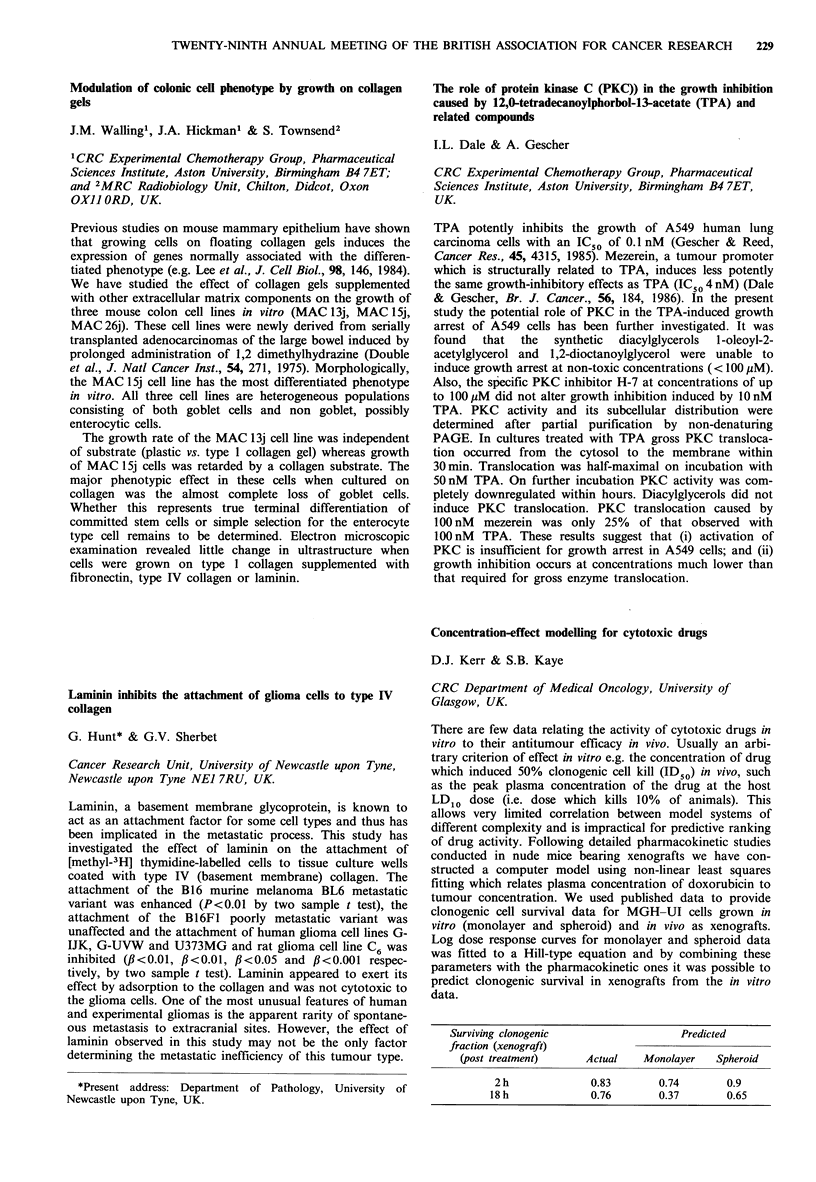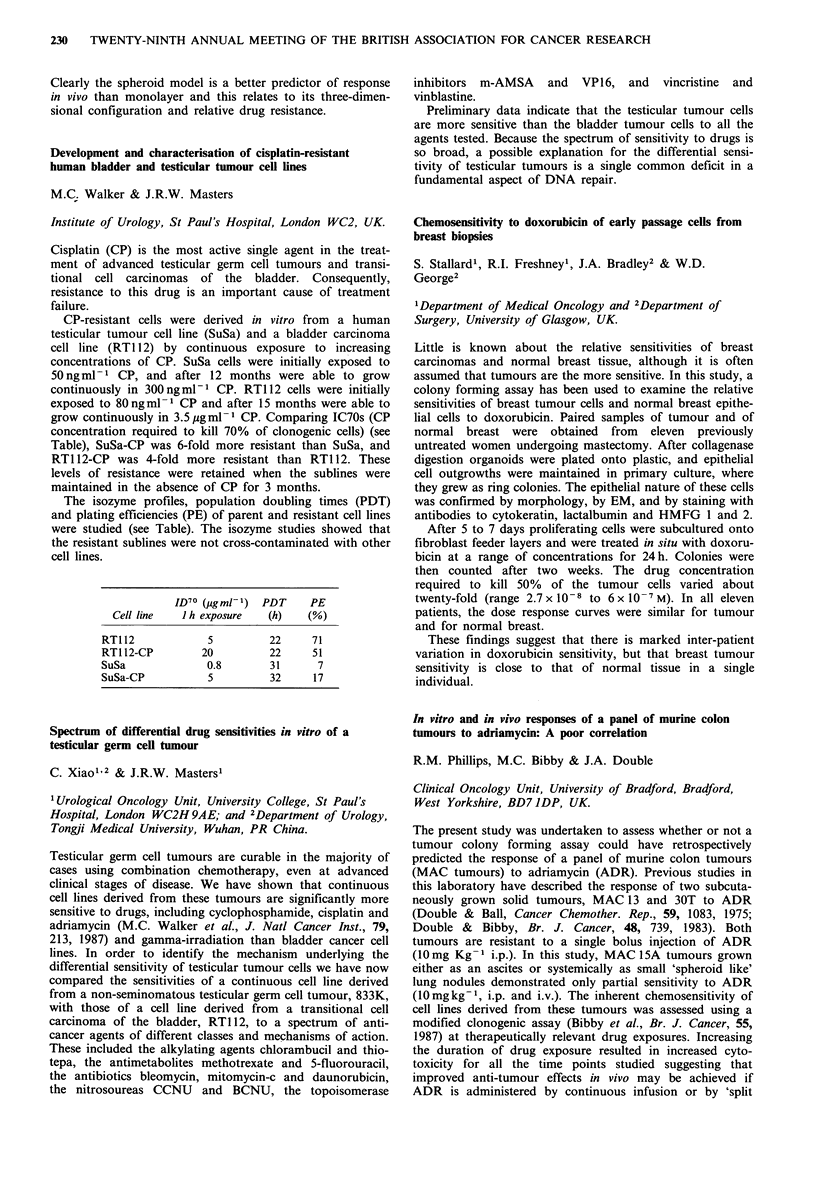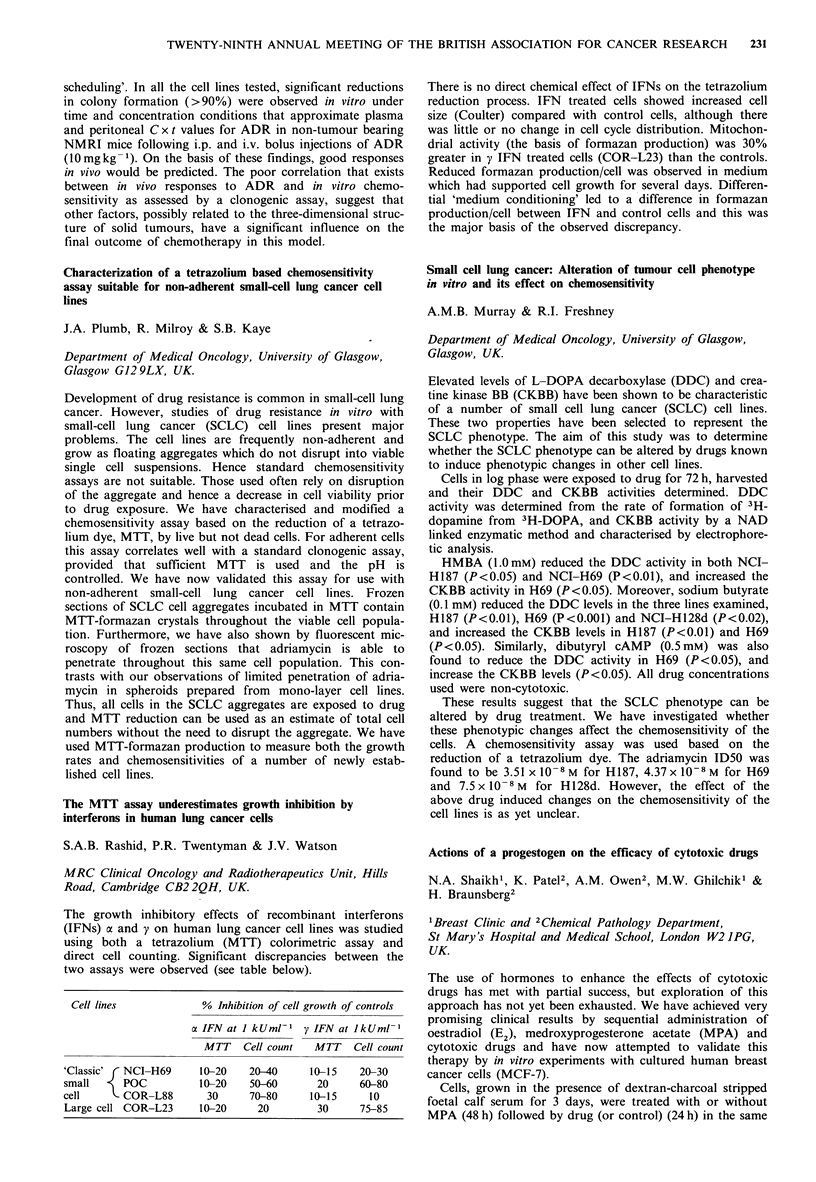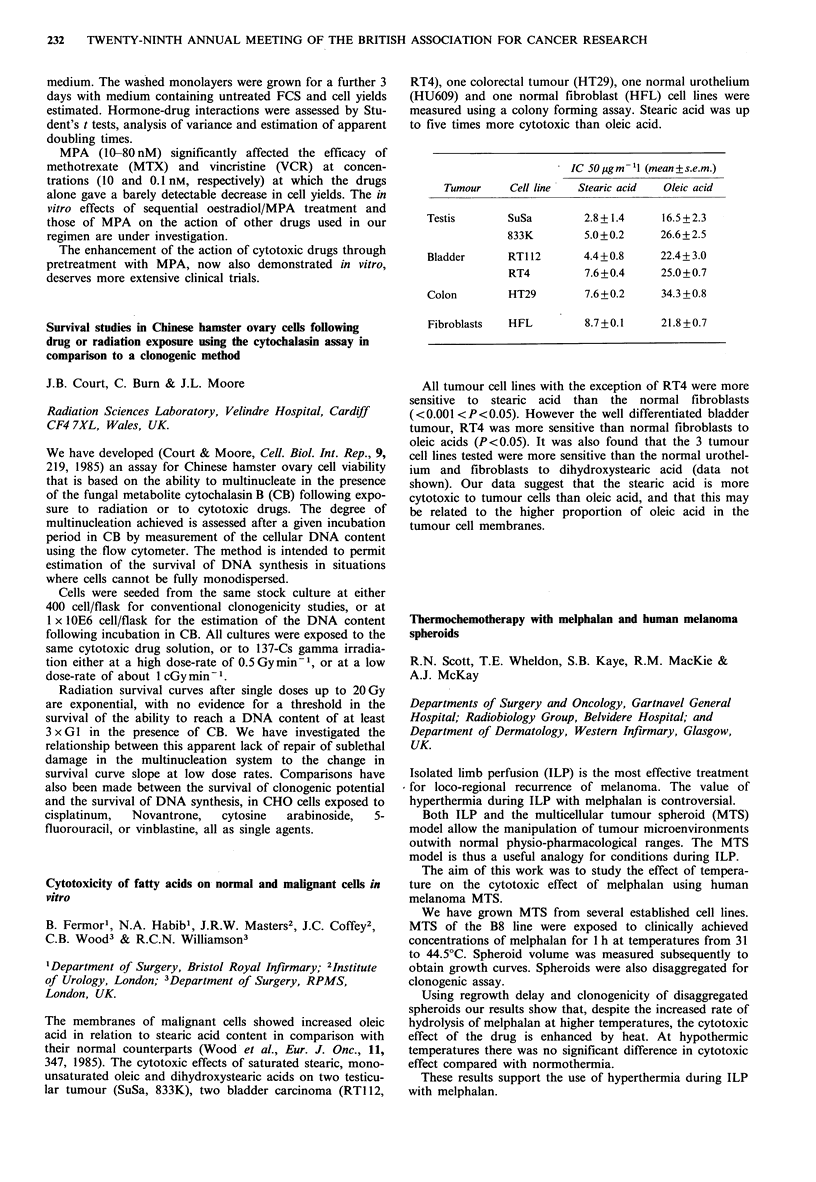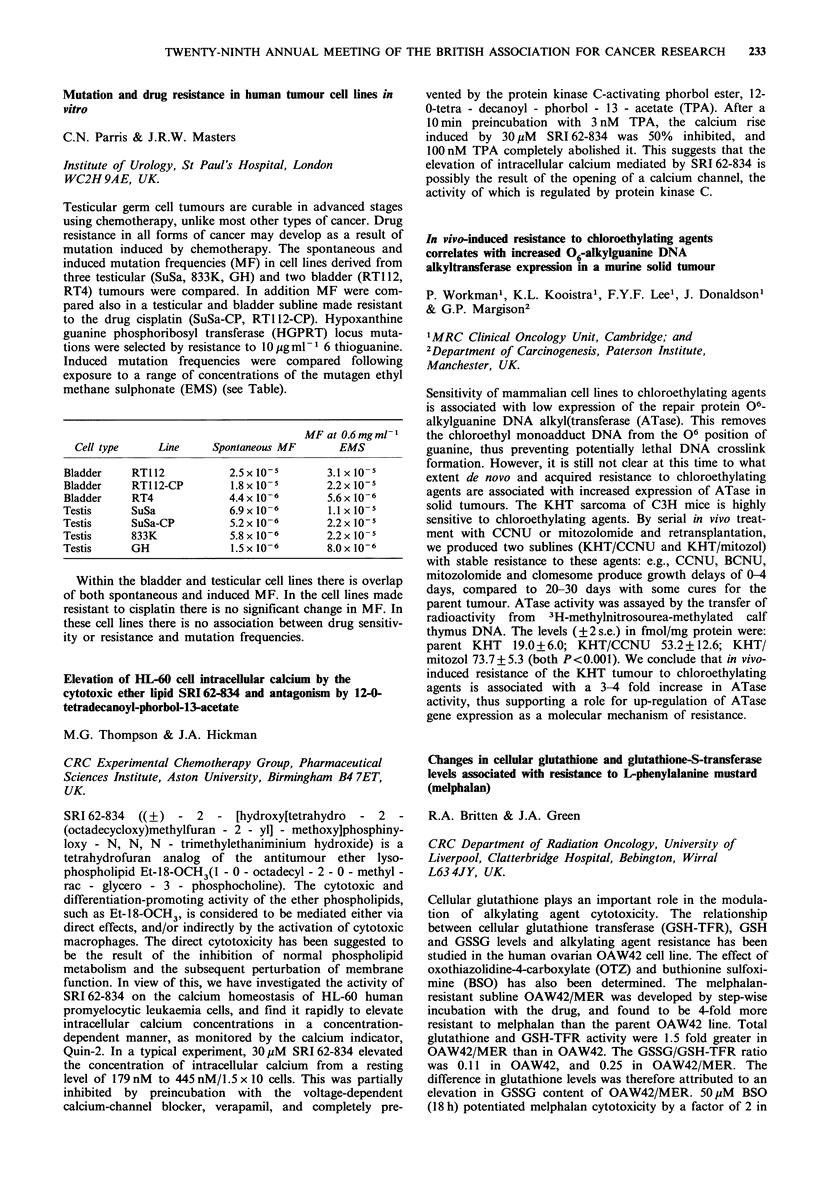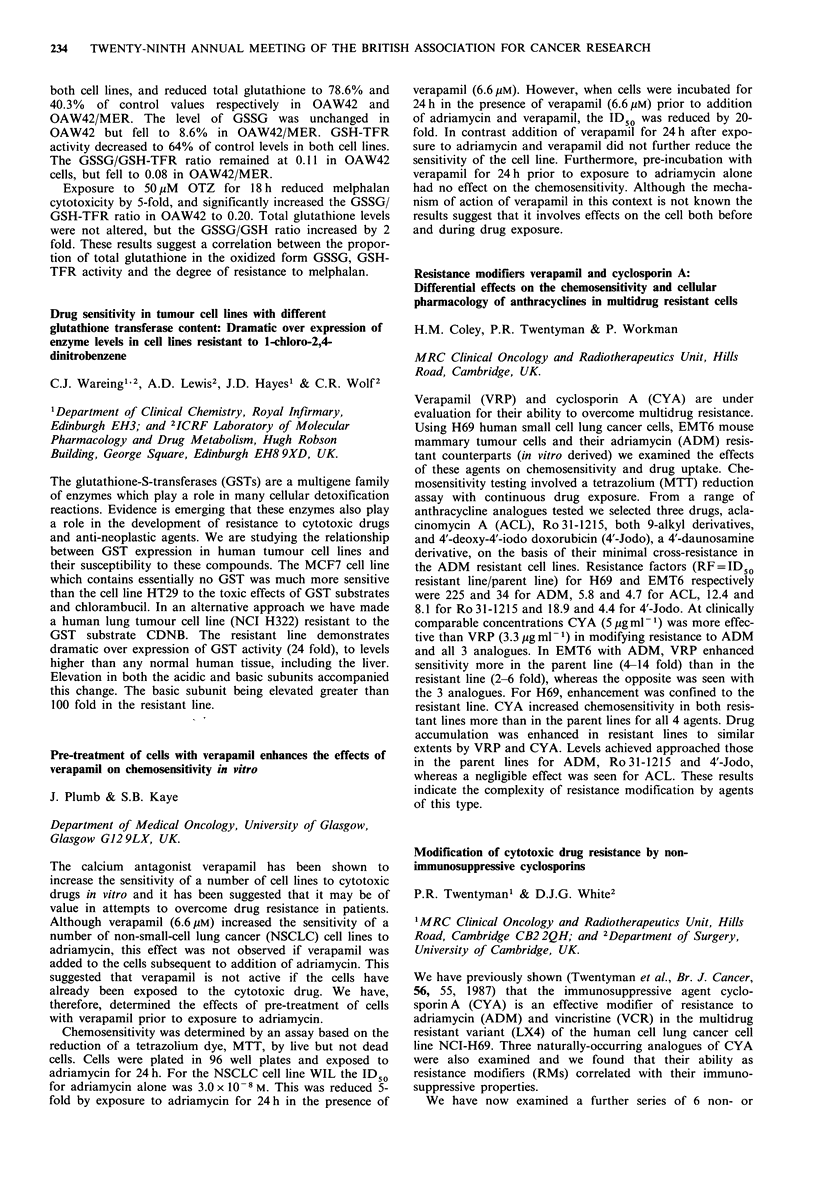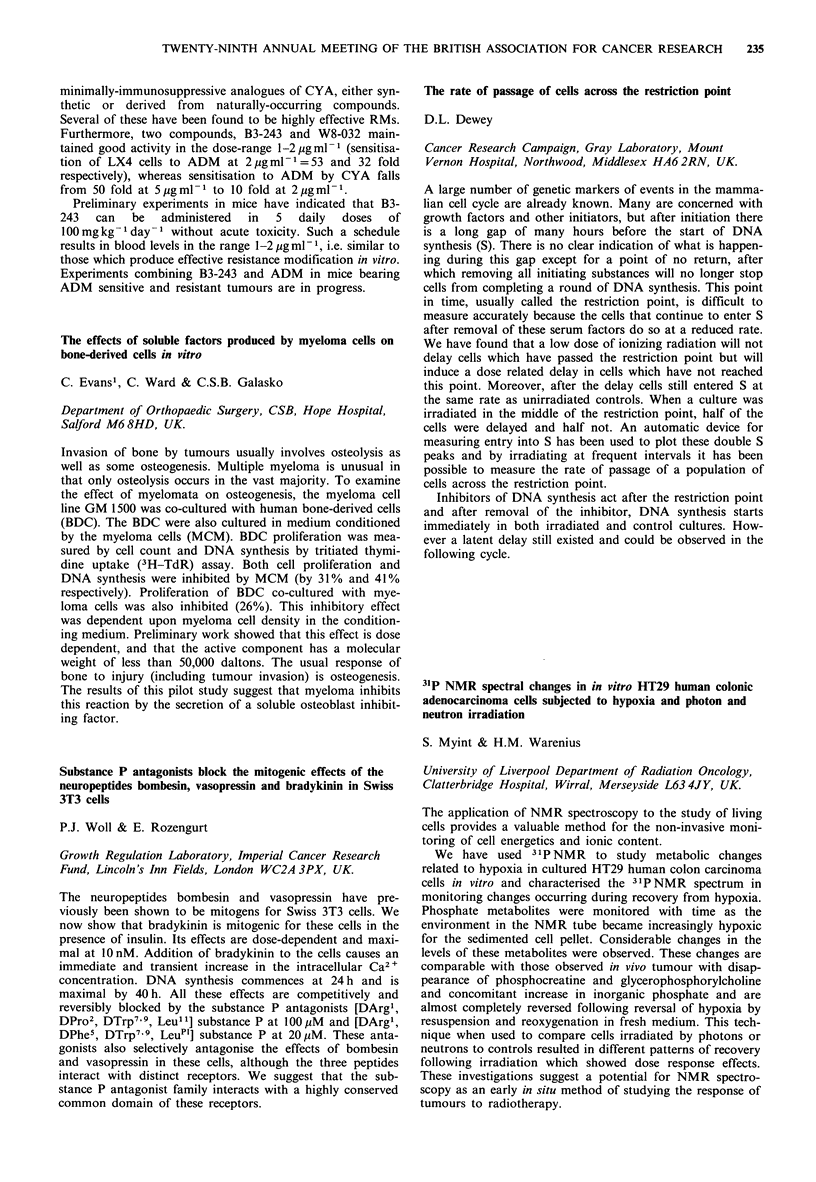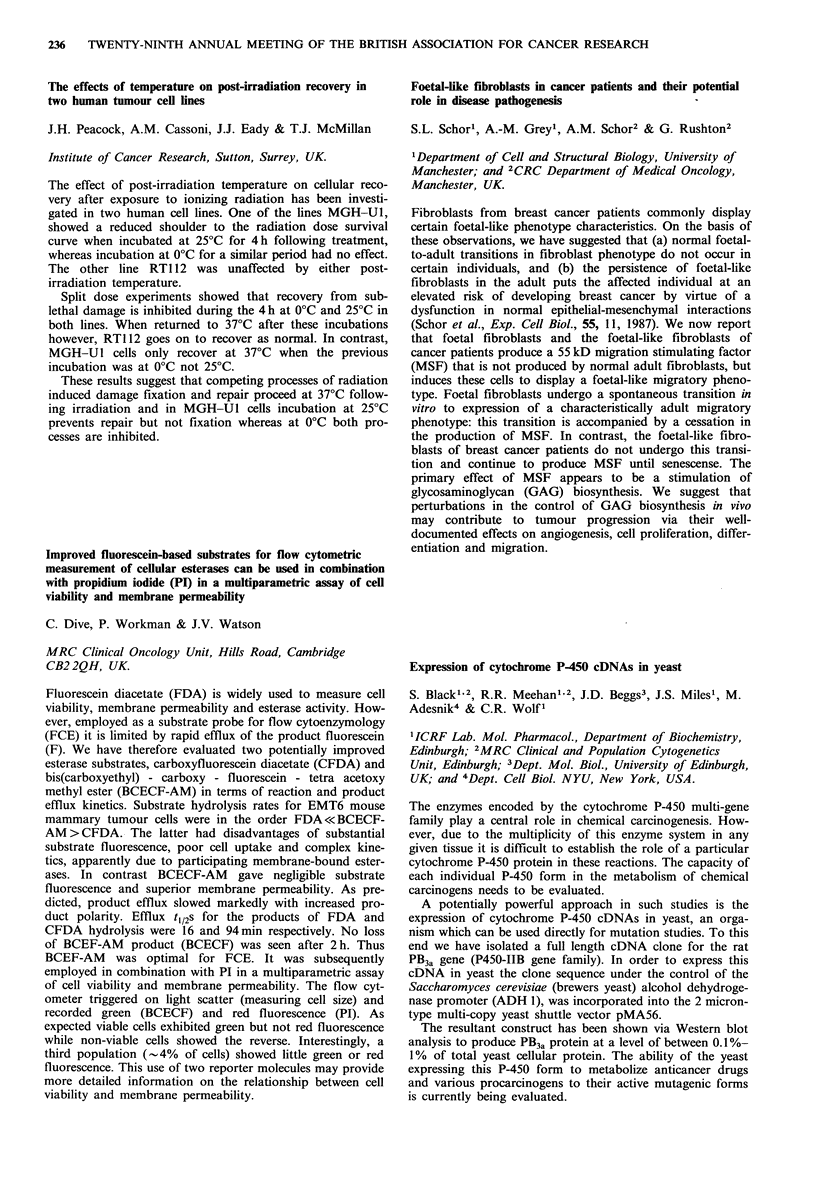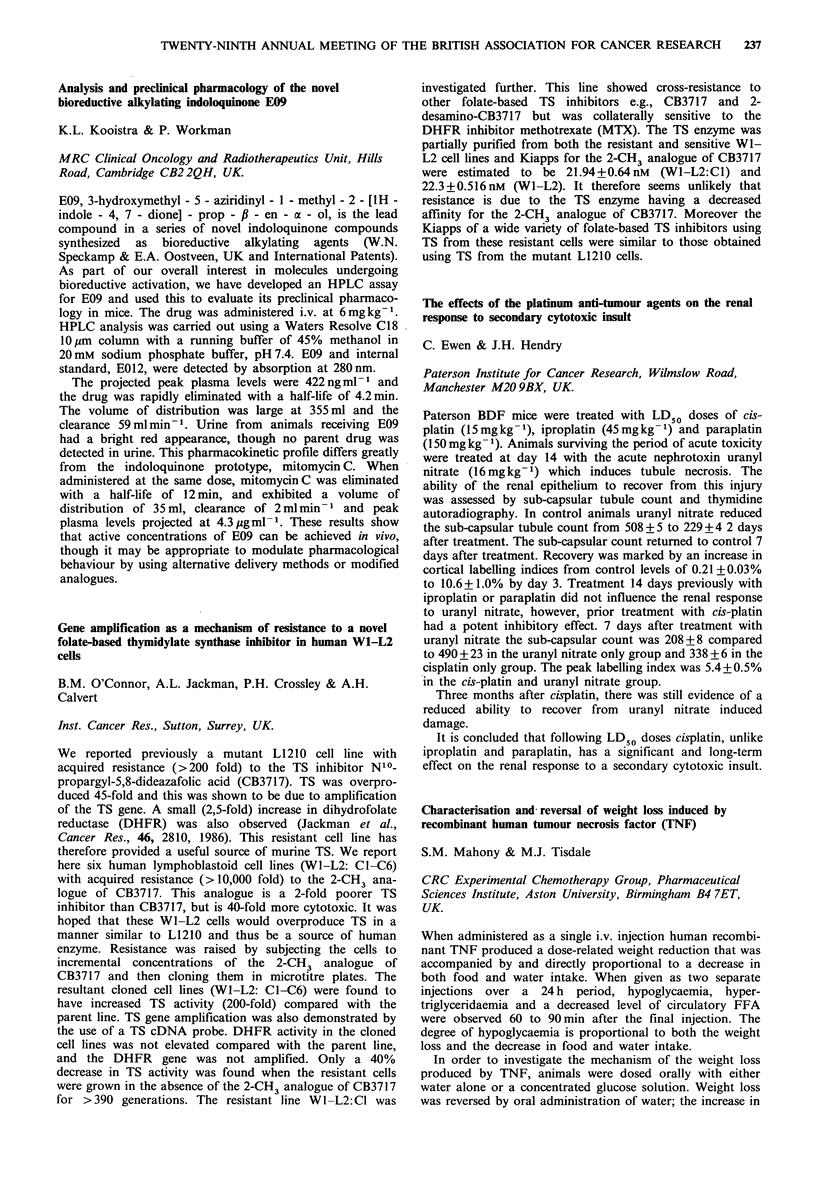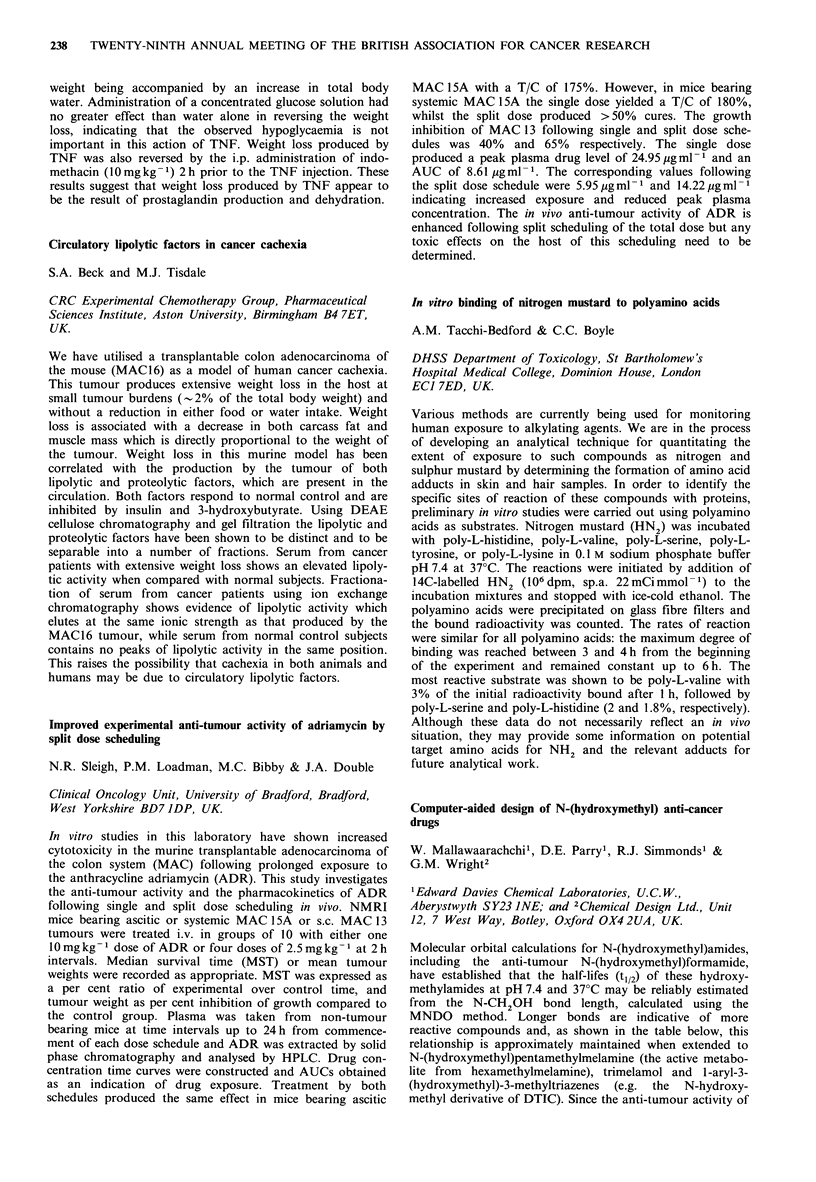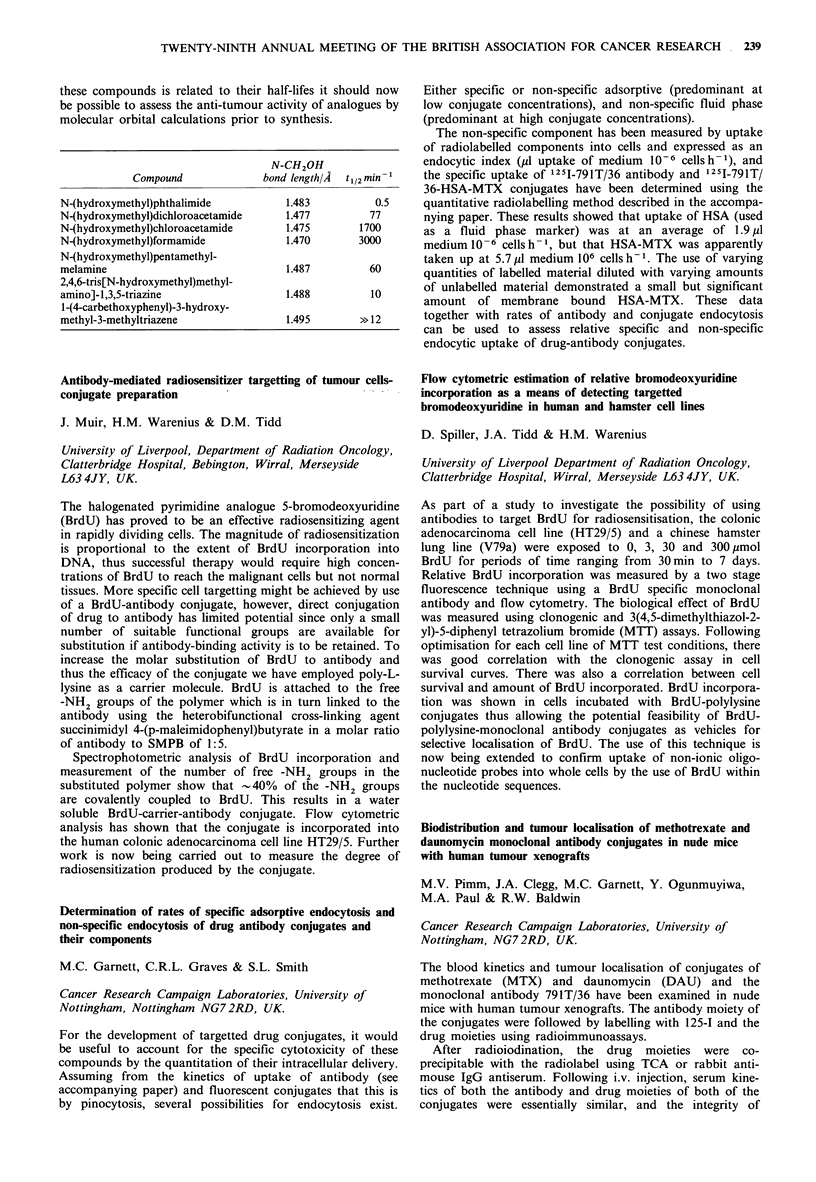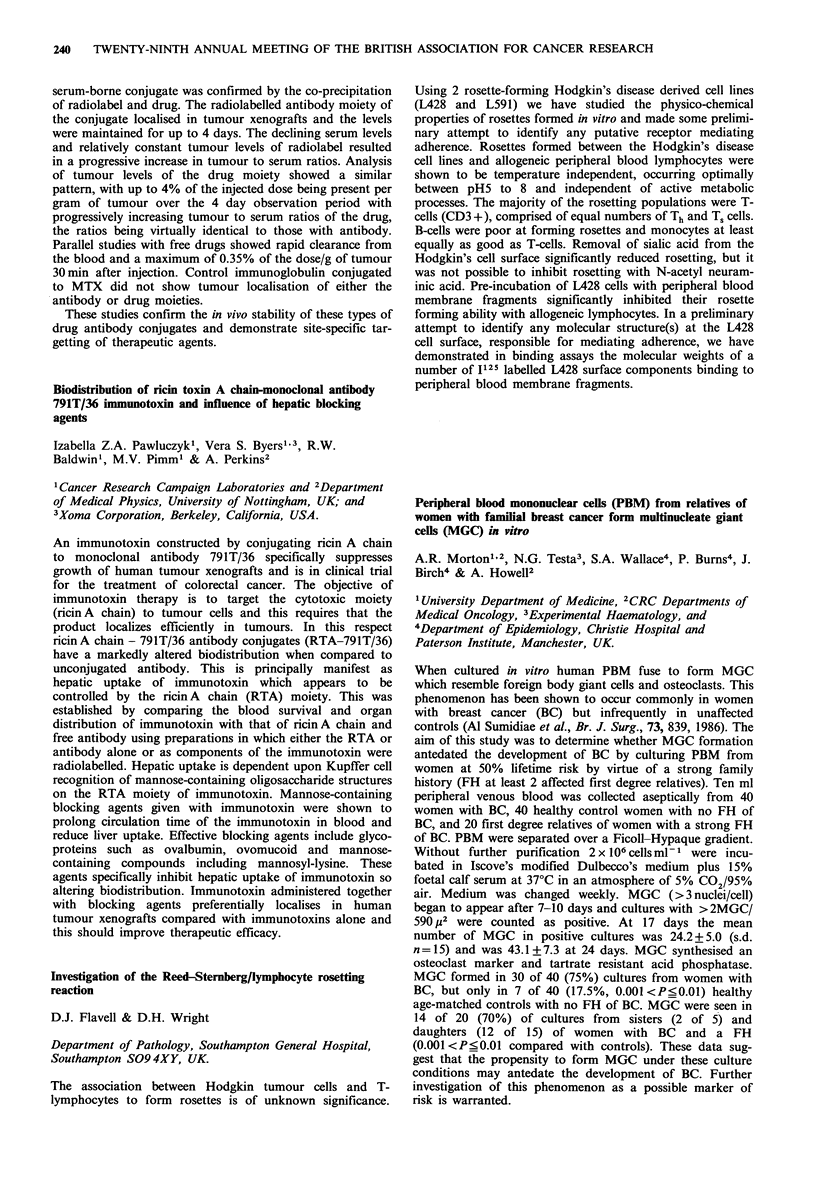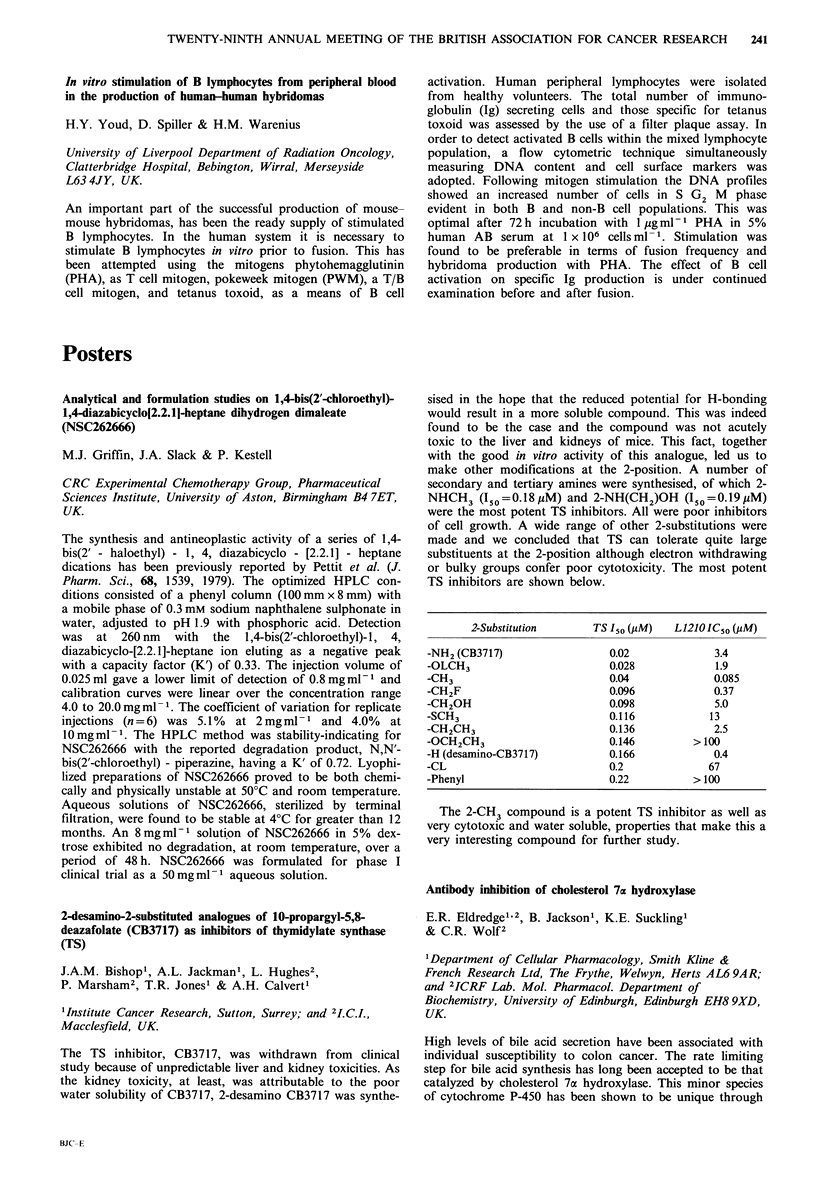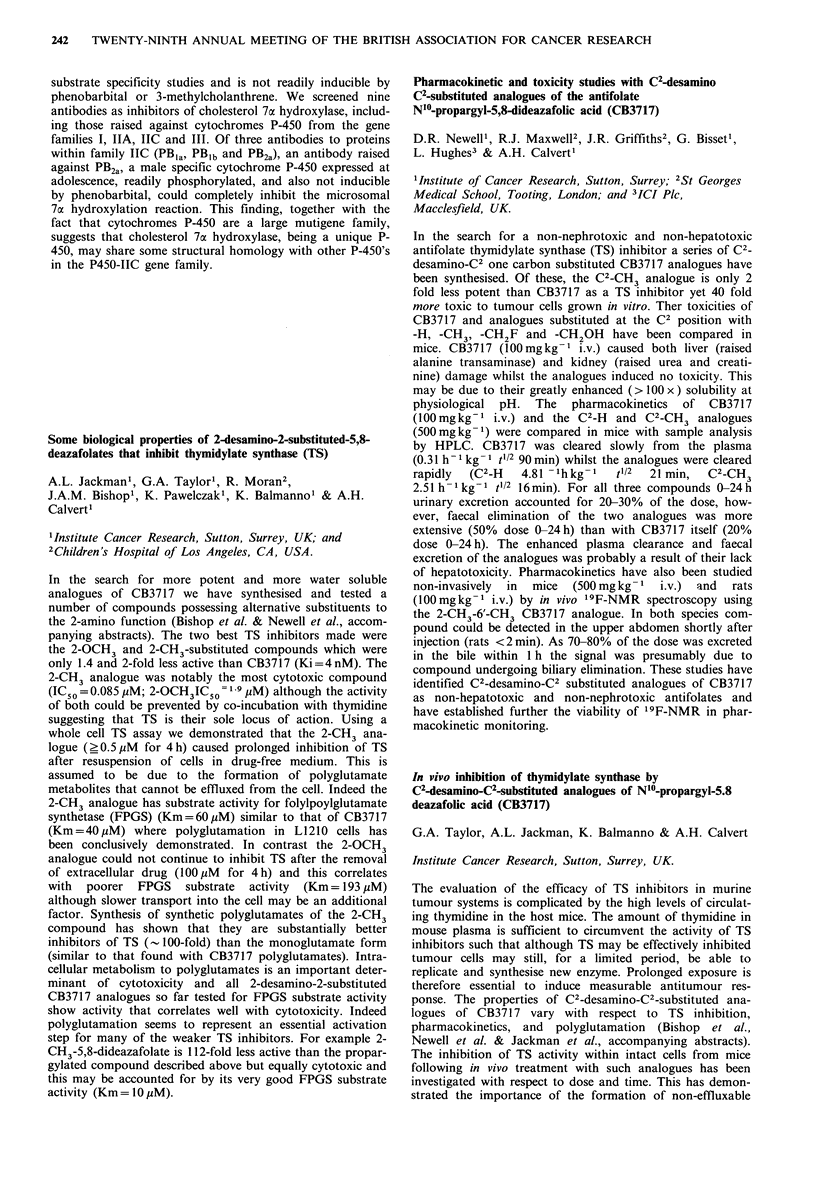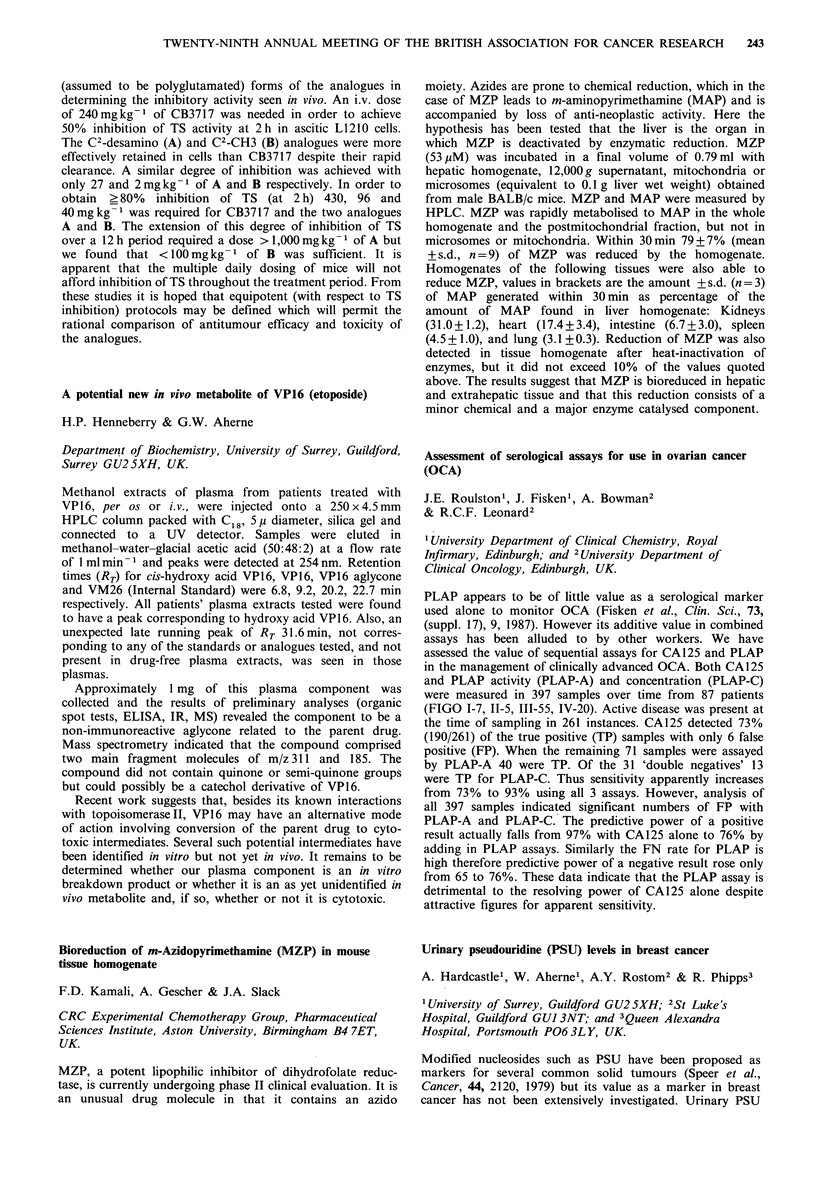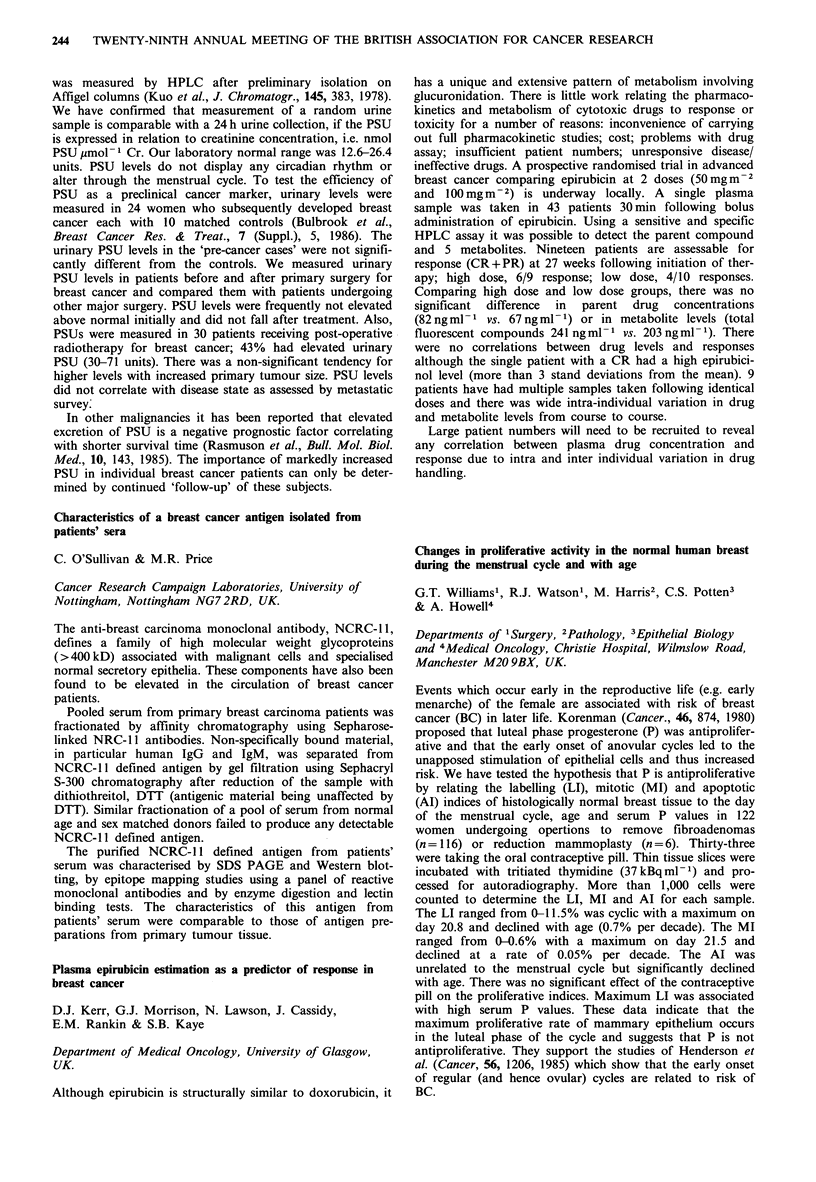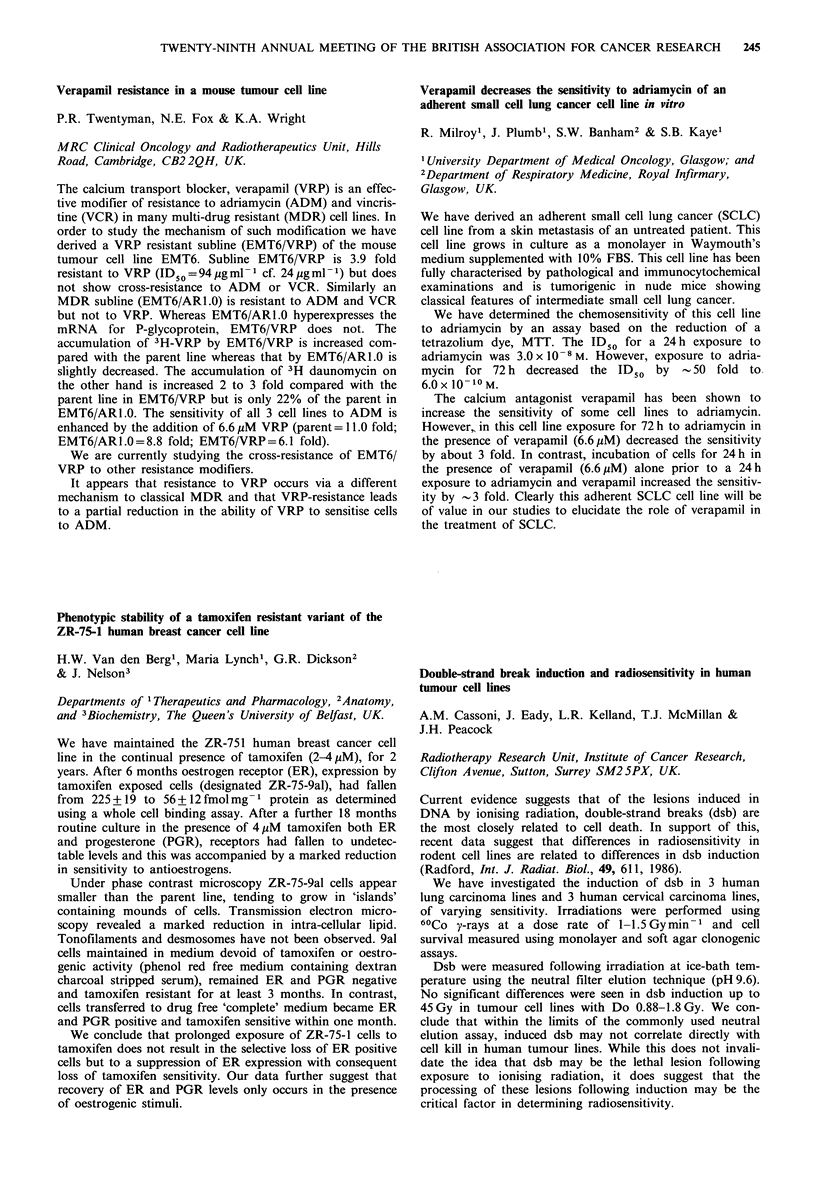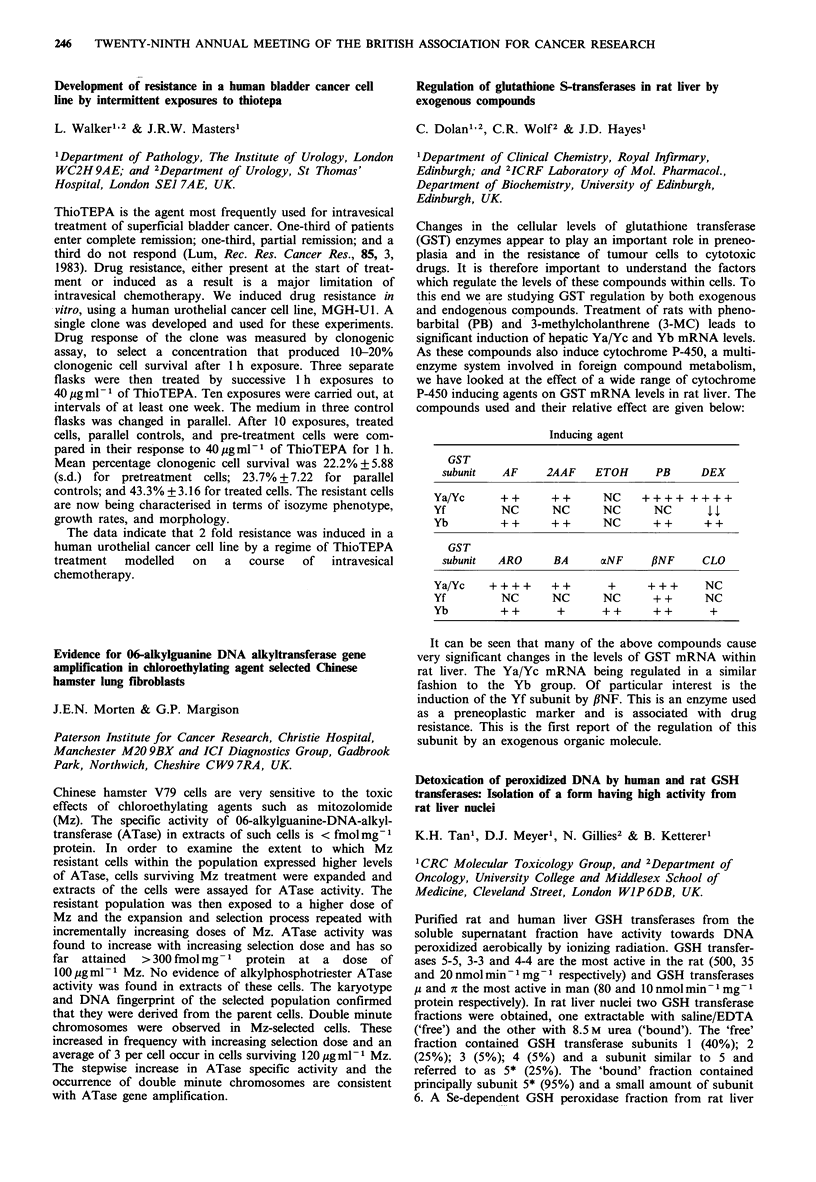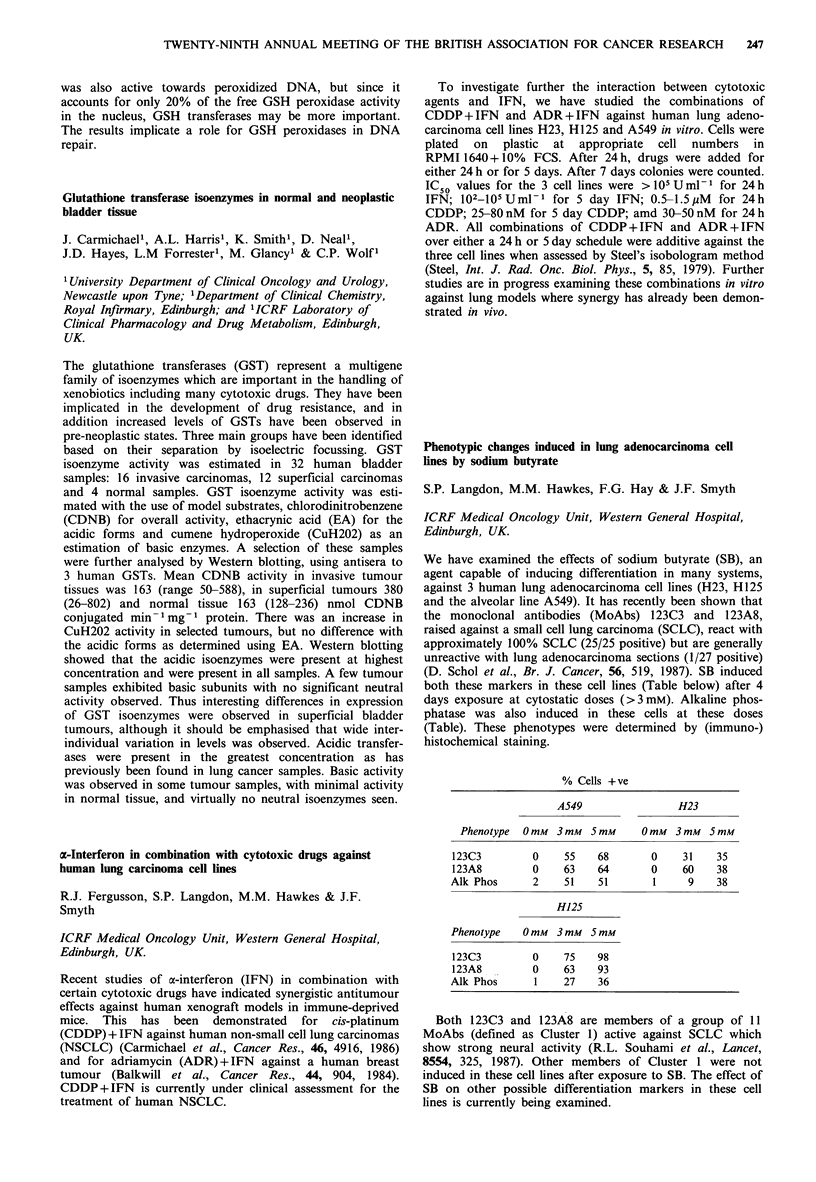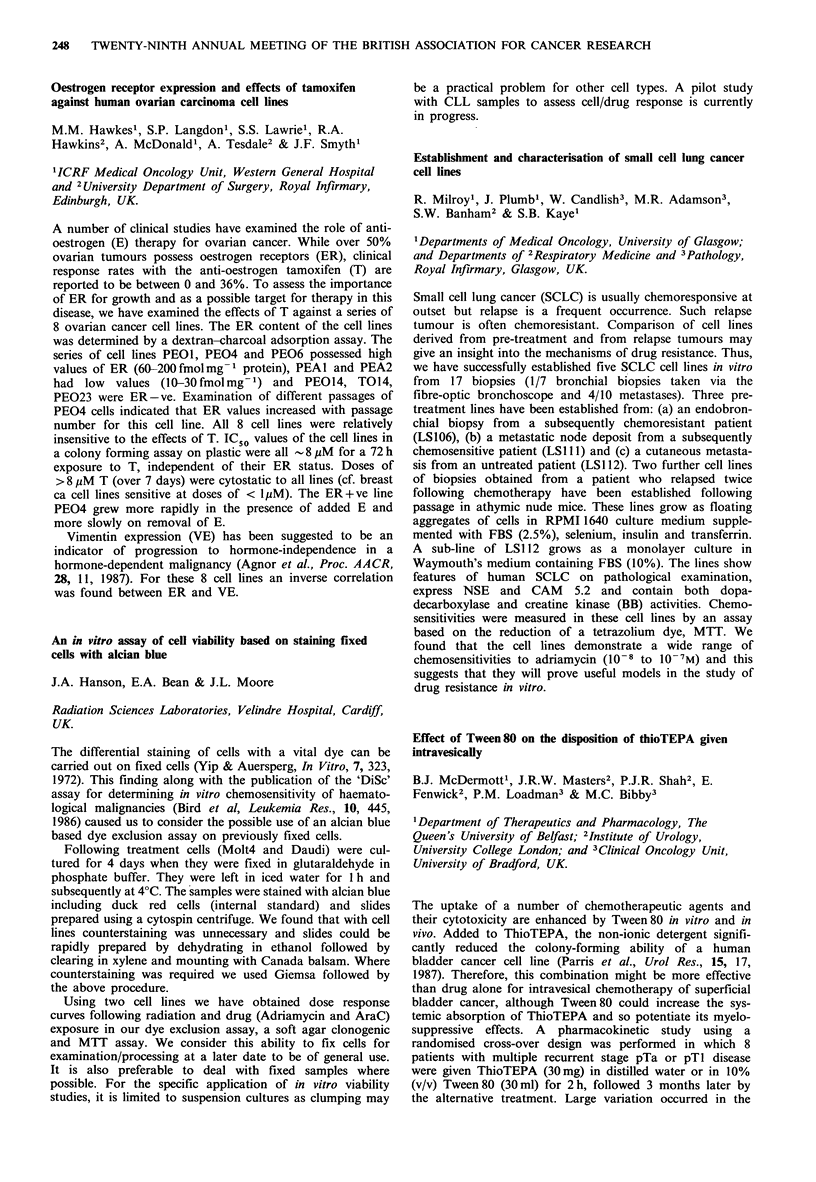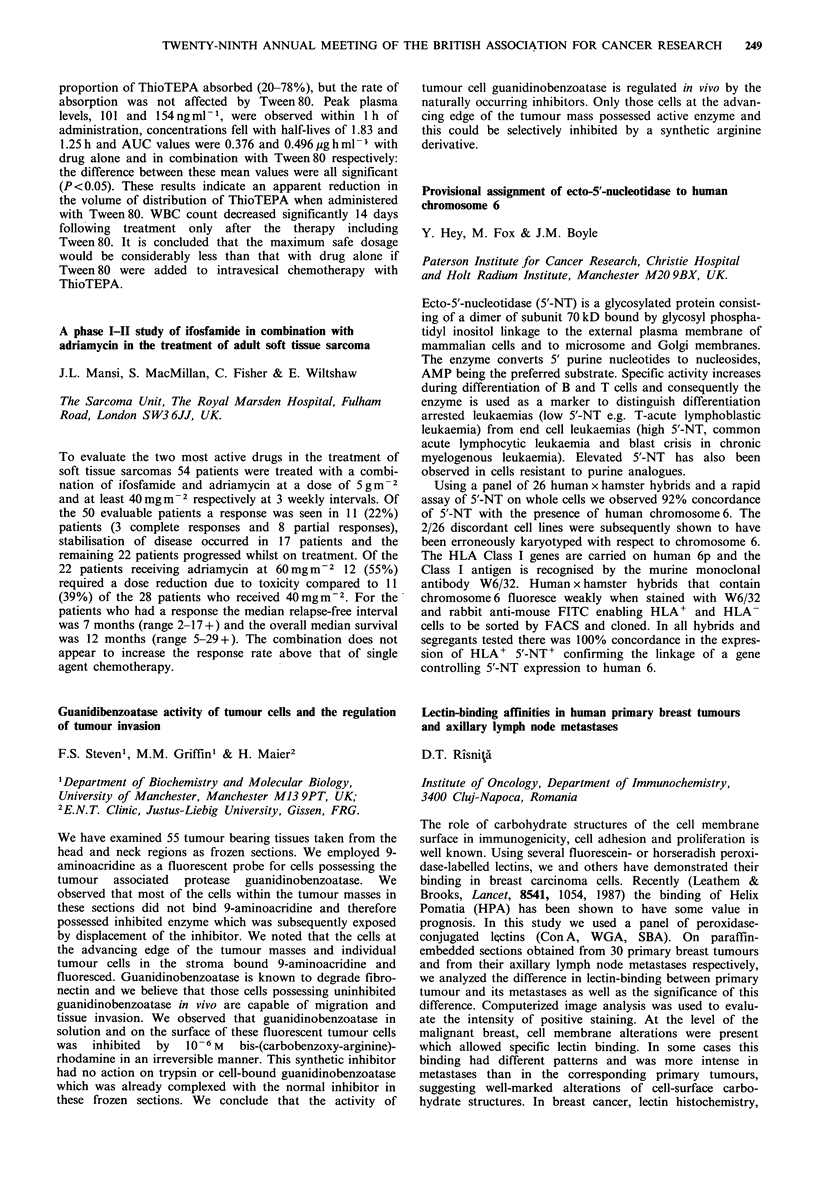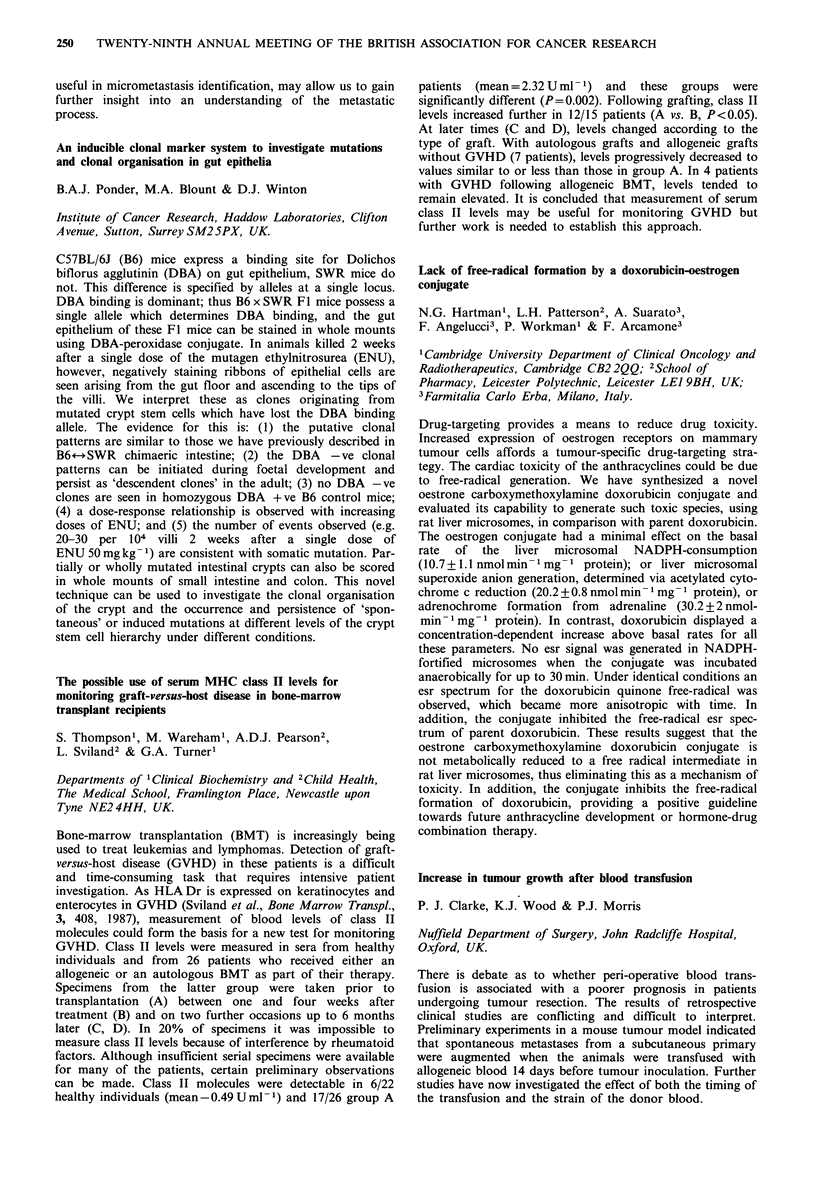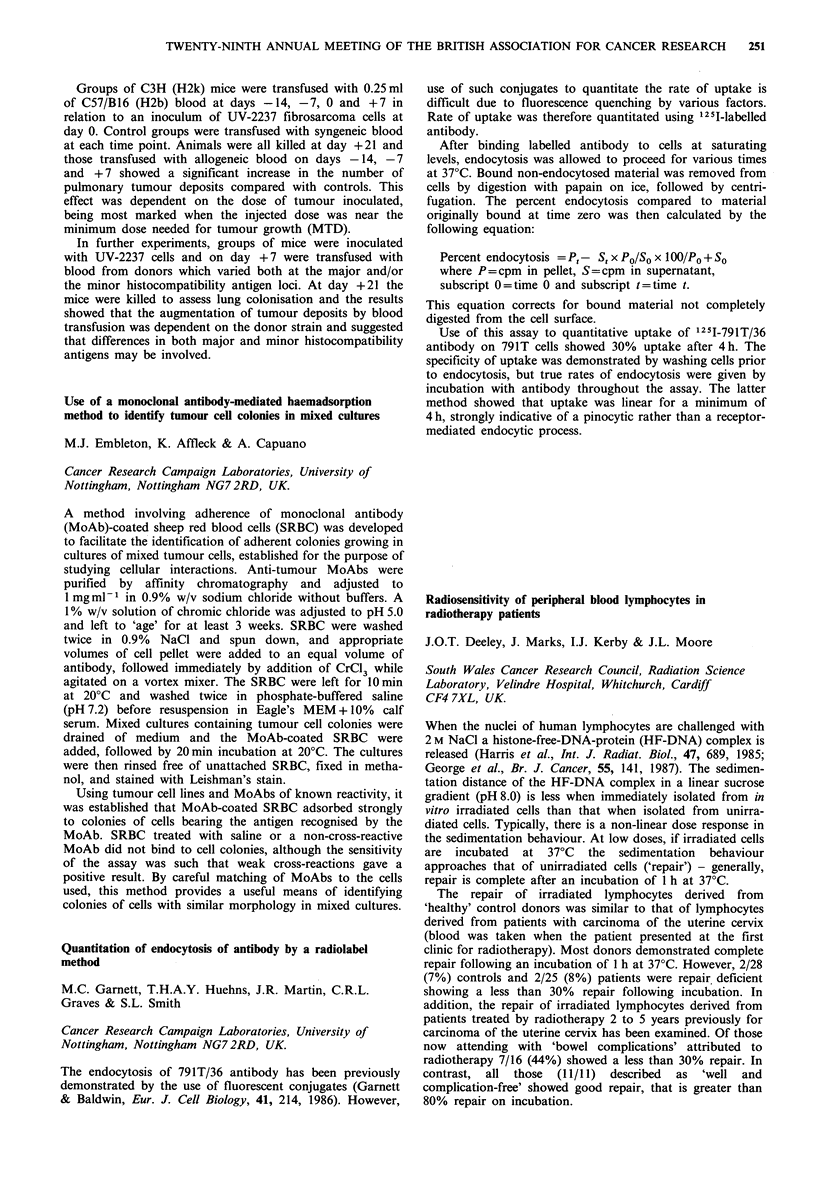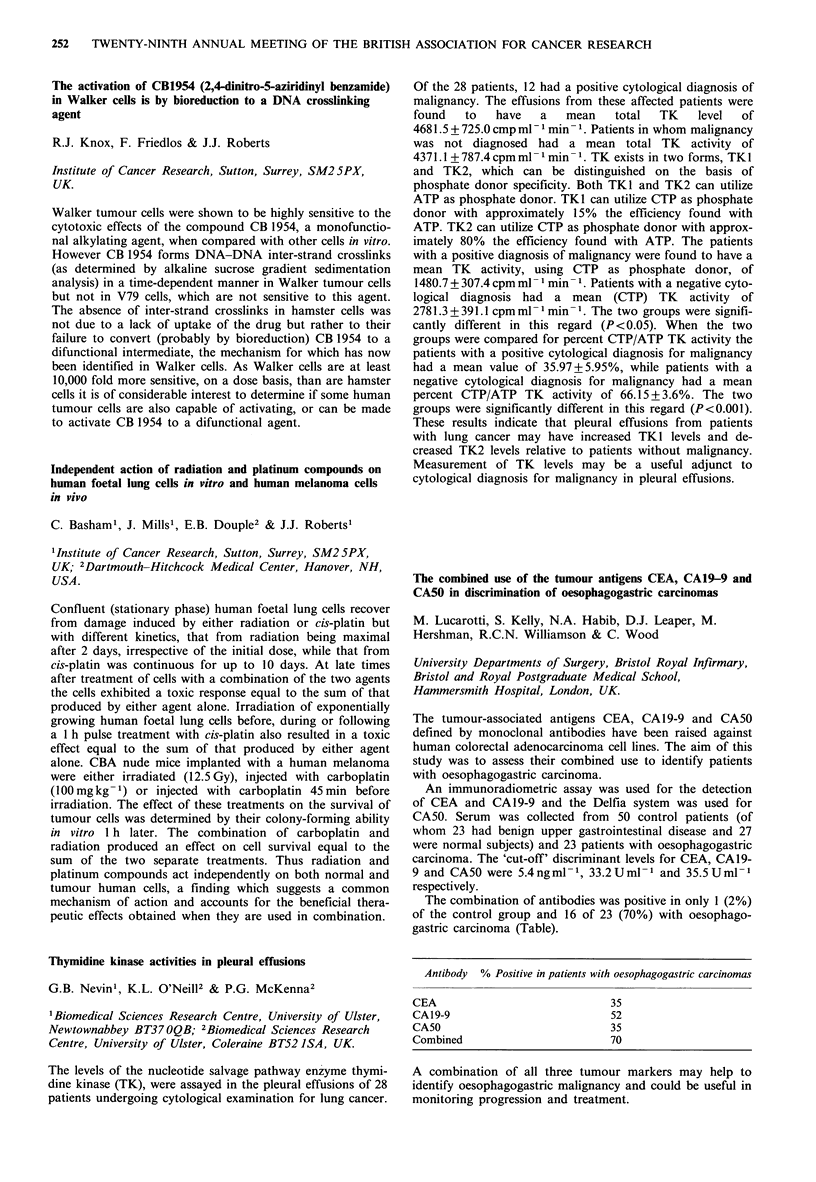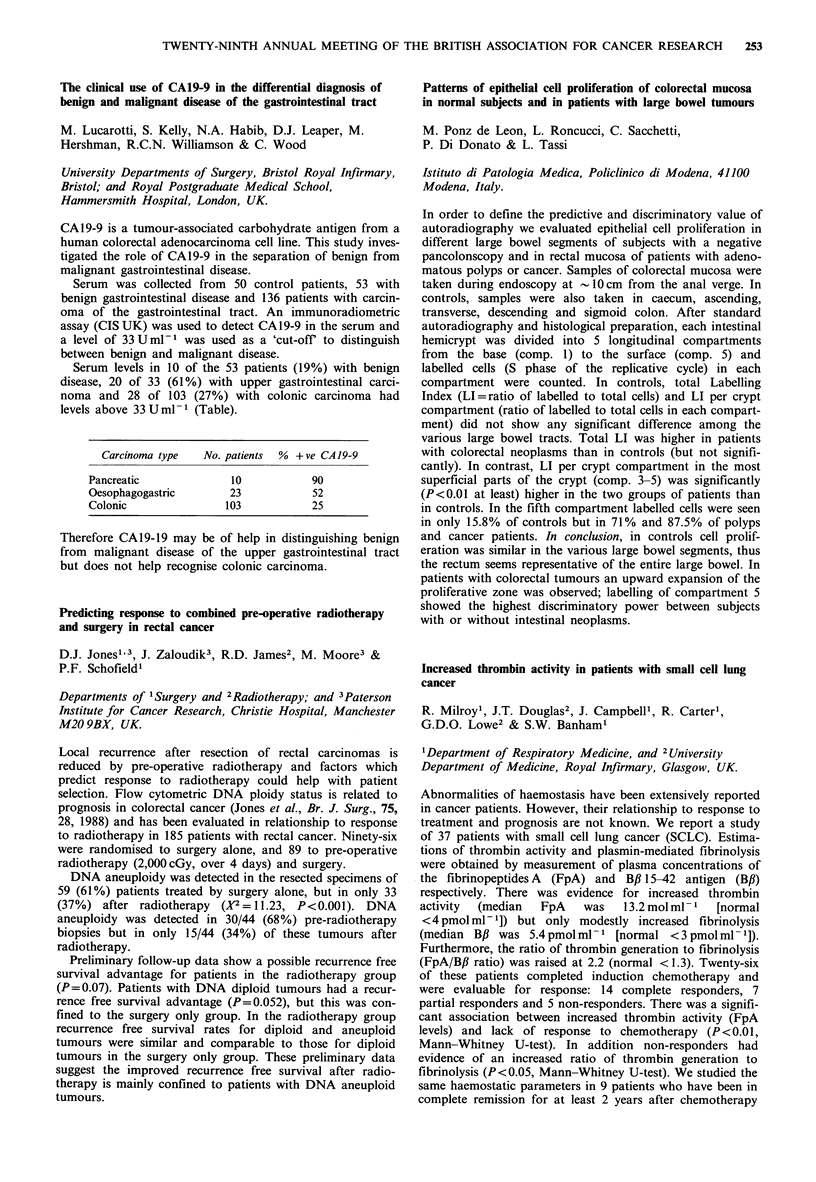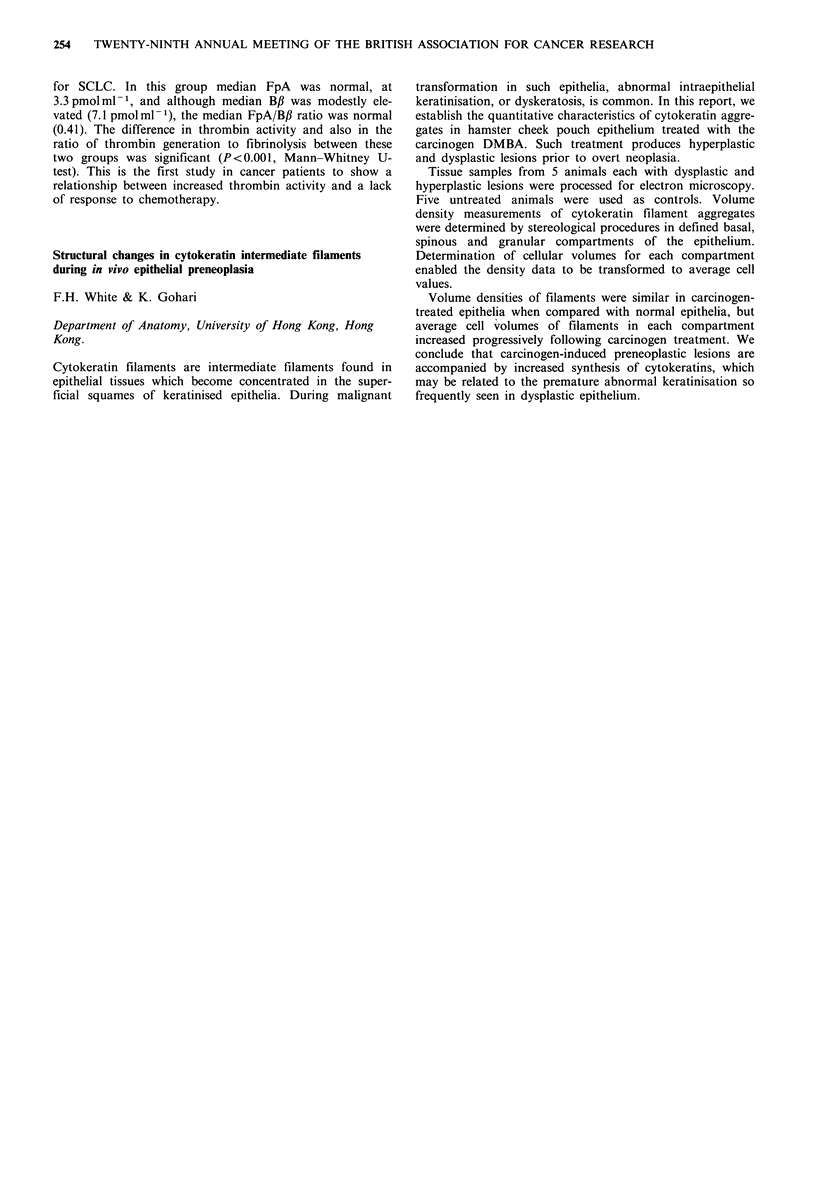# Twenty-ninth annual meeting of the British Association for Cancer Research in conjunction with the third annual meeting of the Association of Cancer Physicians. March 21-24, 1988, Norwich, U.K. Abstracts.

**Published:** 1988-08

**Authors:** 


Br. J. Cancer (1988), 58, 223–254 © The Macmillan Press Ltd., 1988

### Twenty-ninth Annual Meeting of the British Association for Cancer Research* in conjunction with the Third Annual Meeting of the Association of Cancer Physicians

(Incorporating Symposia on 'The Cell Biology of Tumour/Stroma Interactions', 'Control of Gene Expression by Sequence Recognition' and the 1988 Walter Hubert Lecture) March 21–24, 1988

*Held at the University of East Anglia, Norwich, UK.*

### Abstracts of invited papers†

### Symposium on The Cell Biology of Tumour/ Stroma Interactions

### Extracellular matrix influence on gene expression: Is structure the message?

Mina J. Bissell

*Lab Cell Biology, Div. of Biology & Medicine, Lawrence Berkeley Laboratory, Berkeley, CA 94720, USA.*

Mammary epithelial cells from midpregnant mouse have been used as a model to show that extracellular matrix (ECM) and some of its components (laminin, and heparan sulfate proteoglycan) play a fundamental role in regulation of milk protein synthesis and secretion. Using mouse and human c-DNA clones as well as polyclonal and monoclonal antibodies to the casein gene family ($\alpha$, $\beta$ and $\gamma$), transferrin, whey acidic protein and $\alpha$-lactalbumin, we have shown that the ECM regulates milk proteins at multiple points: (a) mRNA level; (b) protein synthesis and accumulation; (c) secretory rate and route; (d) the level and the nature of proteolytic activities, (3) hormonal inducibility and (4) lumen formation and vectorial secretion. Thus, a complex interplay between hormones, ECM and 'shape' determine whether or not a given protein is synthesized and how it is secreted. The results are consistent with a model of 'dynamic reciprocity' where ECM is proposed to influence gene expression via transmembrane proteins attached to cytoskeleton-in turn with connections to the nuclear matrix. A disruption of this interaction at any regulatory point will lead to destabilization of the differentiated state and may be a promotional event in cancer induction.

### Extracellular matrix in intestinal morphogenesis and differentiation

P. Simon-Assmann, F. Bouziges, K. Haffen & M. Kedinger

*INSERM Unité 61, Avenue Molière 67200 Strasbourg, France.*

Intestinal organogenesis and differentiation are driven by interactions between the endoderm and the mesenchyme. The developmental pattern of heterospecific or heterotopic recombinants between epithelial-mesenchymal anlagen allowed us to elucidate the respective role of both tissue components in morphogenesis, epithelial differentiation and hormone-elicited responses. The endoderm is induced by the mesenchyme to differentiate into the polarized epithelial cell types characteristic of the intestine. Intramucosal fibroblasts of the differentiated intestine retain properties similar to embryonic mesenchyme.

Among the mechanisms of tissue interactions, the possible mediation of information via the extracellular matrix is postulated. Positive arguments are brought about by the observation of compositional changes in matrix molecules during intestinal development and differentiation. Immunocytochemistry shows that basement membrane (BM) components are present at the epithelial-mesenchymal interface early in embryonic development and that changes in the spatial distribution of some extracellular matrix proteins within the mesenchyme are associated with morphogenetic processes. Cocultured or transplanted intestinal epithelial-mesenchymal cell associations allowed us to show (1) that cell contacts between both cell populations are essential for deposition of BM components in a polar fashion at their interface; (2) the major role of the mesenchyme in the deposition of type IV collagen in BM and in remodelling of the glycosaminoglycans (GAG), mainly of hyaluronic chains.

The question whether epithelial cells in tumours synthesize different rates of ECM molecules or induce the adjacent mesenchymal cells to modify their synthetic pattern has been tested. Using human HT29 colon carcinoma cells, we could show that the shift from undifferentiated (sugar-containing medium) to differentiated cells (sugar-free medium) was accompanied by (1) an enhancement in the overall GAG synthesis; (2) the appearance of a new class of chondroitin, Cs4 sulfate; (3) a modification in the charge density of the heparan sulfate molecule. No major modifications in the synthetic properties of the mesenchymal cell compartment were obvious when HT29 cells were cocultured with skin fibroblasts.

Taken together, the different experiments stress the importance of the mesenchymal compartment in tissue interactions which take place in intestinal development and differentiation. The search for perturbations of the epithelial-mesenchymal interactions in cancer is currently under study.

### Keratin expression and epithelial cell differentiation

E.B. Lane

*Cell Structure Laboratory, ICRF Clare Hall Laboratories, Blanche Lane, South Mimms, Potter's Bar, Hertfordshire EN6 3LD, UK.*

Monoclonal antibodies can contribute an extra level of

*Enquiries to the BACR Secretariat, c/o Institute of Biology, 20 Queensberry Place, London SW7 2DZ, UK.

†Reprints of these abstracts are not available – Ed.

resolution to histology, in that the use of certain monoclonals in immunohistochemistry will sometimes reveal subpopulations of physiologically distinct cells within what appears to be a homogeneous tissue by conventional histological staining. Recent observations show that this is particularly true of epithelial tissues. We are currently applying monoclonal antibodies which are specific for single keratin intermediate filament proteins, as they become available, to an analysis of keratin expression *in situ* in the study of epithelial differentiation. Expression of any pair or pairs of type I + type II keratins, of the 30 or so keratins described in human tissues, is characteristic of a particular state (or states) of differentiation, and intermediate filament expression appears to be one of the earlier decisions made after induction of differentiation during the course of developmental organogenesis. In complex stratified epithelia we have observed that keratin expression also changes during the course of regeneration after wounding, and that keratin expression in tumours frequently resembles that of wound-healing situations. The data suggest that the intermediate filament phenotype is fundamental to tissue function, perhaps because of a dominant effect on tissue structure. The major question regarding intermediate filaments is still that of the evolutionary pressure which could drive the establishment of such extensive heterogeneity within this gene family: How do these filaments work? To look for functional differences between keratins and to investigate how tissue specificity might be effected, we are using bacterially-produced recombinant keratin fragments to investigate keratin subdomains, using mapped monoclonal antibodies as probes for defined regions of the keratin molecules.

### Structure and function of the cytoskeletal proteins alpha-actinin and vinculin

D.R. Critchley, G.J. Price, P. Jones & M.D. Davison

*Department of Biochemistry, University of Leicester, Leicester LE1 7RH, UK.*

Vinculin and alpha-actinin are cytoskeletal proteins found in a family of specialised cell-cell and cell-substrate contacts called adherens junctions where they serve (along with a number of other components) to link microfilamentous actin to the membrane. Much of the interest in these structures stems from the observation that the interactions between the various cytoskeletal proteins is disrupted following tumour-virus transformation or exposure of cells to tumour promoters or growth factors. A detailed understanding of the mechanisms underlying these events has been limited by a lack of information on the structure of the cytoskeletal proteins involved. We have now determined the sequence of chick alpha-actinin (Baron *et al.*, *J. Biol. Chem.*, **262**, 17623, 1987) and vinculin (Price *et al.*, *Biochem. J.*, **245**, 595, 1987) and have begun to identify the functional domains in these two proteins. Thus residues 1–240 of alpha-actinin contain the actin binding domain; 240–740 contains four 120 residue repeats which show homology to those in the cytoskeletal protein spectrin; the C-terminus of the protein contains two EF-hand calcium binding sites. Interestingly, the Duchenne muscular dystrophy protein contains a homologous actin-binding domain and spectrin-like repeats. Analysis of the deduced vinculin sequence shows that residues 259–589 consist of three 112 amino acid repeats contained within the globular head region of the molecule which is released by V8 proteinase as a 90 kDa-fragment. This fragment contains the N-terminus of vinculin and the talin-binding domain. The V8 cleavage site (Leu 858) is contained within a proline-rich region which separates the vinculin head from the extended tail. There is a candidate tyrosine phosphorylation site (Tyr 822) at the head/tail junction.

### Symposium on 'Control of Gene Expression by Sequence Recognition'

*Sponsored by Bristol–Myers Oncology UK Ltd.*

### Sequence recognition by antibiotics and its structural consequences

M.J. Waring

*Department of Pharmacology, University of Cambridge, Hills Road, Cambridge CB2 2QD, UK.*

Echinomycin and other members of the quinoxaline group of antibiotics bind to DNA by a mechanism of bifunctional (*bis*-) intercalation. Footprinting experiments have revealed that they associate preferentially with sequences in natural DNA containing the dinucleotide step CpG, though this requirement cannot be mandatory because binding also occurs to poly dG.poly dC and to poly (dA-dT). The molecular basis of sequence recognition is indicated by X-ray crystallographic studies. Features of the antibiotics which are critical for recognising the preferred base-pair sequences include peptide N-methylation as well as the CO and NH functionalities of the L-alanine residues in the octapeptide ring. Sequences flanking the antibiotic binding sites are subject to conformational changes which can be seen in footprinting patterns and by enhanced reactivity to chemical reagents; they may involve Hoogsteen base pairing.

Addition of echinomycin to reconstituted nucleosome core particles containing labelled DNA produces striking changes in the nuclease digestion pattern. Strong bands, representing new nuclease-sensitive sites, appear which cannot be attributed to simple displacement of DNA from the protein core. Fourier analysis of the perturbed digestion pattern suggests that antibiotic binding causes a shift in the positioning of DNA with respect to the histone octamer, equivalent to rotation of the entire polynucleotide by about half a turn. This change in rotational orientation occurs with eukaryotic as well as prokaryotic DNA fragments and can be seen after binding of other, non-intercalating, antibiotics such as netropsin and distamycin.

### Development of sequence specific DNA effectors

J.W. Lown

*Department of Chemistry, University of Alberta, Edmonton, Alberta, Canada T6G 2G2*

Alternative approaches to the problem of developing DNA sequence specific agents for potential use in diagnosis and therapy of cancer are reviewed. The major problems of oligonucleotide probes, i.e. difficulty of cellular uptake and susceptibility to intracellular degradation, suggested as poss-

**Molecular Recognition of Lexitropsins**

ible alternatives the employment of certain oligopeptide agents. Progress in the development of lexitropsins, or information-reading oligopeptides which bind selectively to the minor groove of duplex nucleic acids, is discussed. The ability to engineer lexitropsins to recognize and bind to predetermined sequences, their ready cellular uptake, and concentration in the cell nucleus, may offer advantages in the development of cellular regulatory agents. The anticancer efficacy of prototype sequence specific minor groove alkylators is described.

### 1988 Walter Hubert Lecture

**Artificial regulation of gene expression by oligonucleotides covalently linked to intercalating agents**

C. Hélène

*Laboratoire de Biophysique, Muséum National d'Histoire Naturelle, INSERM U.201, CNRS UA.481, 61 Rue Buffon, 75005 Paris, France.*

Oligonucleotides can be used to block mRNA translation. Covalent attachment of intercalating agents to oligodeoxynucleotides provides an additional binding energy which strongly enhances hybrid formation with complementary sequences. In addition oligonucleotide-intercalator conjugates exhibit an increased penetration across cell membranes and are protected against exonucleases. These modified oligodeoxynucleotides can be used to block gene expression both *in vitro* and *in vivo*. Their effect on protein synthesis is – at least in part – mediated by an RNaseH-induced degradation of mRNA in the oligodeoxynucleotide-mRNA hybrid.

The nuclease resistance of oligodeoxynucleotides can be markedly improved by using as building blocks synthetic [α]-anomers of nucleosides instead of the natural [β]-anomers. Oligo-[α]-deoxynucleotides form double helices with complementary DNA sequences in which the two strands adopt a parallel orientation.

[α]- and [β]-oligodeoxynucleotides can be covalently attached to reactive groups which can be activated either chemically or photochemically to generate irreversible reactions in their target sequence.

The major groove of the DNA double helix can be selectively recognized by oligopyrimidines which form a local triple helix at homopurine-homopyrimidine sequences. The oligopyrimidine is oriented parallel to the purine-containing strand. Irreversible reactions can be achieved on the native DNA double helix. This opens the way for selective regulation of gene expression at the transcriptional level.

**Control of gene expression by oligonucleoside methylphosphonates**

P.S. Miller, K.R. Blake, J.M. Kean, B.L. Lee, S.B. Lin, A. Murakami & P.O.P. Ts'o

*Division of Biophysics, School of Hygiene and Public Health, The Johns Hopkins University, Baltimore, MD, USA.*

Our laboratory has studied the use of antisense oligonucleoside methylphosphonates (OMPs) to control gene expression at the mRNA level in living cells. These analogs contain nuclease resistant, methylphosphonate internucleoside bonds; form stable, hydrogen-bonded complexes with complementary sequences of single-stranded RNA and DNA; and are taken up intact by mammalian cells in culture. OMPs targeted against initiation codon regions of vesicular stomatitis virus mRNAs or against the acceptor splice junction regions of two Herpes simplex type 1 precursor mRNAs specifically inhibit virus function in virus-infected cells in a sequence dependent manner. We have also prepared OMPs derivatized with 4′-N(2-aminoalkyl)-aminomethyl-4,5′, 8-trimethylpsoralen, photoactivatable crosslinking groups. These oligomers specifically crosslink with targeted mRNA upon irradiation at 365 nm and specifically inhibit mRNA translation in cell free systems at low (1–5 μM) concentrations. A psoralen-derivatized oligomer complementary to the initiation codon region of VSV N-protein mRNA inhibits VSV protein synthesis at 5 μM concentration when VSV-infected cells are irradiated in the presence of the oligomer. Because OMPs are very stable in media containing mammalian serum and are readily taken up by mammalian cells, they should be quite useful as antisense agents in cell culture experiments and possibly in experiments involving animals. It appears that psoralen-derivatized OMPS may be particularly effective as inhibitors and that they could be used to help define the cellular target(s) of the oligomers and thus the mechanism of action of the oligomers in cell culture systems.

**Synthesis and evaluation of N-*ras* anti-sense and nonsense methylphosphonate oligonucleotide analogues**

D. Tidd[1], P. Hawley[2] & I. Gibson[2]

[1]*Department of Radiation Oncology, Clatterbridge Hospital, Wirral, Merseyside and* [2]*School of Biological Sciences, University of East Anglia, Norwich NR47TJ, UK.*

Anti-sense 8-mer nonionic methylphosphonate oligonucleotide analogues have been reported to inhibit target gene expression in intact cells by virtue of their ability to (i) cross cell membranes, (ii) resist nuclease degradation, and (iii) form sequence specific hybrids with mRNA (Miller & Ts'o, *Anti-Cancer Drug Design*, **2**, 117, 1987). We are interested in using this approach as a means of investigating the relation between oncogene expression and maintenance of the transformed phenotype. A methylphosphonate 9-mer anti-sense to codons 1–3 of the human N-*ras* gene and a 'nonsense' scramble of this sequence were synthesized by both the phosphotriester analogous and the new methylphosphonamidite chemistries. The two oligonucleotide analogues were shown by MIT assay to be completely non-toxic to human HT29 cells in culture at concentrations of up to 80 μM, and in addition, we confirmed by HPLC analysis that no detectable degradation of the molecules occurred in cultures over 48 h. A phosphodiester oligodeoxynucleotide 20-mer corresponding in sequence to codons 1–7 of the human N-*ras* gene was used to investigate the hybridization properties of the methylphosphonates in solution. At low temperature ($<5$°C) both anti-sense and nonsense analogues formed 1:1 complexes with the 20-mer probe, but these were largely dissociated at 25°C. Only a fraction (10–20%) of the molecules in the anti-sense preparations formed stable hybrids with the 20-mer. This could conceivably mean that most of the 256 diastereoisomeric forms present in the purified 9-mer were unable to form Watson–Crick base pairing interactions along their entire length. The melting temperature of the hybrids which did form was approximately 34°C. Our preliminary cell culture results were not repeated in subsequent experiments, which showed no effect of the anti-sense oligonucleotide on induction of $p21^{N\text{-}ras}$ protein synthesis by dexamethasone in T15 cells, a line of NIH 3T3 cells transfected with multiple copies of the human N-*ras* gene under control of the MMTV promoter.

### Pyrrolo(1,4)benzodiazepine antitumour antibiotics. Sequence specificity and effect of binding on local DNA structure

L.H. Hurley & L. Boyd

*Drug Dynamics Institute, College of Pharmacy, University of Texas at Austin, Austin, TX 78712, USA.*

Anthramycin and tomaymycin are members of the pyrrolo-(1,4)benzodiazepine antibiotic group. These drugs bind covalently in the minor groove of DNA through N2 of guanine. Previous studies have shown that PuGPu sequences are the most favoured for binding, while PyGPy sequences are least favoured. Using a combination of fluorescence, $^{1}H$ and $^{31}P$-NMR, and molecular modelling studies we have examined the binding of tomaymycin and anthramycin to various oligomers including $d(ATGCAT)_2$. While anthramycin only forms one type of adduct with the duplex, tomaymycin forms two adducts in approximately equal amounts. Anthramycin binds via a 11S linkage geometry with the aromatic ring to the 3′ side of the covalently modified guanine, while tomaymycin binds with either an 11S or 11R linkage geometry. Molecular modelling and $^{1}H$-NMR studies suggest that the 11S isomer binds in the 3′ orientation while the 11R isomer binds in the 5′ orientation. Upon binding of anthramycin to DNA the covalently modified strand undergoes a selective conformational change up to three bases to the 3′ side of the adduct. At the covalent linkage site the glycosidic dihydral angle is dramatically changed and the pucker of the sugar to the 3′ side assumes the unusual C4′-endo conformation. The sugar-phosphate backbone torsion angles between the covalently modified guanine and the two bases to the 3′ side also appear to have undergone changes resulting in significant changes in the base to H1′ and H2′ and H2″ distances and downfield shifts of the $^{31}P$-NMR signals.

### New DNA-interactive molecules: Rational design by computer modelling

T.C. Jenkins, R. Newman, L.H. Pearl, A. Walton, G.D. Webster & S. Neidle

*CRC Biomolecular Structure Unit, The Institute of Cancer Research, Sutton, Surrey SM2 5PX, UK.*

Computer graphics modelling and molecular mechanics calculations are powerful tools for studying intermolecular interactions. We have been applying these techniques to the design of analogues of the DNA groove binding molecule berenil which have potentially altered sequence recognition properties. The scope and limitations of the approach will be discussed, and results compared with those obtained experimentally.

### Abstracts of Members' Proffered Papers – Oral presentations

### NIH-3T3 cells can be transformed by transfection with DNA from transplanted rat hepatomas but not primary rat hepatomas

M.J. Embleton, A. Stibbe & P.C. Butler

*Cancer Research Campaign Laboratories, University of Nottingham, Nottingham NG7 2RD, UK.*

NIH-3T3 cells were transfected with DNA extracted from chemically-induced primary and syngeneically transplanted rat hepatomas using a calcium phosphate co-precipitation method, in order to assess the ability of the DNA to induce transformation. Negative control DNA was prepared from NIH-3T3 cells and normal rat liver, and a positive control was provided by EJF-5, a line of NIH-3T3 transformed by DNA from human bladder carcinoma cells. The EJF-5 DNA and DNA from two transplanted rat hepatomas originally induced by oral 4-dimethylaminoazobenzene (DAB) produced transformation of NIH-3T3 recognised as piled-up foci in monolayer cultures and by clonogenicity in soft agar, while liver DNA and NIH-3T3 DNA produced none at all. DNA from four primary hepatomas induced by DAB and five induced by oral 2-acetylaminofluorene failed to transform NIH-3T3. These DNA preparations were able to transfer the APRT+ phenotype into LM clone 11 APRT− cells, however, so they were active with respect to other genes. It was concluded that transforming genes were not detectable in the DNA of primary rat hepatomas, but became expressed as part of the progression or selection process accompanying repeated transplantation. It is suggested that the more vigorous growth (hence more 'malignant'behaviour) of such selected cell populations may be associated with greater expression of activated oncogenes in comparison with the mean level in primary tumours.

### Suppression of tumorigenicity in cells transfected with DNA from non-neoplastic cells

D. Tarin[1], A. J. Hayle, J. Taggart[1] & P.A. Whittaker[2]

*[1]Nuffield Department of Pathology, John Radcliffe Hospital, Headington, Oxford OX3 9DU and [2]Department of Biochemistry, University of Oxford, UK.*

This study investigated whether transfer of DNA from non-neoplastic cells into tumour cells could suppress tumour formation, by supplying regulatory genes, postulated to be deleted or damaged in malignant cells. Total genomic DNA from normal human blood leucocytes was co-transfected with the gene for neomycin resistance ($neo^R$) into the highly tumorigenic mouse fibrosarcoma cell line (TR4 Nu). Controls consisted of cells co-transfected with their own DNA, or with DNA from various human and animal tumour lines and $neo^R$. Clones surviving after selection in neomycin were expanded separately and inoculated s.c. into nude mice. These were observed until tumours arose or for 180 days, whichever was sooner. Non-transfected clones of TR4 Nu all (24/24) formed s.c. tumours with a mean latent period of 28 days. Similarly rapid 100% tumorigenicity was observed for the various control clones transfected with mouse or human tumour DNA (41/41). However, of the 34 clones tested, transfected with *normal* human DNA, one (XD1.6) exhibited marked inhibition of tumorigenicity and was demonstrated to contain exogenous DNA by Southern blotting and probing with $neo^R$ and with a probe for human repetitive sequences (Blur 8). The earliest tumour arising was not observed until 100 days while some animals did not develop tumours at all within the 180 day observation period. The mean latent period of tumorigenicity among animals that did develop tumours was 127 days. These observations indicate that tumorigenicity can be suppressed or inhibited by sequences transferred from the normal genome. It has not escaped our notice that these findings, if corroborated, have therapeutic implications.

### Characterisation of clonal cell populations after transfection of metastatic DNA

A.J. Hayle[1], P.A. Whittaker[2] & D. Tarin[1]

[1]*Nuffield Department of Pathology, John Radcliffe Hospital, Headington, Oxford OX3 9DU and* [2]*Department of Biochemistry, University of Oxford, UK.*

Transfection of total genomic DNA from the highly metastatic mouse histiocytic sarcoma (M5076) line into a non-metastatic mouse fibrosarcoma cell line (TR4 Nu), resulted in the establishment of clones which showed the capability to overwhelmingly colonise the lungs when inoculated i.v. into nude mice. Of the 5 clones tested, one produced metastatic colonisation of the lungs in 17/22 (77%) nude mice tested, another clone resulted in 16/21 (76%) showing colonisation, whilst the remaining 3 clones showed 6/22 (27%), 5/23 (21%) and 0/22 animals with metastatic lesions. One clone was also spontaneously metastatic in 1/8 (12.5%) nude mice tested from a tumour formed after s.c. inoculation. Since the experiment involved transfection of mouse cell DNA into another mouse cell line, the DNA from the donor cells was first cloned into a cosmid library to aid recognition and recovery of transferred sequences. The cosmid used (PCV108) also contained the gene for resistance to neomycin (*neo*$^R$) as a selectable marker. Transfections were performed using polybrene and DMSO and survival of cell clones in neomycin containing medium provided initial evidence of exogenous DNA incorporation. By using nick translated isolated *neo* sequences to probe Southern blot transfers of the DNA from these clones, we have confirmed the presence of cosmid vector sequences (and therefore by inference transfected M5076 DNA) in the transfected cells. Multiple incorporation patterns of the cosmid library were seen which were unique to specific clones. These results confirm previous findings indicating that components of the metastatic phenotype can be dominantly conferred on previously non-metastatic tumour cells by transfer of genomic DNA. Further, they may enable isolation and identification of genes believed to be involved in metastatic behaviour.

### Metastasis and the increased expression of MHC class I molecules. A possible mechanism for how the primary site could affect tumour spread

M. Blackmore[1], S. Thompson[1], C.G. Brooks[2] & G.A. Turner[1]

*Departments of* [1]*Clinical Biochemistry and* [2] *Pathology, The Medical School, University of Newcastle upon Tyne, UK.*

Interactions at the primary site of tumour growth could be very important in affecting metastasis. Recent studies (Karre *et al*., *Br. J. Cancer*., **36**, 503, 1985) have suggested that interferon-stimulated MHC expression may be linked to increased metastasis via reduced NK cell recognition. In the current study we provide further support for this proposal, and present evidence to suggest that this mechanism could be operating *in vivo* and explain the observed variation in metastasis with primary implantation site. Class I molecules (KbDb) were not detected on the surface of cultured murine melanoma cells (B16–BL6) using cell surface radiolabelling, immunoprecipitation and electrophoresis. This finding was further confirmed by screening mRNA isolated from BL6 cells with synthetic oligonucleotide probes to class I sequences. However, class I molecules were detected if the cells were grown s.c. and then reintroduced into culture. In other experiments, treatment of MHC-negative cells with IFN-$\gamma$ (10–10,000 U ml$^{-1}$) induced class I expression (as measured by immunoprecipitation), and the acquisition of sensitivity to anti-K$^b$-specific cytotoxic T cells) but caused an almost complete loss of sensitivity to syngeneic NK cells. When these cells were injected intravenously they formed twice as many lung colonies (15 control mice: median = 41; 15 treated mice: median = 80). In the s.c. site, the treated cells appeared to grow faster but this finding is very preliminary and no similar effect was observed for cells growing *in vitro*. IFN-$\alpha/\beta$ also increased class I expression but this was much less than observed with IFN-$\gamma$. These studies suggest that there may be a direct relationship between high MHC class I expression, loss of NK sensitivity and the metastatic spread of tumours in this system.

### Possible autocrine control of growth and progression of melanoma by $\alpha$-MSH

J. Lunec[1], C. Fisher[2], C. Parker[1], G.V. Sherbet[1] & A.J. Thody[2]

[1]*Cancer Research Unit, University of Newcastle upon Tyne, Royal Victoria Infirmary, Newcastle upon Tyne NE1 4LP and* [2]*Department of Dermatology, University of Newcastle upon Tyne, Newcastle upon Tyne NE1 4LP, UK.*

Melanocyte stimulating hormone (MSH) regulates pigment cells and recent evidence suggests that in the case of melanoma cells it stimulates anchorage independent growth and metastatic capacity. In this study, we have examined the possibility that melanoma cells produce MSH.

Immunoreactive $\alpha$-MSH was detected in several variants of the B16 murine melanoma. Levels ranged from $339 \pm 81$ pg $10^{-6}$ cells ($n = 9$) in BL6 cells to $445 \pm 69$ pg $10^{-6}$ cells ($n = 7$) in F10 cells. As shown by HPLC, most of the immunoreactivity corresponded to desacetyl and monacetylated $\alpha-$MSH. No immunoreactivity was detected ($<20$ pg $10^{-6}$ cells) in 3T3, A431 cell lines and in normal keratinocytes and melanocytes.

Additional evidence for the expression of $\alpha$-MSH by B16 melanomas has also been obtained at the transcriptional level. A full length cDNA probe for the murine pro-opiomelanocorticotropin (POMC) gene transcript, which includes the $\alpha$-MSH coding sequence, was used to detect the presence of POMC cDNA clones in a $\lambda$gt10 bacteriophage cDNA library made using mRNA isolated from the BL6 high metastasis variant of the B16 melanoma. Strong positive signals were obtained under high stringency hybridisation conditions at a frequency of $\sim 0.02\%$ of the clones.

Although melanoma cells are known to be responsive to $\alpha$-MSH, their ability to produce this peptide has not been previously recognised. Our observations are particularly significant in the light of recent reports that the addition of $\alpha$-MSH to cultured B16 melanoma cells can increase the metastatic capacity of these tumour lines. The results suggest that $\alpha$-MSH may be a key autocrine and possibly paracrine factor which determines the high degree of invasion and metastasis that is characteristic of the progression of melanomas.

### Gene amplification, growth factor/receptor genes and oncogenes are associated with sister chromatid exchange in melanoma and glioma cell lines

M.S. Lakshmi & G.V. Sherbet

*Cancer Research Unit, University of Newcastle upon Tyne, Medical School, Newcastle upon Tyne NE2 4HH, UK.*

Genetic recombination has been suggested as a mechanism for the appearance of the metastatic phenotype. Recombinational events, seen as sister chromatid exchanges (SCE), correlate with metastatic potential of B16 melanomas. Since unequal homologous SCEs can lead to gene amplification, we have examined the incidence of SCE and gene amplification seen as double minute chromosomes (DM) in F1 (low metastasis) and BL6 (high metastasis) variants of the B16 murine melanoma.

SCEs occurred predominantly in the hypertriploid cells of both cell lines. A high proportion of these were also DM positive. The BL6 line had 2.5× more DM+ cells than the F1 line. Hypodiploid cells showed no DMs in spite of high SCE incidence. In the human glioma GUVW, SCEs and DMs occurred markedly in hypertriploid cells. We propose that unequal SCEs occur at gene loci flanked by repetitive sequences and cause gene amplification. This postulate assumes that SCEs are a non-random event. The chromosomal distribution patterns of SCE were found to be different for human melanoma and astrocytoma cell lines. The SCE break points occurred in the proximity of chromosomal fragile sites and involved genes for growth factors/growth factor receptors and oncogenes. Thus, in the melanoma, the break points involved epidermal growth factor gene, *neu*, Ki-*ras*, *myb* and *ros*. In the glioma, *Rb1*, N-*ras* and DHFR genes were involved. In both tumour types, glucocorticoid receptor gene, $\beta^2$-microglobulin gene and its regulator gene were involved.

### The isolation of cDNA clones for cysteine proteases and measurement of the expression of corresponding genes in metastatic variants of B16 melanoma

J. Lunec, C. Parker & G.V. Sherbet

*Cancer Research Unit, The Medical School, University of Newcastle upon Tyne, Newcastle upon Tyne NE2 4HH, UK.*

Cysteine proteases such as cathepsin B possess a highly conserved amino acid sequence at the putative active site. cDNA clones for gene encoding this conserved sequence have been isolated from a B16 melanoma cDNA library using the corresponding mixed synthetic oligonucleotide probe. DNA from one of these clones, designated $\lambda$gt 10BL6–CysP–1, has subsequently been used as a probe in Northern and Southern transfer hybridisation studies to compare expression of the corresponding gene between B16 melanoma variants showing different capacities for local invasion and metastasis, and to test for possible rearrangements of the gene or alteration in copy number. Subsequent restriction mapping has identified $\lambda$gt 10BL6–CysP–1 as a cDNA clone for Cathepsin-L.

Three B16 melanoma tumour lines were analysed: B16–F1, which only metastasises to the lung at a low level; B16–BL6, which is selected for increased ability to metastasise to the lung and penetration of bladder epithelium; and B16–ML8, which is derived from lung metastases of the B16–BL6 primary, exhibits an even greater ability to metastasise to the lung but appears to be less locally invasive than either B16–BL6 or B16–F1. The Cathepsin-L probe detected a single 1.6 kb transcript in RNA samples from all of these cell lines, whether grown in culture or as the primary tumour. This transcript was expressed at an elevated level in the B16–BL6 tumour compared with B16–F1. No difference was evident when the cells were grown in culture. However, B16–ML8 was found to express the Cathepsin-L transcript at a greatly reduced level in cell culture compared with either B16–F1 or B16–BL6. This difference was not so apparent in tumour samples. These observations suggest that invasion and metastasis are dissociable phenomena and that the expression of Cathepsin-L may be associated with local invasion but not metastasis in this system.

### Expression of major histocompatibility complex (MHC) products in colorectal cancer

D.J. Jones[1,2], A.K. Ghosh[1], M. Moore[1], J.M.T. Howat[3] & P.F. Schofield[2]

[1]*Department of Immunology, Paterson Institute for Cancer Research, Christie Hospital, Manchester;* [2]*Department of Surgery, University Hospital of South Manchester; and* [3]*Department of Surgery, North Manchester General Hospital, UK.*

Lymphocytic infiltration is a favourable prognostic factor in colorectal cancer. If this phenomenon is tumour antigen related, MHC expression would be important, since functional T-lymphocyte subsets recognize cell bound 'foreign' antigen, preferentially in the presence of MHC class I and II molecules. To test this hypothesis MHC expression was studied immunohistochemically on cryostat sections of 100 prospective colorectal carcinomas, using monoclonal antibodies to class I and II molecules. Normal mucosa was studied from 64 cases.

Normal mucosa and 96% of carcinomas were class I positive. Class II positive epithelia were detected in 13% of normal sections compared to 57% of carcinomas ($X^2=30.47$, $P<0.001$). 11/12 (92%) Dukes' A class II positive compared to 46/88 (52%) Dukes' B and C ($X^2=5.17$, $P<0.025$). MHC expression was independent of the extent of infiltration.

Class II expression was also observed in 5/5 cases of inflammatory bowel disease.

The ability of epithelial cells to express class II antigens is therefore a feature of both non-neoplastic and neoplastic mucosa, but in carcinomas induction of class II expression is decreased in advanced disease and could be associated with escape from immune surveillance. However, since MHC expression was independent of the extent of lymphocytic infiltration, the observed *in situ* immune response is unlikely to be tumour specific.

### *Ras* oncogene expression in colorectal cancer

D.J. Jones[1,2], N. Salhab[1,2], A. Kinsella[1], P.F. Schofield[2] & M. Moore[1]

[1]*Paterson Institute for Cancer Research and* [2]*Department of Surgery, Christie Hospital, Manchester M20 9BX, UK.*

c-Ha-*ras* and c-Ki-*ras* gene expression were studied in 31 colorectal carcinomas by Southern blotting and immunohistochemical staining of cryostat sections using the monoclonal antibody, Y13–259. Amplification of Ha-*ras* and Ki-*ras* were detected in 11 and 9 tumours respectively (two had amplification of both genes). Two tumours with low level amplification also had point mutation at codon 12. Staining was membrane associated in normal mucosa, but predominantly cytoplasmic in carcinomas. Staining intensity was unrelated to the degree of amplification. The abnormal distribution in carcinomas may be due to perturbed production and handling of *ras* proteins in malignancy. *Ras* gene abnormalities were independent of differentiation, Dukes' stage and DNA ploidy status (determined by flow cytometry), but, there was a significant association between gene amplification and a previous or family history of colorectal cancer (Fisher; $P=0.013$). These preliminary data suggest gene amplification may identify patients at risk of developing metachronous tumours and population subgroups for whom screening may be appropriate.

### Modulation of colonic cell phenotype by growth on collagen gels

J.M. Walling[1], J.A. Hickman[1] & S. Townsend[2]

[1]*CRC Experimental Chemotherapy Group, Pharmaceutical Sciences Institute, Aston University, Birmingham B4 7ET; and* [2]*MRC Radiobiology Unit, Chilton, Didcot, Oxon OX11 0RD, UK.*

Previous studies on mouse mammary epithelium have shown that growing cells on floating collagen gels induces the expression of genes normally associated with the differentiated phenotype (e.g. Lee *et al.*, *J. Cell Biol.*, **98**, 146, 1984). We have studied the effect of collagen gels supplemented with other extracellular matrix components on the growth of three mouse colon cell lines *in vitro* (MAC 13j, MAC 15j, MAC 26j). These cell lines were newly derived from serially transplanted adenocarcinomas of the large bowel induced by prolonged administration of 1,2 dimethylhydrazine (Double *et al.*, *J. Natl Cancer Inst.*, **54**, 271, 1975). Morphologically, the MAC 15j cell line has the most differentiated phenotype *in vitro*. All three cell lines are heterogeneous populations consisting of both goblet cells and non goblet, possibly enterocytic cells.

The growth rate of the MAC 13j cell line was independent of substrate (plastic *vs.* type 1 collagen gel) whereas growth of MAC 15j cells was retarded by a collagen substrate. The major phenotypic effect in these cells when cultured on collagen was the almost complete loss of goblet cells. Whether this represents true terminal differentiation of committed stem cells or simple selection for the enterocyte type cell remains to be determined. Electron microscopic examination revealed little change in ultrastructure when cells were grown on type 1 collagen supplemented with fibronectin, type IV collagen or laminin.

### Laminin inhibits the attachment of glioma cells to type IV collagen

G. Hunt* & G.V. Sherbet

*Cancer Research Unit, University of Newcastle upon Tyne, Newcastle upon Tyne NE1 7RU, UK.*

Laminin, a basement membrane glycoprotein, is known to act as an attachment factor for some cell types and thus has been implicated in the metastatic process. This study has investigated the effect of laminin on the attachment of [methyl-$^3$H] thymidine-labelled cells to tissue culture wells coated with type IV (basement membrane) collagen. The attachment of the B16 murine melanoma BL6 metastatic variant was enhanced ($P<0.01$ by two sample *t* test), the attachment of the B16F1 poorly metastatic variant was unaffected and the attachment of human glioma cell lines G-IJK, G-UVW and U373MG and rat glioma cell line $C_6$ was inhibited ($\beta<0.01$, $\beta<0.01$, $\beta<0.05$ and $\beta<0.001$ respectively, by two sample *t* test). Laminin appeared to exert its effect by adsorption to the collagen and was not cytotoxic to the glioma cells. One of the most unusual features of human and experimental gliomas is the apparent rarity of spontaneous metastasis to extracranial sites. However, the effect of laminin observed in this study may not be the only factor determining the metastatic inefficiency of this tumour type.

*Present address: Department of Pathology, University of Newcastle upon Tyne, UK.

### The role of protein kinase C (PKC)) in the growth inhibition caused by 12,0-tetradecanoylphorbol-13-acetate (TPA) and related compounds

I.L. Dale & A. Gescher

*CRC Experimental Chemotherapy Group, Pharmaceutical Sciences Institute, Aston University, Birmingham B4 7ET, UK.*

TPA potently inhibits the growth of A549 human lung carcinoma cells with an $IC_{50}$ of 0.1 nM (Gescher & Reed, *Cancer Res.*, **45**, 4315, 1985). Mezerein, a tumour promoter which is structurally related to TPA, induces less potently the same growth-inhibitory effects as TPA ($IC_{50}$ 4 nM) (Dale & Gescher, *Br. J. Cancer.*, **56**, 184, 1986). In the present study the potential role of PKC in the TPA-induced growth arrest of A549 cells has been further investigated. It was found that the synthetic diacylglycerols 1-oleoyl-2-acetylglycerol and 1,2-dioctanoylglycerol were unable to induce growth arrest at non-toxic concentrations (<100 μM). Also, the specific PKC inhibitor H-7 at concentrations of up to 100 μM did not alter growth inhibition induced by 10 nM TPA. PKC activity and its subcellular distribution were determined after partial purification by non-denaturing PAGE. In cultures treated with TPA gross PKC translocation occurred from the cytosol to the membrane within 30 min. Translocation was half-maximal on incubation with 50 nM TPA. On further incubation PKC activity was completely downregulated within hours. Diacylglycerols did not induce PKC translocation. PKC translocation caused by 100 nM mezerein was only 25% of that observed with 100 nM TPA. These results suggest that (i) activation of PKC is insufficient for growth arrest in A549 cells; and (ii) growth inhibition occurs at concentrations much lower than that required for gross enzyme translocation.

### Concentration-effect modelling for cytotoxic drugs

D.J. Kerr & S.B. Kaye

*CRC Department of Medical Oncology, University of Glasgow, UK.*

There are few data relating the activity of cytotoxic drugs *in vitro* to their antitumour efficacy *in vivo*. Usually an arbitrary criterion of effect *in vitro* e.g. the concentration of drug which induced 50% clonogenic cell kill ($ID_{50}$) *in vivo*, such as the peak plasma concentration of the drug at the host $LD_{10}$ dose (i.e. dose which kills 10% of animals). This allows very limited correlation between model systems of different complexity and is impractical for predictive ranking of drug activity. Following detailed pharmacokinetic studies conducted in nude mice bearing xenografts we have constructed a computer model using non-linear least squares fitting which relates plasma concentration of doxorubicin to tumour concentration. We used published data to provide clonogenic cell survival data for MGH–UI cells grown *in vitro* (monolayer and spheroid) and *in vivo* as xenografts. Log dose response curves for monolayer and spheroid data was fitted to a Hill-type equation and by combining these parameters with the pharmacokinetic ones it was possible to predict clonogenic survival in xenografts from the *in vitro* data.

| *Surviving clonogenic fraction (xenograft) (post treatment)* | *Actual* | *Predicted* | |
|---|---|---|---|
| | | *Monolayer* | *Spheroid* |
| 2 h | 0.83 | 0.74 | 0.9 |
| 18 h | 0.76 | 0.37 | 0.65 |

Clearly the spheroid model is a better predictor of response *in vivo* than monolayer and this relates to its three-dimensional configuration and relative drug resistance.

### Development and characterisation of cisplatin-resistant human bladder and testicular tumour cell lines

M.C. Walker & J.R.W. Masters

*Institute of Urology, St Paul's Hospital, London WC2, UK.*

Cisplatin (CP) is the most active single agent in the treatment of advanced testicular germ cell tumours and transitional cell carcinomas of the bladder. Consequently, resistance to this drug is an important cause of treatment failure.

CP-resistant cells were derived *in vitro* from a human testicular tumour cell line (SuSa) and a bladder carcinoma cell line (RT112) by continuous exposure to increasing concentrations of CP. SuSa cells were initially exposed to 50 ng ml$^{-1}$ CP, and after 12 months were able to grow continuously in 300 ng ml$^{-1}$ CP. RT112 cells were initially exposed to 80 ng ml$^{-1}$ CP and after 15 months were able to grow continuously in 3.5 $\mu$g ml$^{-1}$ CP. Comparing IC70s (CP concentration required to kill 70% of clonogenic cells) (see Table), SuSa-CP was 6-fold more resistant than SuSa, and RT112-CP was 4-fold more resistant than RT112. These levels of resistance were retained when the sublines were maintained in the absence of CP for 3 months.

The isozyme profiles, population doubling times (PDT) and plating efficiencies (PE) of parent and resistant cell lines were studied (see Table). The isozyme studies showed that the resistant sublines were not cross-contaminated with other cell lines.

| *Cell line* | *ID$^{70}$ ($\mu$g ml$^{-1}$) 1 h exposure* | *PDT (h)* | *PE (%)* |
|---|---|---|---|
| RT112 | 5 | 22 | 71 |
| RT112-CP | 20 | 22 | 51 |
| SuSa | 0.8 | 31 | 7 |
| SuSa-CP | 5 | 32 | 17 |

### Spectrum of differential drug sensitivities *in vitro* of a testicular germ cell tumour

C. Xiao[1,2] & J.R.W. Masters[1]

[1]*Urological Oncology Unit, University College, St Paul's Hospital, London WC2H 9AE; and* [2]*Department of Urology, Tongji Medical University, Wuhan, PR China.*

Testicular germ cell tumours are curable in the majority of cases using combination chemotherapy, even at advanced clinical stages of disease. We have shown that continuous cell lines derived from these tumours are significantly more sensitive to drugs, including cyclophosphamide, cisplatin and adriamycin (M.C. Walker *et al.*, *J. Natl Cancer Inst.*, **79**, 213, 1987) and gamma-irradiation than bladder cancer cell lines. In order to identify the mechanism underlying the differential sensitivity of testicular tumour cells we have now compared the sensitivities of a continuous cell line derived from a non-seminomatous testicular germ cell tumour, 833K, with those of a cell line derived from a transitional cell carcinoma of the bladder, RT112, to a spectrum of anti-cancer agents of different classes and mechanisms of action. These included the alkylating agents chlorambucil and thiotepa, the antimetabolites methotrexate and 5-fluorouracil, the antibiotics bleomycin, mitomycin-c and daunorubicin, the nitrosoureas CCNU and BCNU, the topoisomerase inhibitors m-AMSA and VP16, and vincristine and vinblastine.

Preliminary data indicate that the testicular tumour cells are more sensitive than the bladder tumour cells to all the agents tested. Because the spectrum of sensitivity to drugs is so broad, a possible explanation for the differential sensitivity of testicular tumours is a single common deficit in a fundamental aspect of DNA repair.

### Chemosensitivity to doxorubicin of early passage cells from breast biopsies

S. Stallard[1], R.I. Freshney[1], J.A. Bradley[2] & W.D. George[2]

[1]*Department of Medical Oncology and* [2]*Department of Surgery, University of Glasgow, UK.*

Little is known about the relative sensitivities of breast carcinomas and normal breast tissue, although it is often assumed that tumours are the more sensitive. In this study, a colony forming assay has been used to examine the relative sensitivities of breast tumour cells and normal breast epithelial cells to doxorubicin. Paired samples of tumour and of normal breast were obtained from eleven previously untreated women undergoing mastectomy. After collagenase digestion organoids were plated onto plastic, and epithelial cell outgrowths were maintained in primary culture, where they grew as ring colonies. The epithelial nature of these cells was confirmed by morphology, by EM, and by staining with antibodies to cytokeratin, lactalbumin and HMFG 1 and 2.

After 5 to 7 days proliferating cells were subcultured onto fibroblast feeder layers and were treated *in situ* with doxorubicin at a range of concentrations for 24 h. Colonies were then counted after two weeks. The drug concentration required to kill 50% of the tumour cells varied about twenty-fold (range $2.7 \times 10^{-8}$ to $6 \times 10^{-7}$ M). In all eleven patients, the dose response curves were similar for tumour and for normal breast.

These findings suggest that there is marked inter-patient variation in doxorubicin sensitivity, but that breast tumour sensitivity is close to that of normal tissue in a single individual.

### *In vitro* and *in vivo* responses of a panel of murine colon tumours to adriamycin: A poor correlation

R.M. Phillips, M.C. Bibby & J.A. Double

*Clinical Oncology Unit, University of Bradford, Bradford, West Yorkshire, BD7 1DP, UK.*

The present study was undertaken to assess whether or not a tumour colony forming assay could have retrospectively predicted the response of a panel of murine colon tumours (MAC tumours) to adriamycin (ADR). Previous studies in this laboratory have described the response of two subcutaneously grown solid tumours, MAC 13 and 30T to ADR (Double & Ball, *Cancer Chemother. Rep.*, **59**, 1083, 1975; Double & Bibby, *Br. J. Cancer*, **48**, 739, 1983). Both tumours are resistant to a single bolus injection of ADR (10 mg Kg$^{-1}$ i.p.). In this study, MAC 15A tumours grown either as an ascites or systemically as small 'spheroid like' lung nodules demonstrated only partial sensitivity to ADR (10 mg kg$^{-1}$, i.p. and i.v.). The inherent chemosensitivity of cell lines derived from these tumours was assessed using a modified clonogenic assay (Bibby *et al.*, *Br. J. Cancer*, **55**, 1987) at therapeutically relevant drug exposures. Increasing the duration of drug exposure resulted in increased cytotoxicity for all the time points studied suggesting that improved anti-tumour effects *in vivo* may be achieved if ADR is administered by continuous infusion or by 'split scheduling'. In all the cell lines tested, significant reductions in colony formation (>90%) were observed *in vitro* under time and concentration conditions that approximate plasma and peritoneal $C \times t$ values for ADR in non-tumour bearing NMRI mice following i.p. and i.v. bolus injections of ADR ($10\,mg\,kg^{-1}$). On the basis of these findings, good responses *in vivo* would be predicted. The poor correlation that exists between *in vivo* responses to ADR and *in vitro* chemosensitivity as assessed by a clonogenic assay, suggest that other factors, possibly related to the three-dimensional structure of solid tumours, have a significant influence on the final outcome of chemotherapy in this model.

### Characterization of a tetrazolium based chemosensitivity assay suitable for non-adherent small-cell lung cancer cell lines

J.A. Plumb, R. Milroy & S.B. Kaye

*Department of Medical Oncology, University of Glasgow, Glasgow G12 9LX, UK.*

Development of drug resistance is common in small-cell lung cancer. However, studies of drug resistance *in vitro* with small-cell lung cancer (SCLC) cell lines present major problems. The cell lines are frequently non-adherent and grow as floating aggregates which do not disrupt into viable single cell suspensions. Hence standard chemosensitivity assays are not suitable. Those used often rely on disruption of the aggregate and hence a decrease in cell viability prior to drug exposure. We have characterised and modified a chemosensitivity assay based on the reduction of a tetrazolium dye, MTT, by live but not dead cells. For adherent cells this assay correlates well with a standard clonogenic assay, provided that sufficient MTT is used and the pH is controlled. We have now validated this assay for use with non-adherent small-cell lung cancer cell lines. Frozen sections of SCLC cell aggregates incubated in MTT contain MTT-formazan crystals throughout the viable cell population. Furthermore, we have also shown by fluorescent microscopy of frozen sections that adriamycin is able to penetrate throughout this same cell population. This contrasts with our observations of limited penetration of adriamycin in spheroids prepared from mono-layer cell lines. Thus, all cells in the SCLC aggregates are exposed to drug and MTT reduction can be used as an estimate of total cell numbers without the need to disrupt the aggregate. We have used MTT-formazan production to measure both the growth rates and chemosensitivities of a number of newly established cell lines.

### The MTT assay underestimates growth inhibition by interferons in human lung cancer cells

S.A.B. Rashid, P.R. Twentyman & J.V. Watson

*MRC Clinical Oncology and Radiotherapeutics Unit, Hills Road, Cambridge CB2 2QH, UK.*

The growth inhibitory effects of recombinant interferons (IFNs) $\alpha$ and $\gamma$ on human lung cancer cell lines was studied using both a tetrazolium (MTT) colorimetric assay and direct cell counting. Significant discrepancies between the two assays were observed (see table below).

| *Cell lines* | | *% Inhibition of cell growth of controls* | | | |
|---|---|---|---|---|---|
| | | *$\alpha$ IFN at 1 $kU\,ml^{-1}$* | | *$\gamma$ IFN at 1 $kU\,ml^{-1}$* | |
| | | *MTT* | *Cell count* | *MTT* | *Cell count* |
| 'Classic' small cell | NCI-H69 | 10–20 | 20–40 | 10–15 | 20–30 |
| | POC | 10–20 | 50–60 | 20 | 60–80 |
| | COR-L88 | 30 | 70–80 | 10–15 | 10 |
| Large cell | COR-L23 | 10–20 | 20 | 30 | 75–85 |

There is no direct chemical effect of IFNs on the tetrazolium reduction process. IFN treated cells showed increased cell size (Coulter) compared with control cells, although there was little or no change in cell cycle distribution. Mitochondrial activity (the basis of formazan production) was 30% greater in $\gamma$ IFN treated cells (COR-L23) than the controls. Reduced formazan production/cell was observed in medium which had supported cell growth for several days. Differential 'medium conditioning' led to a difference in formazan production/cell between IFN and control cells and this was the major basis of the observed discrepancy.

### Small cell lung cancer: Alteration of tumour cell phenotype *in vitro* and its effect on chemosensitivity

A.M.B. Murray & R.I. Freshney

*Department of Medical Oncology, University of Glasgow, Glasgow, UK.*

Elevated levels of L-DOPA decarboxylase (DDC) and creatine kinase BB (CKBB) have been shown to be characteristic of a number of small cell lung cancer (SCLC) cell lines. These two properties have been selected to represent the SCLC phenotype. The aim of this study was to determine whether the SCLC phenotype can be altered by drugs known to induce phenotypic changes in other cell lines.

Cells in log phase were exposed to drug for 72 h, harvested and their DDC and CKBB activities determined. DDC activity was determined from the rate of formation of $^3$H-dopamine from $^3$H-DOPA, and CKBB activity by a NAD linked enzymatic method and characterised by electrophoretic analysis.

HMBA (1.0 mM) reduced the DDC activity in both NCI-H187 ($P<0.05$) and NCI-H69 ($P<0.01$), and increased the CKBB activity in H69 ($P<0.05$). Moreover, sodium butyrate (0.1 mM) reduced the DDC levels in the three lines examined, H187 ($P<0.01$), H69 ($P<0.001$) and NCI-H128d ($P<0.02$), and increased the CKBB levels in H187 ($P<0.01$) and H69 ($P<0.05$). Similarly, dibutyryl cAMP (0.5 mM) was also found to reduce the DDC activity in H69 ($P<0.05$), and increase the CKBB levels ($P<0.05$). All drug concentrations used were non-cytotoxic.

These results suggest that the SCLC phenotype can be altered by drug treatment. We have investigated whether these phenotypic changes affect the chemosensitivity of the cells. A chemosensitivity assay was used based on the reduction of a tetrazolium dye. The adriamycin ID50 was found to be $3.51 \times 10^{-8}$ M for H187, $4.37 \times 10^{-8}$ M for H69 and $7.5 \times 10^{-8}$ M for H128d. However, the effect of the above drug induced changes on the chemosensitivity of the cell lines is as yet unclear.

### Actions of a progestogen on the efficacy of cytotoxic drugs

N.A. Shaikh[1], K. Patel[2], A.M. Owen[2], M.W. Ghilchik[1] & H. Braunsberg[2]

[1]*Breast Clinic and* [2]*Chemical Pathology Department, St Mary's Hospital and Medical School, London W2 1PG, UK.*

The use of hormones to enhance the effects of cytotoxic drugs has met with partial success, but exploration of this approach has not yet been exhausted. We have achieved very promising clinical results by sequential administration of oestradiol ($E_2$), medroxyprogesterone acetate (MPA) and cytotoxic drugs and have now attempted to validate this therapy by *in vitro* experiments with cultured human breast cancer cells (MCF-7).

Cells, grown in the presence of dextran-charcoal stripped foetal calf serum for 3 days, were treated with or without MPA (48 h) followed by drug (or control) (24 h) in the same medium. The washed monolayers were grown for a further 3 days with medium containing untreated FCS and cell yields estimated. Hormone-drug interactions were assessed by Student's *t* tests, analysis of variance and estimation of apparent doubling times.

MPA (10–80 nM) significantly affected the efficacy of methotrexate (MTX) and vincristine (VCR) at concentrations (10 and 0.1 nM, respectively) at which the drugs alone gave a barely detectable decrease in cell yields. The *in vitro* effects of sequential oestradiol/MPA treatment and those of MPA on the action of other drugs used in our regimen are under investigation.

The enhancement of the action of cytotoxic drugs through pretreatment with MPA, now also demonstrated *in vitro*, deserves more extensive clinical trials.

**Survival studies in Chinese hamster ovary cells following drug or radiation exposure using the cytochalasin assay in comparison to a clonogenic method**

J.B. Court, C. Burn & J.L. Moore

*Radiation Sciences Laboratory, Velindre Hospital, Cardiff CF4 7XL, Wales, UK.*

We have developed (Court & Moore, *Cell. Biol. Int. Rep.*, **9**, 219, 1985) an assay for Chinese hamster ovary cell viability that is based on the ability to multinucleate in the presence of the fungal metabolite cytochalasin B (CB) following exposure to radiation or to cytotoxic drugs. The degree of multinucleation achieved is assessed after a given incubation period in CB by measurement of the cellular DNA content using the flow cytometer. The method is intended to permit estimation of the survival of DNA synthesis in situations where cells cannot be fully monodispersed.

Cells were seeded from the same stock culture at either 400 cell/flask for conventional clonogenicity studies, or at 1 x 10E6 cell/flask for the estimation of the DNA content following incubation in CB. All cultures were exposed to the same cytotoxic drug solution, or to 137-Cs gamma irradiation either at a high dose-rate of 0.5 Gy min$^{-1}$, or at a low dose-rate of about 1 cGy min$^{-1}$.

Radiation survival curves after single doses up to 20 Gy are exponential, with no evidence for a threshold in the survival of the ability to reach a DNA content of at least 3 x G1 in the presence of CB. We have investigated the relationship between this apparent lack of repair of sublethal damage in the multinucleation system to the change in survival curve slope at low dose rates. Comparisons have also been made between the survival of clonogenic potential and the survival of DNA synthesis, in CHO cells exposed to cisplatinum, Novantrone, cytosine arabinoside, 5-fluorouracil, or vinblastine, all as single agents.

**Cytotoxicity of fatty acids on normal and malignant cells *in vitro***

B. Fermor[1], N.A. Habib[1], J.R.W. Masters[2], J.C. Coffey[2], C.B. Wood[3] & R.C.N. Williamson[3]

[1]*Department of Surgery, Bristol Royal Infirmary;* [2]*Institute of Urology, London;* [3]*Department of Surgery, RPMS, London, UK.*

The membranes of malignant cells showed increased oleic acid in relation to stearic acid content in comparison with their normal counterparts (Wood *et al.*, *Eur. J. Onc.*, **11**, 347, 1985). The cytotoxic effects of saturated stearic, monounsaturated oleic and dihydroxystearic acids on two testicular tumour (SuSa, 833K), two bladder carcinoma (RT112, RT4), one colorectal tumour (HT29), one normal urothelium (HU609) and one normal fibroblast (HFL) cell lines were measured using a colony forming assay. Stearic acid was up to five times more cytotoxic than oleic acid.

| | | *IC 50 μg m$^{-1}$l (mean ± s.e.m.)* | |
|---|---|---|---|
| *Tumour* | *Cell line* | *Stearic acid* | *Oleic acid* |
| Testis | SuSa | 2.8 ± 1.4 | 16.5 ± 2.3 |
| | 833K | 5.0 ± 0.2 | 26.6 ± 2.5 |
| Bladder | RT112 | 4.4 ± 0.8 | 22.4 ± 3.0 |
| | RT4 | 7.6 ± 0.4 | 25.0 ± 0.7 |
| Colon | HT29 | 7.6 ± 0.2 | 34.3 ± 0.8 |
| Fibroblasts | HFL | 8.7 ± 0.1 | 21.8 ± 0.7 |

All tumour cell lines with the exception of RT4 were more sensitive to stearic acid than the normal fibroblasts ($<0.001<P<0.05$). However the well differentiated bladder tumour, RT4 was more sensitive than normal fibroblasts to oleic acids ($P<0.05$). It was also found that the 3 tumour cell lines tested were more sensitive than the normal urothelium and fibroblasts to dihydroxystearic acid (data not shown). Our data suggest that the stearic acid is more cytotoxic to tumour cells than oleic acid, and that this may be related to the higher proportion of oleic acid in the tumour cell membranes.

**Thermochemotherapy with melphalan and human melanoma spheroids**

R.N. Scott, T.E. Wheldon, S.B. Kaye, R.M. MacKie & A.J. McKay

*Departments of Surgery and Oncology, Gartnavel General Hospital; Radiobiology Group, Belvidere Hospital; and Department of Dermatology, Western Infirmary, Glasgow, UK.*

Isolated limb perfusion (ILP) is the most effective treatment for loco-regional recurrence of melanoma. The value of hyperthermia during ILP with melphalan is controversial.

Both ILP and the multicellular tumour spheroid (MTS) model allow the manipulation of tumour microenvironments outwith normal physio-pharmacological ranges. The MTS model is thus a useful analogy for conditions during ILP.

The aim of this work was to study the effect of temperature on the cytotoxic effect of melphalan using human melanoma MTS.

We have grown MTS from several established cell lines. MTS of the B8 line were exposed to clinically achieved concentrations of melphalan for 1 h at temperatures from 31 to 44.5°C. Spheroid volume was measured subsequently to obtain growth curves. Spheroids were also disaggregated for clonogenic assay.

Using regrowth delay and clonogenicity of disaggregated spheroids our results show that, despite the increased rate of hydrolysis of melphalan at higher temperatures, the cytotoxic effect of the drug is enhanced by heat. At hypothermic temperatures there was no significant difference in cytotoxic effect compared with normothermia.

These results support the use of hyperthermia during ILP with melphalan.

**Mutation and drug resistance in human tumour cell lines *in vitro***

C.N. Parris & J.R.W. Masters

*Institute of Urology, St Paul's Hospital, London WC2H 9AE, UK.*

Testicular germ cell tumours are curable in advanced stages using chemotherapy, unlike most other types of cancer. Drug resistance in all forms of cancer may develop as a result of mutation induced by chemotherapy. The spontaneous and induced mutation frequencies (MF) in cell lines derived from three testicular (SuSa, 833K, GH) and two bladder (RT112, RT4) tumours were compared. In addition MF were compared also in a testicular and bladder subline made resistant to the drug cisplatin (SuSa-CP, RT112-CP). Hypoxanthine guanine phosphoribosyl transferase (HGPRT) locus mutations were selected by resistance to 10 $\mu g\,ml^{-1}$ 6 thioguanine. Induced mutation frequencies were compared following exposure to a range of concentrations of the mutagen ethyl methane sulphonate (EMS) (see Table).

| *Cell type* | *Line* | *Spontaneous MF* | *MF at 0.6 mg ml$^{-1}$ EMS* |
|---|---|---|---|
| Bladder | RT112 | $2.5\times10^{-5}$ | $3.1\times10^{-5}$ |
| Bladder | RT112-CP | $1.8\times10^{-5}$ | $2.2\times10^{-5}$ |
| Bladder | RT4 | $4.4\times10^{-6}$ | $5.6\times10^{-6}$ |
| Testis | SuSa | $6.9\times10^{-6}$ | $1.1\times10^{-5}$ |
| Testis | SuSa-CP | $5.2\times10^{-6}$ | $2.2\times10^{-5}$ |
| Testis | 833K | $5.8\times10^{-6}$ | $2.2\times10^{-5}$ |
| Testis | GH | $1.5\times10^{-6}$ | $8.0\times10^{-6}$ |

Within the bladder and testicular cell lines there is overlap of both spontaneous and induced MF. In the cell lines made resistant to cisplatin there is no significant change in MF. In these cell lines there is no association between drug sensitivity or resistance and mutation frequencies.

**Elevation of HL-60 cell intracellular calcium by the cytotoxic ether lipid SRI 62-834 and antagonism by 12-0-tetradecanoyl-phorbol-13-acetate**

M.G. Thompson & J.A. Hickman

*CRC Experimental Chemotherapy Group, Pharmaceutical Sciences Institute, Aston University, Birmingham B4 7ET, UK.*

SRI 62-834 (($\pm$) - 2 - [hydroxy[tetrahydro - 2 - (octadecycloxy)methylfuran - 2 - yl] - methoxy]phosphinyloxy - N, N, N - trimethylethaniminium hydroxide) is a tetrahydrofuran analog of the antitumour ether lysophospholipid Et-18-$OCH_3$(1 - 0 - octadecyl - 2 - 0 - methyl - rac - glycero - 3 - phosphocholine). The cytotoxic and differentiation-promoting activity of the ether phospholipids, such as Et-18-$OCH_3$, is considered to be mediated either via direct effects, and/or indirectly by the activation of cytotoxic macrophages. The direct cytotoxicity has been suggested to be the result of the inhibition of normal phospholipid metabolism and the subsequent perturbation of membrane function. In view of this, we have investigated the activity of SRI 62-834 on the calcium homeostasis of HL-60 human promyelocytic leukaemia cells, and find it rapidly to elevate intracellular calcium concentrations in a concentration-dependent manner, as monitored by the calcium indicator, Quin-2. In a typical experiment, 30 $\mu$M SRI 62-834 elevated the concentration of intracellular calcium from a resting level of 179 nM to 445 nM/1.5 x 10 cells. This was partially inhibited by preincubation with the voltage-dependent calcium-channel blocker, verapamil, and completely prevented by the protein kinase C-activating phorbol ester, 12-0-tetra - decanoyl - phorbol - 13 - acetate (TPA). After a 10 min preincubation with 3 nM TPA, the calcium rise induced by 30 $\mu$M SRI 62-834 was 50% inhibited, and 100 nM TPA completely abolished it. This suggests that the elevation of intracellular calcium mediated by SRI 62-834 is possibly the result of the opening of a calcium channel, the activity of which is regulated by protein kinase C.

***In vivo*-induced resistance to chloroethylating agents correlates with increased $O_6$-alkylguanine DNA alkyltransferase expression in a murine solid tumour**

P. Workman[1], K.L. Kooistra[1], F.Y.F. Lee[1], J. Donaldson[1] & G.P. Margison[2]

[1]*MRC Clinical Oncology Unit, Cambridge; and*
[2]*Department of Carcinogenesis, Paterson Institute, Manchester, UK.*

Sensitivity of mammalian cell lines to chloroethylating agents is associated with low expression of the repair protein $O^6$-alkylguanine DNA alkyl(transferase (ATase). This removes the chloroethyl monoadduct DNA from the $O^6$ position of guanine, thus preventing potentially lethal DNA crosslink formation. However, it is still not clear at this time to what extent *de novo* and acquired resistance to chloroethylating agents are associated with increased expression of ATase in solid tumours. The KHT sarcoma of C3H mice is highly sensitive to chloroethylating agents. By serial *in vivo* treatment with CCNU or mitozolomide and retransplantation, we produced two sublines (KHT/CCNU and KHT/mitozol) with stable resistance to these agents: e.g., CCNU, BCNU, mitozolomide and clomesome produce growth delays of 0–4 days, compared to 20–30 days with some cures for the parent tumour. ATase activity was assayed by the transfer of radioactivity from $^3$H-methylnitrosourea-methylated calf thymus DNA. The levels ($\pm$2 s.e.) in fmol/mg protein were: parent KHT 19.0$\pm$6.0; KHT/CCNU 53.2$\pm$12.6; KHT/mitozol 73.7$\pm$5.3 (both $P<0.001$). We conclude that *in vivo*-induced resistance of the KHT tumour to chloroethylating agents is associated with a 3–4 fold increase in ATase activity, thus supporting a role for up-regulation of ATase gene expression as a molecular mechanism of resistance.

**Changes in cellular glutathione and glutathione-S-transferase levels associated with resistance to L-phenylalanine mustard (melphalan)**

R.A. Britten & J.A. Green

*CRC Department of Radiation Oncology, University of Liverpool, Clatterbridge Hospital, Bebington, Wirral L63 4JY, UK.*

Cellular glutathione plays an important role in the modulation of alkylating agent cytotoxicity. The relationship between cellular glutathione transferase (GSH-TFR), GSH and GSSG levels and alkylating agent resistance has been studied in the human ovarian OAW42 cell line. The effect of oxothiazolidine-4-carboxylate (OTZ) and buthionine sulfoximine (BSO) has also been determined. The melphalan-resistant subline OAW42/MER was developed by step-wise incubation with the drug, and found to be 4-fold more resistant to melphalan than the parent OAW42 line. Total glutathione and GSH-TFR activity were 1.5 fold greater in OAW42/MER than in OAW42. The GSSG/GSH-TFR ratio was 0.11 in OAW42, and 0.25 in OAW42/MER. The difference in glutathione levels was therefore attributed to an elevation in GSSG content of OAW42/MER. 50 $\mu$M BSO (18 h) potentiated melphalan cytotoxicity by a factor of 2 in both cell lines, and reduced total glutathione to 78.6% and 40.3% of control values respectively in OAW42 and OAW42/MER. The level of GSSG was unchanged in OAW42 but fell to 8.6% in OAW42/MER. GSH-TFR activity decreased to 64% of control levels in both cell lines. The GSSG/GSH-TFR ratio remained at 0.11 in OAW42 cells, but fell to 0.08 in OAW42/MER.

Exposure to 50 $\mu$M OTZ for 18 h reduced melphalan cytotoxicity by 5-fold, and significantly increased the GSSG/GSH-TFR ratio in OAW42 to 0.20. Total glutathione levels were not altered, but the GSSG/GSH ratio increased by 2 fold. These results suggest a correlation between the proportion of total glutathione in the oxidized form GSSG, GSH-TFR activity and the degree of resistance to melphalan.

### Drug sensitivity in tumour cell lines with different glutathione transferase content: Dramatic over expression of enzyme levels in cell lines resistant to 1-chloro-2,4-dinitrobenzene

C.J. Wareing[1,2], A.D. Lewis[2], J.D. Hayes[1] & C.R. Wolf[2]

[1]*Department of Clinical Chemistry, Royal Infirmary, Edinburgh EH3; and* [2]*ICRF Laboratory of Molecular Pharmacology and Drug Metabolism, Hugh Robson Building, George Square, Edinburgh EH8 9XD, UK.*

The glutathione-S-transferases (GSTs) are a multigene family of enzymes which play a role in many cellular detoxification reactions. Evidence is emerging that these enzymes also play a role in the development of resistance to cytotoxic drugs and anti-neoplastic agents. We are studying the relationship between GST expression in human tumour cell lines and their susceptibility to these compounds. The MCF7 cell line which contains essentially no GST was much more sensitive than the cell line HT29 to the toxic effects of GST substrates and chlorambucil. In an alternative approach we have made a human lung tumour cell line (NCI H322) resistant to the GST substrate CDNB. The resistant line demonstrates dramatic over expression of GST activity (24 fold), to levels higher than any normal human tissue, including the liver. Elevation in both the acidic and basic subunits accompanied this change. The basic subunit being elevated greater than 100 fold in the resistant line.

### Pre-treatment of cells with verapamil enhances the effects of verapamil on chemosensitivity *in vitro*

J. Plumb & S.B. Kaye

*Department of Medical Oncology, University of Glasgow, Glasgow G12 9LX, UK.*

The calcium antagonist verapamil has been shown to increase the sensitivity of a number of cell lines to cytotoxic drugs *in vitro* and it has been suggested that it may be of value in attempts to overcome drug resistance in patients. Although verapamil (6.6 $\mu$M) increased the sensitivity of a number of non-small-cell lung cancer (NSCLC) cell lines to adriamycin, this effect was not observed if verapamil was added to the cells subsequent to addition of adriamycin. This suggested that verapamil is not active if the cells have already been exposed to the cytotoxic drug. We have, therefore, determined the effects of pre-treatment of cells with verapamil prior to exposure to adriamycin.

Chemosensitivity was determined by an assay based on the reduction of a tetrazolium dye, MTT, by live but not dead cells. Cells were plated in 96 well plates and exposed to adriamycin for 24 h. For the NSCLC cell line WIL the $ID_{50}$ for adriamycin alone was $3.0 \times 10^{-8}$ M. This was reduced 5-fold by exposure to adriamycin for 24 h in the presence of verapamil (6.6 $\mu$M). However, when cells were incubated for 24 h in the presence of verapamil (6.6 $\mu$M) prior to addition of adriamycin and verapamil, the $ID_{50}$ was reduced by 20-fold. In contrast addition of verapamil for 24 h after exposure to adriamycin and verapamil did not further reduce the sensitivity of the cell line. Furthermore, pre-incubation with verapamil for 24 h prior to exposure to adriamycin alone had no effect on the chemosensitivity. Although the mechanism of action of verapamil in this context is not known the results suggest that it involves effects on the cell both before and during drug exposure.

### Resistance modifiers verapamil and cyclosporin A: Differential effects on the chemosensitivity and cellular pharmacology of anthracyclines in multidrug resistant cells

H.M. Coley, P.R. Twentyman & P. Workman

*MRC Clinical Oncology and Radiotherapeutics Unit, Hills Road, Cambridge, UK.*

Verapamil (VRP) and cyclosporin A (CYA) are under evaluation for their ability to overcome multidrug resistance. Using H69 human small cell lung cancer cells, EMT6 mouse mammary tumour cells and their adriamycin (ADM) resistant counterparts (*in vitro* derived) we examined the effects of these agents on chemosensitivity and drug uptake. Chemosensitivity testing involved a tetrazolium (MTT) reduction assay with continuous drug exposure. From a range of anthracycline analogues tested we selected three drugs, aclacinomycin A (ACL), Ro 31-1215, both 9-alkyl derivatives, and 4′-deoxy-4′-iodo doxorubicin (4′-Jodo), a 4′-daunosamine derivative, on the basis of their minimal cross-resistance in the ADM resistant cell lines. Resistance factors (RF = $ID_{50}$ resistant line/parent line) for H69 and EMT6 respectively were 225 and 34 for ADM, 5.8 and 4.7 for ACL, 12.4 and 8.1 for Ro 31-1215 and 18.9 and 4.4 for 4′-Jodo. At clinically comparable concentrations CYA (5 $\mu g\,ml^{-1}$) was more effective than VRP (3.3 $\mu g\,ml^{-1}$) in modifying resistance to ADM and all 3 analogues. In EMT6 with ADM, VRP enhanced sensitivity more in the parent line (4–14 fold) than in the resistant line (2–6 fold), whereas the opposite was seen with the 3 analogues. For H69, enhancement was confined to the resistant line. CYA increased chemosensitivity in both resistant lines more than in the parent lines for all 4 agents. Drug accumulation was enhanced in resistant lines to similar extents by VRP and CYA. Levels achieved approached those in the parent lines for ADM, Ro 31-1215 and 4′-Jodo, whereas a negligible effect was seen for ACL. These results indicate the complexity of resistance modification by agents of this type.

### Modification of cytotoxic drug resistance by non-immunosuppressive cyclosporins

P.R. Twentyman[1] & D.J.G. White[2]

[1]*MRC Clinical Oncology and Radiotherapeutics Unit, Hills Road, Cambridge CB2 2QH; and* [2]*Department of Surgery, University of Cambridge, UK.*

We have previously shown (Twentyman *et al.*, *Br. J. Cancer*, **56**, 55, 1987) that the immunosuppressive agent cyclosporin A (CYA) is an effective modifier of resistance to adriamycin (ADM) and vincristine (VCR) in the multidrug resistant variant (LX4) of the human cell lung cancer cell line NCI-H69. Three naturally-occurring analogues of CYA were also examined and we found that their ability as resistance modifiers (RMs) correlated with their immunosuppressive properties.

We have now examined a further series of 6 non- or minimally-immunosuppressive analogues of CYA, either synthetic or derived from naturally-occurring compounds. Several of these have been found to be highly effective RMs. Furthermore, two compounds, B3-243 and W8-032 maintained good activity in the dose-range 1–2 $\mu g\,ml^{-1}$ (sensitisation of LX4 cells to ADM at $2\,\mu g\,ml^{-1} = 53$ and 32 fold respectively), whereas sensitisation to ADM by CYA falls from 50 fold at $5\,\mu g\,ml^{-1}$ to 10 fold at $2\,\mu g\,ml^{-1}$.

Preliminary experiments in mice have indicated that B3-243 can be administered in 5 daily doses of $100\,mg\,kg^{-1}\,day^{-1}$ without acute toxicity. Such a schedule results in blood levels in the range 1–2 $\mu g\,ml^{-1}$, i.e. similar to those which produce effective resistance modification *in vitro*. Experiments combining B3-243 and ADM in mice bearing ADM sensitive and resistant tumours are in progress.

### The effects of soluble factors produced by myeloma cells on bone-derived cells *in vitro*

C. Evans[1], C. Ward & C.S.B. Galasko

*Department of Orthopaedic Surgery, CSB, Hope Hospital, Salford M6 8HD, UK.*

Invasion of bone by tumours usually involves osteolysis as well as some osteogenesis. Multiple myeloma is unusual in that only osteolysis occurs in the vast majority. To examine the effect of myelomata on osteogenesis, the myeloma cell line GM 1500 was co-cultured with human bone-derived cells (BDC). The BDC were also cultured in medium conditioned by the myeloma cells (MCM). BDC proliferation was measured by cell count and DNA synthesis by tritiated thymidine uptake ($^3$H–TdR) assay. Both cell proliferation and DNA synthesis were inhibited by MCM (by 31% and 41% respectively). Proliferation of BDC co-cultured with myeloma cells was also inhibited (26%). This inhibitory effect was dependent upon myeloma cell density in the conditioning medium. Preliminary work showed that this effect is dose dependent, and that the active component has a molecular weight of less than 50,000 daltons. The usual response of bone to injury (including tumour invasion) is osteogenesis. The results of this pilot study suggest that myeloma inhibits this reaction by the secretion of a soluble osteoblast inhibiting factor.

### Substance P antagonists block the mitogenic effects of the neuropeptides bombesin, vasopressin and bradykinin in Swiss 3T3 cells

P.J. Woll & E. Rozengurt

*Growth Regulation Laboratory, Imperial Cancer Research Fund, Lincoln's Inn Fields, London WC2A 3PX, UK.*

The neuropeptides bombesin and vasopressin have previously been shown to be mitogens for Swiss 3T3 cells. We now show that bradykinin is mitogenic for these cells in the presence of insulin. Its effects are dose-dependent and maximal at 10 nM. Addition of bradykinin to the cells causes an immediate and transient increase in the intracellular $Ca^{2+}$ concentration. DNA synthesis commences at 24 h and is maximal by 40 h. All these effects are competitively and reversibly blocked by the substance P antagonists [DArg$^1$, DPro$^2$, DTrp$^{7,9}$, Leu$^{11}$] substance P at 100 $\mu$M and [DArg$^1$, DPhe$^5$, DTrp$^{7,9}$, Leu$^{P1}$] substance P at 20 $\mu$M. These antagonists also selectively antagonise the effects of bombesin and vasopressin in these cells, although the three peptides interact with distinct receptors. We suggest that the substance P antagonist family interacts with a highly conserved common domain of these receptors.

### The rate of passage of cells across the restriction point

D.L. Dewey

*Cancer Research Campaign, Gray Laboratory, Mount Vernon Hospital, Northwood, Middlesex HA6 2RN, UK.*

A large number of genetic markers of events in the mammalian cell cycle are already known. Many are concerned with growth factors and other initiators, but after initiation there is a long gap of many hours before the start of DNA synthesis (S). There is no clear indication of what is happening during this gap except for a point of no return, after which removing all initiating substances will no longer stop cells from completing a round of DNA synthesis. This point in time, usually called the restriction point, is difficult to measure accurately because the cells that continue to enter S after removal of these serum factors do so at a reduced rate. We have found that a low dose of ionizing radiation will not delay cells which have passed the restriction point but will induce a dose related delay in cells which have not reached this point. Moreover, after the delay cells still entered S at the same rate as unirradiated controls. When a culture was irradiated in the middle of the restriction point, half of the cells were delayed and half not. An automatic device for measuring entry into S has been used to plot these double S peaks and by irradiating at frequent intervals it has been possible to measure the rate of passage of a population of cells across the restriction point.

Inhibitors of DNA synthesis act after the restriction point and after removal of the inhibitor, DNA synthesis starts immediately in both irradiated and control cultures. However a latent delay still existed and could be observed in the following cycle.

### $^{31}$P NMR spectral changes in *in vitro* HT29 human colonic adenocarcinoma cells subjected to hypoxia and photon and neutron irradiation

S. Myint & H.M. Warenius

*University of Liverpool Department of Radiation Oncology, Clatterbridge Hospital, Wirral, Merseyside L63 4JY, UK.*

The application of NMR spectroscopy to the study of living cells provides a valuable method for the non-invasive monitoring of cell energetics and ionic content.

We have used $^{31}$P NMR to study metabolic changes related to hypoxia in cultured HT29 human colon carcinoma cells *in vitro* and characterised the $^{31}$P NMR spectrum in monitoring changes occurring during recovery from hypoxia. Phosphate metabolites were monitored with time as the environment in the NMR tube became increasingly hypoxic for the sedimented cell pellet. Considerable changes in the levels of these metabolites were observed. These changes are comparable with those observed *in vivo* tumour with disappearance of phosphocreatine and glycerophosphorylcholine and concomitant increase in inorganic phosphate and are almost completely reversed following reversal of hypoxia by resuspension and reoxygenation in fresh medium. This technique when used to compare cells irradiated by photons or neutrons to controls resulted in different patterns of recovery following irradiation which showed dose response effects. These investigations suggest a potential for NMR spectroscopy as an early *in situ* method of studying the response of tumours to radiotherapy.

**The effects of temperature on post-irradiation recovery in two human tumour cell lines**

J.H. Peacock, A.M. Cassoni, J.J. Eady & T.J. McMillan

*Institute of Cancer Research, Sutton, Surrey, UK.*

The effect of post-irradiation temperature on cellular recovery after exposure to ionizing radiation has been investigated in two human cell lines. One of the lines MGH–U1, showed a reduced shoulder to the radiation dose survival curve when incubated at 25°C for 4 h following treatment, whereas incubation at 0°C for a similar period had no effect. The other line RT112 was unaffected by either post-irradiation temperature.

Split dose experiments showed that recovery from sublethal damage is inhibited during the 4 h at 0°C and 25°C in both lines. When returned to 37°C after these incubations however, RT112 goes on to recover as normal. In contrast, MGH–U1 cells only recover at 37°C when the previous incubation was at 0°C not 25°C.

These results suggest that competing processes of radiation induced damage fixation and repair proceed at 37°C following irradiation and in MGH–U1 cells incubation at 25°C prevents repair but not fixation whereas at 0°C both processes are inhibited.

**Improved fluorescein-based substrates for flow cytometric measurement of cellular esterases can be used in combination with propidium iodide (PI) in a multiparametric assay of cell viability and membrane permeability**

C. Dive, P. Workman & J.V. Watson

*MRC Clinical Oncology Unit, Hills Road, Cambridge CB2 2QH, UK.*

Fluorescein diacetate (FDA) is widely used to measure cell viability, membrane permeability and esterase activity. However, employed as a substrate probe for flow cytoenzymology (FCE) it is limited by rapid efflux of the product fluorescein (F). We have therefore evaluated two potentially improved esterase substrates, carboxyfluorescein diacetate (CFDA) and bis(carboxyethyl) - carboxy - fluorescein - tetra acetoxy methyl ester (BCECF-AM) in terms of reaction and product efflux kinetics. Substrate hydrolysis rates for EMT6 mouse mammary tumour cells were in the order FDA ≪ BCECF-AM > CFDA. The latter had disadvantages of substantial substrate fluorescence, poor cell uptake and complex kinetics, apparently due to participating membrane-bound esterases. In contrast BCECF-AM gave negligible substrate fluorescence and superior membrane permeability. As predicted, product efflux slowed markedly with increased product polarity. Efflux $t_{1/2}$s for the products of FDA and CFDA hydrolysis were 16 and 94 min respectively. No loss of BCEF-AM product (BCECF) was seen after 2 h. Thus BCEF-AM was optimal for FCE. It was subsequently employed in combination with PI in a multiparametric assay of cell viability and membrane permeability. The flow cytometer triggered on light scatter (measuring cell size) and recorded green (BCECF) and red fluorescence (PI). As expected viable cells exhibited green but not red fluorescence while non-viable cells showed the reverse. Interestingly, a third population (~4% of cells) showed little green or red fluorescence. This use of two reporter molecules may provide more detailed information on the relationship between cell viability and membrane permeability.

**Foetal-like fibroblasts in cancer patients and their potential role in disease pathogenesis**

S.L. Schor[1], A.-M. Grey[1], A.M. Schor[2] & G. Rushton[2]

[1]*Department of Cell and Structural Biology, University of Manchester; and* [2]*CRC Department of Medical Oncology, Manchester, UK.*

Fibroblasts from breast cancer patients commonly display certain foetal-like phenotype characteristics. On the basis of these observations, we have suggested that (a) normal foetal-to-adult transitions in fibroblast phenotype do not occur in certain individuals, and (b) the persistence of foetal-like fibroblasts in the adult puts the affected individual at an elevated risk of developing breast cancer by virtue of a dysfunction in normal epithelial-mesenchymal interactions (Schor *et al.*, *Exp. Cell Biol.*, **55**, 11, 1987). We now report that foetal fibroblasts and the foetal-like fibroblasts of cancer patients produce a 55 kD migration stimulating factor (MSF) that is not produced by normal adult fibroblasts, but induces these cells to display a foetal-like migratory phenotype. Foetal fibroblasts undergo a spontaneous transition *in vitro* to expression of a characteristically adult migratory phenotype: this transition is accompanied by a cessation in the production of MSF. In contrast, the foetal-like fibroblasts of breast cancer patients do not undergo this transition and continue to produce MSF until senescense. The primary effect of MSF appears to be a stimulation of glycosaminoglycan (GAG) biosynthesis. We suggest that perturbations in the control of GAG biosynthesis *in vivo* may contribute to tumour progression via their well-documented effects on angiogenesis, cell proliferation, differentiation and migration.

**Expression of cytochrome P-450 cDNAs in yeast**

S. Black[1,2], R.R. Meehan[1,2], J.D. Beggs[3], J.S. Miles[1], M. Adesnik[4] & C.R. Wolf[1]

[1]*ICRF Lab. Mol. Pharmacol., Department of Biochemistry, Edinburgh;* [2]*MRC Clinical and Population Cytogenetics Unit, Edinburgh;* [3]*Dept. Mol. Biol., University of Edinburgh, UK; and* [4]*Dept. Cell Biol. NYU, New York, USA.*

The enzymes encoded by the cytochrome P-450 multi-gene family play a central role in chemical carcinogenesis. However, due to the multiplicity of this enzyme system in any given tissue it is difficult to establish the role of a particular cytochrome P-450 protein in these reactions. The capacity of each individual P-450 form in the metabolism of chemical carcinogens needs to be evaluated.

A potentially powerful approach in such studies is the expression of cytochrome P-450 cDNAs in yeast, an organism which can be used directly for mutation studies. To this end we have isolated a full length cDNA clone for the rat $PB_{3a}$ gene (P450-IIB gene family). In order to express this cDNA in yeast the clone sequence under the control of the *Saccharomyces cerevisiae* (brewers yeast) alcohol dehydrogenase promoter (ADH 1), was incorporated into the 2 micron-type multi-copy yeast shuttle vector pMA56.

The resultant construct has been shown via Western blot analysis to produce $PB_{3a}$ protein at a level of between 0.1%–1% of total yeast cellular protein. The ability of the yeast expressing this P-450 form to metabolize anticancer drugs and various procarcinogens to their active mutagenic forms is currently being evaluated.

**Analysis and preclinical pharmacology of the novel bioreductive alkylating indoloquinone E09**

K.L. Kooistra & P. Workman

*MRC Clinical Oncology and Radiotherapeutics Unit, Hills Road, Cambridge CB2 2QH, UK.*

E09, 3-hydroxymethyl - 5 - aziridinyl - 1 - methyl - 2 - [1H - indole - 4, 7 - dione] - prop - $\beta$ - en - $\alpha$ - ol, is the lead compound in a series of novel indoloquinone compounds synthesized as bioreductive alkylating agents (W.N. Speckamp & E.A. Oostveen, UK and International Patents). As part of our overall interest in molecules undergoing bioreductive activation, we have developed an HPLC assay for E09 and used this to evaluate its preclinical pharmacology in mice. The drug was administered i.v. at $6\,mg\,kg^{-1}$. HPLC analysis was carried out using a Waters Resolve C18 $10\,\mu m$ column with a running buffer of 45% methanol in 20 mM sodium phosphate buffer, pH 7.4. E09 and internal standard, E012, were detected by absorption at 280 nm.

The projected peak plasma levels were $422\,ng\,ml^{-1}$ and the drug was rapidly eliminated with a half-life of 4.2 min. The volume of distribution was large at 355 ml and the clearance $59\,ml\,min^{-1}$. Urine from animals receiving E09 had a bright red appearance, though no parent drug was detected in urine. This pharmacokinetic profile differs greatly from the indoloquinone prototype, mitomycin C. When administered at the same dose, mitomycin C was eliminated with a half-life of 12 min, and exhibited a volume of distribution of 35 ml, clearance of $2\,ml\,min^{-1}$ and peak plasma levels projected at $4.3\,\mu g\,ml^{-1}$. These results show that active concentrations of E09 can be achieved *in vivo*, though it may be appropriate to modulate pharmacological behaviour by using alternative delivery methods or modified analogues.

**Gene amplification as a mechanism of resistance to a novel folate-based thymidylate synthase inhibitor in human W1–L2 cells**

B.M. O'Connor, A.L. Jackman, P.H. Crossley & A.H. Calvert

*Inst. Cancer Res., Sutton, Surrey, UK.*

We reported previously a mutant L1210 cell line with acquired resistance (>200 fold) to the TS inhibitor $N^{10}$-propargyl-5,8-dideazafolic acid (CB3717). TS was overproduced 45-fold and this was shown to be due to amplification of the TS gene. A small (2,5-fold) increase in dihydrofolate reductase (DHFR) was also observed (Jackman *et al.*, *Cancer Res.*, **46,** 2810, 1986). This resistant cell line has therefore provided a useful source of murine TS. We report here six human lymphoblastoid cell lines (W1–L2: C1–C6) with acquired resistance (>10,000 fold) to the 2-$CH_3$ analogue of CB3717. This analogue is a 2-fold poorer TS inhibitor than CB3717, but is 40-fold more cytotoxic. It was hoped that these W1–L2 cells would overproduce TS in a manner similar to L1210 and thus be a source of human enzyme. Resistance was raised by subjecting the cells to incremental concentrations of the 2-$CH_3$ analogue of CB3717 and then cloning them in microtitre plates. The resultant cloned cell lines (W1–L2: C1–C6) were found to have increased TS activity (200-fold) compared with the parent line. TS gene amplification was also demonstrated by the use of a TS cDNA probe. DHFR activity in the cloned cell lines was not elevated compared with the parent line, and the DHFR gene was not amplified. Only a 40% decrease in TS activity was found when the resistant cells were grown in the absence of the 2-$CH_3$ analogue of CB3717 for >390 generations. The resistant line W1–L2:C1 was investigated further. This line showed cross-resistance to other folate-based TS inhibitors e.g., CB3717 and 2-desamino-CB3717 but was collaterally sensitive to the DHFR inhibitor methotrexate (MTX). The TS enzyme was partially purified from both the resistant and sensitive W1–L2 cell lines and Kiapps for the 2-$CH_3$ analogue of CB3717 were estimated to be $21.94 \pm 0.64$ nM (W1–L2:C1) and $22.3 \pm 0.516$ nM (W1–L2). It therefore seems unlikely that resistance is due to the TS enzyme having a decreased affinity for the 2-$CH_3$ analogue of CB3717. Moreover the Kiapps of a wide variety of folate-based TS inhibitors using TS from these resistant cells were similar to those obtained using TS from the mutant L1210 cells.

**The effects of the platinum anti-tumour agents on the renal response to secondary cytotoxic insult**

C. Ewen & J.H. Hendry

*Paterson Institute for Cancer Research, Wilmslow Road, Manchester M20 9BX, UK.*

Paterson BDF mice were treated with $LD_{50}$ doses of *cis*-platin ($15\,mg\,kg^{-1}$), iproplatin ($45\,mg\,kg^{-1}$) and paraplatin ($150\,mg\,kg^{-1}$). Animals surviving the period of acute toxicity were treated at day 14 with the acute nephrotoxin uranyl nitrate ($16\,mg\,kg^{-1}$) which induces tubule necrosis. The ability of the renal epithelium to recover from this injury was assessed by sub-capsular tubule count and thymidine autoradiography. In control animals uranyl nitrate reduced the sub-capsular tubule count from $508 \pm 5$ to $229 \pm 4$ 2 days after treatment. The sub-capsular count returned to control 7 days after treatment. Recovery was marked by an increase in cortical labelling indices from control levels of $0.21 \pm 0.03\%$ to $10.6 \pm 1.0\%$ by day 3. Treatment 14 days previously with iproplatin or paraplatin did not influence the renal response to uranyl nitrate, however, prior treatment with *cis*-platin had a potent inhibitory effect. 7 days after treatment with uranyl nitrate the sub-capsular count was $208 \pm 8$ compared to $490 \pm 23$ in the uranyl nitrate only group and $338 \pm 6$ in the cisplatin only group. The peak labelling index was $5.4 \pm 0.5\%$ in the *cis*-platin and uranyl nitrate group.

Three months after *cis*platin, there was still evidence of a reduced ability to recover from uranyl nitrate induced damage.

It is concluded that following $LD_{50}$ doses *cis*platin, unlike iproplatin and paraplatin, has a significant and long-term effect on the renal response to a secondary cytotoxic insult.

**Characterisation and reversal of weight loss induced by recombinant human tumour necrosis factor (TNF)**

S.M. Mahony & M.J. Tisdale

*CRC Experimental Chemotherapy Group, Pharmaceutical Sciences Institute, Aston University, Birmingham B4 7ET, UK.*

When administered as a single i.v. injection human recombinant TNF produced a dose-related weight reduction that was accompanied by and directly proportional to a decrease in both food and water intake. When given as two separate injections over a 24 h period, hypoglycaemia, hypertriglyceridaemia and a decreased level of circulatory FFA were observed 60 to 90 min after the final injection. The degree of hypoglycaemia is proportional to both the weight loss and the decrease in food and water intake.

In order to investigate the mechanism of the weight loss produced by TNF, animals were dosed orally with either water alone or a concentrated glucose solution. Weight loss was reversed by oral administration of water; the increase in weight being accompanied by an increase in total body water. Administration of a concentrated glucose solution had no greater effect than water alone in reversing the weight loss, indicating that the observed hypoglycaemia is not important in this action of TNF. Weight loss produced by TNF was also reversed by the i.p. administration of indomethacin (10 mg $kg^{-1}$) 2 h prior to the TNF injection. These results suggest that weight loss produced by TNF appear to be the result of prostaglandin production and dehydration.

### Circulatory lipolytic factors in cancer cachexia

S.A. Beck and M.J. Tisdale

*CRC Experimental Chemotherapy Group, Pharmaceutical Sciences Institute, Aston University, Birmingham B4 7ET, UK.*

We have utilised a transplantable colon adenocarcinoma of the mouse (MAC16) as a model of human cancer cachexia. This tumour produces extensive weight loss in the host at small tumour burdens (~2% of the total body weight) and without a reduction in either food or water intake. Weight loss is associated with a decrease in both carcass fat and muscle mass which is directly proportional to the weight of the tumour. Weight loss in this murine model has been correlated with the production by the tumour of both lipolytic and proteolytic factors, which are present in the circulation. Both factors respond to normal control and are inhibited by insulin and 3-hydroxybutyrate. Using DEAE cellulose chromatography and gel filtration the lipolytic and proteolytic factors have been shown to be distinct and to be separable into a number of fractions. Serum from cancer patients with extensive weight loss shows an elevated lipolytic activity when compared with normal subjects. Fractionation of serum from cancer patients using ion exchange chromatography shows evidence of lipolytic activity which elutes at the same ionic strength as that produced by the MAC16 tumour, while serum from normal control subjects contains no peaks of lipolytic activity in the same position. This raises the possibility that cachexia in both animals and humans may be due to circulatory lipolytic factors.

### Improved experimental anti-tumour activity of adriamycin by split dose scheduling

N.R. Sleigh, P.M. Loadman, M.C. Bibby & J.A. Double

*Clinical Oncology Unit, University of Bradford, Bradford, West Yorkshire BD7 1DP, UK.*

*In vitro* studies in this laboratory have shown increased cytotoxicity in the murine transplantable adenocarcinoma of the colon system (MAC) following prolonged exposure to the anthracycline adriamycin (ADR). This study investigates the anti-tumour activity and the pharmacokinetics of ADR following single and split dose scheduling *in vivo*. NMRI mice bearing ascitic or systemic MAC 15A or s.c. MAC 13 tumours were treated i.v. in groups of 10 with either one 10 mg $kg^{-1}$ dose of ADR or four doses of 2.5 mg $kg^{-1}$ at 2 h intervals. Median survival time (MST) or mean tumour weights were recorded as appropriate. MST was expressed as a per cent ratio of experimental over control time, and tumour weight as per cent inhibition of growth compared to the control group. Plasma was taken from non-tumour bearing mice at time intervals up to 24 h from commencement of each dose schedule and ADR was extracted by solid phase chromatography and analysed by HPLC. Drug concentration time curves were constructed and AUCs obtained as an indication of drug exposure. Treatment by both schedules produced the same effect in mice bearing ascitic MAC 15A with a T/C of 175%. However, in mice bearing systemic MAC 15A the single dose yielded a T/C of 180%, whilst the split dose produced >50% cures. The growth inhibition of MAC 13 following single and split dose schedules was 40% and 65% respectively. The single dose produced a peak plasma drug level of 24.95 $\mu g\, ml^{-1}$ and an AUC of 8.61 $\mu g\, ml^{-1}$. The corresponding values following the split dose schedule were 5.95 $\mu g\, ml^{-1}$ and 14.22 $\mu g\, ml^{-1}$ indicating increased exposure and reduced peak plasma concentration. The *in vivo* anti-tumour activity of ADR is enhanced following split scheduling of the total dose but any toxic effects on the host of this scheduling need to be determined.

### *In vitro* binding of nitrogen mustard to polyamino acids

A.M. Tacchi-Bedford & C.C. Boyle

*DHSS Department of Toxicology, St Bartholomew's Hospital Medical College, Dominion House, London EC1 7ED, UK.*

Various methods are currently being used for monitoring human exposure to alkylating agents. We are in the process of developing an analytical technique for quantitating the extent of exposure to such compounds as nitrogen and sulphur mustard by determining the formation of amino acid adducts in skin and hair samples. In order to identify the specific sites of reaction of these compounds with proteins, preliminary *in vitro* studies were carried out using polyamino acids as substrates. Nitrogen mustard ($HN_2$) was incubated with poly-L-histidine, poly-L-valine, poly-L-serine, poly-L-tyrosine, or poly-L-lysine in 0.1 M sodium phosphate buffer pH 7.4 at 37°C. The reactions were initiated by addition of 14C-labelled $HN_2$ ($10^6$ dpm, sp.a. 22 mCi $mmol^{-1}$) to the incubation mixtures and stopped with ice-cold ethanol. The polyamino acids were precipitated on glass fibre filters and the bound radioactivity was counted. The rates of reaction were similar for all polyamino acids: the maximum degree of binding was reached between 3 and 4 h from the beginning of the experiment and remained constant up to 6 h. The most reactive substrate was shown to be poly-L-valine with 3% of the initial radioactivity bound after 1 h, followed by poly-L-serine and poly-L-histidine (2 and 1.8%, respectively). Although these data do not necessarily reflect an *in vivo* situation, they may provide some information on potential target amino acids for $NH_2$ and the relevant adducts for future analytical work.

### Computer-aided design of N-(hydroxymethyl) anti-cancer drugs

W. Mallawaarachchi[1], D.E. Parry[1], R.J. Simmonds[1] & G.M. Wright[2]

[1]*Edward Davies Chemical Laboratories, U.C.W., Aberystwyth SY23 1NE; and* [2]*Chemical Design Ltd., Unit 12, 7 West Way, Botley, Oxford OX4 2UA, UK.*

Molecular orbital calculations for N-(hydroxymethyl)amides, including the anti-tumour N-(hydroxymethyl)formamide, have established that the half-lifes ($t_{1/2}$) of these hydroxymethylamides at pH 7.4 and 37°C may be reliably estimated from the N-$CH_2OH$ bond length, calculated using the MNDO method. Longer bonds are indicative of more reactive compounds and, as shown in the table below, this relationship is approximately maintained when extended to N-(hydroxymethyl)pentamethylmelamine (the active metabolite from hexamethylmelamine), trimelamol and 1-aryl-3-(hydroxymethyl)-3-methyltriazenes (e.g. the N-hydroxymethyl derivative of DTIC). Since the anti-tumour activity of these compounds is related to their half-lifes it should now be possible to assess the anti-tumour activity of analogues by molecular orbital calculations prior to synthesis.

| *Compound* | *N-CH₂OH bond length/Å* | $t_{1/2}$ *min*$^{-1}$ |
|---|---|---|
| N-(hydroxymethyl)phthalimide | 1.483 | 0.5 |
| N-(hydroxymethyl)dichloroacetamide | 1.477 | 77 |
| N-(hydroxymethyl)chloroacetamide | 1.475 | 1700 |
| N-(hydroxymethyl)formamide | 1.470 | 3000 |
| N-(hydroxymethyl)pentamethyl-melamine | 1.487 | 60 |
| 2,4,6-tris[N-hydroxymethyl)methyl-amino]-1,3,5-triazine | 1.488 | 10 |
| 1-(4-carbethoxyphenyl)-3-hydroxy-methyl-3-methyltriazene | 1.495 | ≫12 |

**Antibody-mediated radiosensitizer targetting of tumour cells-conjugate preparation**

J. Muir, H.M. Warenius & D.M. Tidd

*University of Liverpool, Department of Radiation Oncology, Clatterbridge Hospital, Bebington, Wirral, Merseyside L63 4JY, UK.*

The halogenated pyrimidine analogue 5-bromodeoxyuridine (BrdU) has proved to be an effective radiosensitizing agent in rapidly dividing cells. The magnitude of radiosensitization is proportional to the extent of BrdU incorporation into DNA, thus successful therapy would require high concentrations of BrdU to reach the malignant cells but not normal tissues. More specific cell targetting might be achieved by use of a BrdU-antibody conjugate, however, direct conjugation of drug to antibody has limited potential since only a small number of suitable functional groups are available for substitution if antibody-binding activity is to be retained. To increase the molar substitution of BrdU to antibody and thus the efficacy of the conjugate we have employed poly-L-lysine as a carrier molecule. BrdU is attached to the free -$NH_2$ groups of the polymer which is in turn linked to the antibody using the heterobifunctional cross-linking agent succinimidyl 4-(p-maleimidophenyl)butyrate in a molar ratio of antibody to SMPB of 1:5.

Spectrophotometric analysis of BrdU incorporation and measurement of the number of free -$NH_2$ groups in the substituted polymer show that ~40% of the -$NH_2$ groups are covalently coupled to BrdU. This results in a water soluble BrdU-carrier-antibody conjugate. Flow cytometric analysis has shown that the conjugate is incorporated into the human colonic adenocarcinoma cell line HT29/5. Further work is now being carried out to measure the degree of radiosensitization produced by the conjugate.

**Determination of rates of specific adsorptive endocytosis and non-specific endocytosis of drug antibody conjugates and their components**

M.C. Garnett, C.R.L. Graves & S.L. Smith

*Cancer Research Campaign Laboratories, University of Nottingham, Nottingham NG7 2RD, UK.*

For the development of targetted drug conjugates, it would be useful to account for the specific cytotoxicity of these compounds by the quantitation of their intracellular delivery. Assuming from the kinetics of uptake of antibody (see accompanying paper) and fluorescent conjugates that this is by pinocytosis, several possibilities for endocytosis exist. Either specific or non-specific adsorptive (predominant at low conjugate concentrations), and non-specific fluid phase (predominant at high conjugate concentrations).

The non-specific component has been measured by uptake of radiolabelled components into cells and expressed as an endocytic index (μl uptake of medium $10^{-6}$ cells $h^{-1}$), and the specific uptake of $^{125}$I-791T/36 antibody and $^{125}$I-791T/36-HSA-MTX conjugates have been determined using the quantitative radiolabelling method described in the accompanying paper. These results showed that uptake of HSA (used as a fluid phase marker) was at an average of 1.9 μl medium $10^{-6}$ cells $h^{-1}$, but that HSA-MTX was apparently taken up at 5.7 μl medium $10^{6}$ cells $h^{-1}$. The use of varying quantities of labelled material diluted with varying amounts of unlabelled material demonstrated a small but significant amount of membrane bound HSA-MTX. These data together with rates of antibody and conjugate endocytosis can be used to assess relative specific and non-specific endocytic uptake of drug-antibody conjugates.

**Flow cytometric estimation of relative bromodeoxyuridine incorporation as a means of detecting targetted bromodeoxyuridine in human and hamster cell lines**

D. Spiller, J.A. Tidd & H.M. Warenius

*University of Liverpool Department of Radiation Oncology, Clatterbridge Hospital, Wirral, Merseyside L63 4JY, UK.*

As part of a study to investigate the possibility of using antibodies to target BrdU for radiosensitisation, the colonic adenocarcinoma cell line (HT29/5) and a chinese hamster lung line (V79a) were exposed to 0, 3, 30 and 300 μmol BrdU for periods of time ranging from 30 min to 7 days. Relative BrdU incorporation was measured by a two stage fluorescence technique using a BrdU specific monoclonal antibody and flow cytometry. The biological effect of BrdU was measured using clonogenic and 3(4,5-dimethylthiazol-2-yl)-5-diphenyl tetrazolium bromide (MTT) assays. Following optimisation for each cell line of MTT test conditions, there was good correlation with the clonogenic assay in cell survival curves. There was also a correlation between cell survival and amount of BrdU incorporated. BrdU incorporation was shown in cells incubated with BrdU-polylysine conjugates thus allowing the potential feasibility of BrdU-polylysine-monoclonal antibody conjugates as vehicles for selective localisation of BrdU. The use of this technique is now being extended to confirm uptake of non-ionic oligonucleotide probes into whole cells by the use of BrdU within the nucleotide sequences.

**Biodistribution and tumour localisation of methotrexate and daunomycin monoclonal antibody conjugates in nude mice with human tumour xenografts**

M.V. Pimm, J.A. Clegg, M.C. Garnett, Y. Ogunmuyiwa, M.A. Paul & R.W. Baldwin

*Cancer Research Campaign Laboratories, University of Nottingham, NG7 2RD, UK.*

The blood kinetics and tumour localisation of conjugates of methotrexate (MTX) and daunomycin (DAU) and the monoclonal antibody 791T/36 have been examined in nude mice with human tumour xenografts. The antibody moiety of the conjugates were followed by labelling with 125-I and the drug moieties using radioimmunoassays.

After radioiodination, the drug moieties were co-precipitable with the radiolabel using TCA or rabbit anti-mouse IgG antiserum. Following i.v. injection, serum kinetics of both the antibody and drug moieties of both of the conjugates were essentially similar, and the integrity of serum-borne conjugate was confirmed by the co-precipitation of radiolabel and drug. The radiolabelled antibody moiety of the conjugate localised in tumour xenografts and the levels were maintained for up to 4 days. The declining serum levels and relatively constant tumour levels of radiolabel resulted in a progressive increase in tumour to serum ratios. Analysis of tumour levels of the drug moiety showed a similar pattern, with up to 4% of the injected dose being present per gram of tumour over the 4 day observation period with progressively increasing tumour to serum ratios of the drug, the ratios being virtually identical to those with antibody. Parallel studies with free drugs showed rapid clearance from the blood and a maximum of 0.35% of the dose/g of tumour 30 min after injection. Control immunoglobulin conjugated to MTX did not show tumour localisation of either the antibody or drug moieties.

These studies confirm the *in vivo* stability of these types of drug antibody conjugates and demonstrate site-specific targetting of therapeutic agents.

**Biodistribution of ricin toxin A chain-monoclonal antibody 791T/36 immunotoxin and influence of hepatic blocking agents**

Izabella Z.A. Pawluczyk[1], Vera S. Byers[1,3], R.W. Baldwin[1], M.V. Pimm[1] & A. Perkins[2]

[1]*Cancer Research Campaign Laboratories and* [2]*Department of Medical Physics, University of Nottingham, UK; and* [3]*Xoma Corporation, Berkeley, California, USA.*

An immunotoxin constructed by conjugating ricin A chain to monoclonal antibody 791T/36 specifically suppresses growth of human tumour xenografts and is in clinical trial for the treatment of colorectal cancer. The objective of immunotoxin therapy is to target the cytotoxic moiety (ricin A chain) to tumour cells and this requires that the product localizes efficiently in tumours. In this respect ricin A chain – 791T/36 antibody conjugates (RTA–791T/36) have a markedly altered biodistribution when compared to unconjugated antibody. This is principally manifest as hepatic uptake of immunotoxin which appears to be controlled by the ricin A chain (RTA) moiety. This was established by comparing the blood survival and organ distribution of immunotoxin with that of ricin A chain and free antibody using preparations in which either the RTA or antibody alone or as components of the immunotoxin were radiolabelled. Hepatic uptake is dependent upon Kupffer cell recognition of mannose-containing oligosaccharide structures on the RTA moiety of immunotoxin. Mannose-containing blocking agents given with immunotoxin were shown to prolong circulation time of the immunotoxin in blood and reduce liver uptake. Effective blocking agents include glycoproteins such as ovalbumin, ovomucoid and mannose-containing compounds including mannosyl-lysine. These agents specifically inhibit hepatic uptake of immunotoxin so altering biodistribution. Immunotoxin administered together with blocking agents preferentially localises in human tumour xenografts compared with immunotoxins alone and this should improve therapeutic efficacy.

**Investigation of the Reed–Sternberg/lymphocyte rosetting reaction**

D.J. Flavell & D.H. Wright

*Department of Pathology, Southampton General Hospital, Southampton SO9 4XY, UK.*

The association between Hodgkin tumour cells and T-lymphocytes to form rosettes is of unknown significance. Using 2 rosette-forming Hodgkin's disease derived cell lines (L428 and L591) we have studied the physico-chemical properties of rosettes formed *in vitro* and made some preliminary attempt to identify any putative receptor mediating adherence. Rosettes formed between the Hodgkin's disease cell lines and allogeneic peripheral blood lymphocytes were shown to be temperature independent, occurring optimally between pH5 to 8 and independent of active metabolic processes. The majority of the rosetting populations were T-cells (CD3+), comprised of equal numbers of $T_h$ and $T_s$ cells. B-cells were poor at forming rosettes and monocytes at least equally as good as T-cells. Removal of sialic acid from the Hodgkin's cell surface significantly reduced rosetting, but it was not possible to inhibit rosetting with N-acetyl neuraminic acid. Pre-incubation of L428 cells with peripheral blood membrane fragments significantly inhibited their rosette forming ability with allogeneic lymphocytes. In a preliminary attempt to identify any molecular structure(s) at the L428 cell surface, responsible for mediating adherence, we have demonstrated in binding assays the molecular weights of a number of $I^{125}$ labelled L428 surface components binding to peripheral blood membrane fragments.

**Peripheral blood mononuclear cells (PBM) from relatives of women with familial breast cancer form multinucleate giant cells (MGC) *in vitro***

A.R. Morton[1,2], N.G. Testa[3], S.A. Wallace[4], P. Burns[4], J. Birch[4] & A. Howell[2]

[1]*University Department of Medicine,* [2]*CRC Departments of Medical Oncology,* [3]*Experimental Haematology, and* [4]*Department of Epidemiology, Christie Hospital and Paterson Institute, Manchester, UK.*

When cultured *in vitro* human PBM fuse to form MGC which resemble foreign body giant cells and osteoclasts. This phenomenon has been shown to occur commonly in women with breast cancer (BC) but infrequently in unaffected controls (Al Sumidiae *et al.*, *Br. J. Surg.*, **73**, 839, 1986). The aim of this study was to determine whether MGC formation antedated the development of BC by culturing PBM from women at 50% lifetime risk by virtue of a strong family history (FH at least 2 affected first degree relatives). Ten ml peripheral venous blood was collected aseptically from 40 women with BC, 40 healthy control women with no FH of BC, and 20 first degree relatives of women with a strong FH of BC. PBM were separated over a Ficoll–Hypaque gradient. Without further purification $2 \times 10^6$ cells ml$^{-1}$ were incubated in Iscove's modified Dulbecco's medium plus 15% foetal calf serum at 37°C in an atmosphere of 5% $CO_2$/95% air. Medium was changed weekly. MGC (>3 nuclei/cell) began to appear after 7–10 days and cultures with >2MGC/590 $\mu^2$ were counted as positive. At 17 days the mean number of MGC in positive cultures was $24.2 \pm 5.0$ (s.d. $n=15$) and was $43.1 \pm 7.3$ at 24 days. MGC synthesised an osteoclast marker and tartrate resistant acid phosphatase. MGC formed in 30 of 40 (75%) cultures from women with BC, but only in 7 of 40 (17.5%, $0.001 < P \leq 0.01$) healthy age-matched controls with no FH of BC. MGC were seen in 14 of 20 (70%) of cultures from sisters (2 of 5) and daughters (12 of 15) of women with BC and a FH ($0.001 < P \leq 0.01$ compared with controls). These data suggest that the propensity to form MGC under these culture conditions may antedate the development of BC. Further investigation of this phenomenon as a possible marker of risk is warranted.

**_In vitro_ stimulation of B lymphocytes from peripheral blood in the production of human–human hybridomas**

H.Y. Youd, D. Spiller & H.M. Warenius

*University of Liverpool Department of Radiation Oncology, Clatterbridge Hospital, Bebington, Wirral, Merseyside L63 4JY, UK.*

An important part of the successful production of mouse–mouse hybridomas, has been the ready supply of stimulated B lymphocytes. In the human system it is necessary to stimulate B lymphocytes *in vitro* prior to fusion. This has been attempted using the mitogens phytohemagglutinin (PHA), as T cell mitogen, pokeweek mitogen (PWM), a T/B cell mitogen, and tetanus toxoid, as a means of B cell activation. Human peripheral lymphocytes were isolated from healthy volunteers. The total number of immunoglobulin (Ig) secreting cells and those specific for tetanus toxoid was assessed by the use of a filter plaque assay. In order to detect activated B cells within the mixed lymphocyte population, a flow cytometric technique simultaneously measuring DNA content and cell surface markers was adopted. Following mitogen stimulation the DNA profiles showed an increased number of cells in S $G_2$ M phase evident in both B and non-B cell populations. This was optimal after 72 h incubation with 1 $\mu$g ml$^{-1}$ PHA in 5% human AB serum at $1\times10^6$ cells ml$^{-1}$. Stimulation was found to be preferable in terms of fusion frequency and hybridoma production with PHA. The effect of B cell activation on specific Ig production is under continued examination before and after fusion.

### Posters

**Analytical and formulation studies on 1,4-bis(2′-chloroethyl)-1,4-diazabicyclo[2.2.1]-heptane dihydrogen dimaleate (NSC262666)**

M.J. Griffin, J.A. Slack & P. Kestell

*CRC Experimental Chemotherapy Group, Pharmaceutical Sciences Institute, University of Aston, Birmingham B4 7ET, UK.*

The synthesis and antineoplastic activity of a series of 1,4-bis(2′ - haloethyl) - 1, 4, diazabicyclo - [2.2.1] - heptane dications has been previously reported by Pettit *et al.* (*J. Pharm. Sci.*, **68**, 1539, 1979). The optimized HPLC conditions consisted of a phenyl column (100 mm x 8 mm) with a mobile phase of 0.3 mM sodium naphthalene sulphonate in water, adjusted to pH 1.9 with phosphoric acid. Detection was at 260 nm with the 1,4-bis(2′-chloroethyl)-1, 4, diazabicyclo-[2.2.1]-heptane ion eluting as a negative peak with a capacity factor (K′) of 0.33. The injection volume of 0.025 ml gave a lower limit of detection of 0.8 mg ml$^{-1}$ and calibration curves were linear over the concentration range 4.0 to 20.0 mg ml$^{-1}$. The coefficient of variation for replicate injections ($n=6$) was 5.1% at 2 mg ml$^{-1}$ and 4.0% at 10 mg ml$^{-1}$. The HPLC method was stability-indicating for NSC262666 with the reported degradation product, N,N′-bis(2′-chloroethyl) - piperazine, having a K′ of 0.72. Lyophilized preparations of NSC262666 proved to be both chemically and physically unstable at 50°C and room temperature. Aqueous solutions of NSC262666, sterilized by terminal filtration, were found to be stable at 4°C for greater than 12 months. An 8 mg ml$^{-1}$ solution of NSC262666 in 5% dextrose exhibited no degradation, at room temperature, over a period of 48 h. NSC262666 was formulated for phase I clinical trial as a 50 mg ml$^{-1}$ aqueous solution.

**2-desamino-2-substituted analogues of 10-propargyl-5,8-deazafolate (CB3717) as inhibitors of thymidylate synthase (TS)**

J.A.M. Bishop[1], A.L. Jackman[1], L. Hughes[2], P. Marsham[2], T.R. Jones[1] & A.H. Calvert[1]

[1]*Institute Cancer Research, Sutton, Surrey; and* [2]*I.C.I., Macclesfield, UK.*

The TS inhibitor, CB3717, was withdrawn from clinical study because of unpredictable liver and kidney toxicities. As the kidney toxicity, at least, was attributable to the poor water solubility of CB3717, 2-desamino CB3717 was synthesised in the hope that the reduced potential for H-bonding would result in a more soluble compound. This was indeed found to be the case and the compound was not acutely toxic to the liver and kidneys of mice. This fact, together with the good *in vitro* activity of this analogue, led us to make other modifications at the 2-position. A number of secondary and tertiary amines were synthesised, of which 2-$NHCH_3$ ($I_{50}=0.18\,\mu$M) and 2-$NH(CH_2)OH$ ($I_{50}=0.19\,\mu$M) were the most potent TS inhibitors. All were poor inhibitors of cell growth. A wide range of other 2-substitutions were made and we concluded that TS can tolerate quite large substituents at the 2-position although electron withdrawing or bulky groups confer poor cytotoxicity. The most potent TS inhibitors are shown below.

| *2-Substitution* | *TS $I_{50}$ ($\mu$M)* | *L1210 $IC_{50}$ ($\mu$M)* |
|---|---|---|
| -$NH_2$ (CB3717) | 0.02 | 3.4 |
| -$OLCH_3$ | 0.028 | 1.9 |
| -$CH_3$ | 0.04 | 0.085 |
| -$CH_2F$ | 0.096 | 0.37 |
| -$CH_2OH$ | 0.098 | 5.0 |
| -$SCH_3$ | 0.116 | 13 |
| -$CH_2CH_3$ | 0.136 | 2.5 |
| -$OCH_2CH_3$ | 0.146 | >100 |
| -H (desamino-CB3717) | 0.166 | 0.4 |
| -CL | 0.2 | 67 |
| -Phenyl | 0.22 | >100 |

The 2-$CH_3$ compound is a potent TS inhibitor as well as very cytotoxic and water soluble, properties that make this a very interesting compound for further study.

**Antibody inhibition of cholesterol 7α hydroxylase**

E.R. Eldredge[1,2], B. Jackson[1], K.E. Suckling[1] & C.R. Wolf[2]

[1]*Department of Cellular Pharmacology, Smith Kline & French Research Ltd, The Frythe, Welwyn, Herts AL6 9AR; and* [2]*ICRF Lab. Mol. Pharmacol. Department of Biochemistry, University of Edinburgh, Edinburgh EH8 9XD, UK.*

High levels of bile acid secretion have been associated with individual susceptibility to colon cancer. The rate limiting step for bile acid synthesis has long been accepted to be that catalyzed by cholesterol 7α hydroxylase. This minor species of cytochrome P-450 has been shown to be unique through substrate specificity studies and is not readily inducible by phenobarbital or 3-methylcholanthrene. We screened nine antibodies as inhibitors of cholesterol 7α hydroxylase, including those raised against cytochromes P-450 from the gene families I, IIA, IIC and III. Of three antibodies to proteins within family IIC ($PB_{1a}$, $PB_{1b}$ and $PB_{2a}$), an antibody raised against $PB_{2a}$, a male specific cytochrome P-450 expressed at adolescence, readily phosphorylated, and also not inducible by phenobarbital, could completely inhibit the microsomal 7α hydroxylation reaction. This finding, together with the fact that cytochromes P-450 are a large mutigene family, suggests that cholesterol 7α hydroxylase, being a unique P-450, may share some structural homology with other P-450's in the P450-IIC gene family.

### Some biological properties of 2-desamino-2-substituted-5,8-deazafolates that inhibit thymidylate synthase (TS)

A.L. Jackman[1], G.A. Taylor[1], R. Moran[2], J.A.M. Bishop[1], K. Pawelczak[1], K. Balmanno[1] & A.H. Calvert[1]

[1]*Institute Cancer Research, Sutton, Surrey, UK; and* [2]*Children's Hospital of Los Angeles, CA, USA.*

In the search for more potent and more water soluble analogues of CB3717 we have synthesised and tested a number of compounds possessing alternative substituents to the 2-amino function (Bishop *et al.* & Newell *et al.*, accompanying abstracts). The two best TS inhibitors made were the 2-$OCH_3$ and 2-$CH_3$-substituted compounds which were only 1.4 and 2-fold less active than CB3717 (Ki = 4 nM). The 2-$CH_3$ analogue was notably the most cytotoxic compound ($IC_{50}$ = 0.085 μM; 2-$OCH_3IC_{50}$ = 1.9 μM) although the activity of both could be prevented by co-incubation with thymidine suggesting that TS is their sole locus of action. Using a whole cell TS assay we demonstrated that the 2-$CH_3$ analogue (≧0.5 μM for 4 h) caused prolonged inhibition of TS after resuspension of cells in drug-free medium. This is assumed to be due to the formation of polyglutamate metabolites that cannot be effluxed from the cell. Indeed the 2-$CH_3$ analogue has substrate activity for folylpoylglutamate synthetase (FPGS) (Km = 60 μM) similar to that of CB3717 (Km = 40 μM) where polyglutamation in L1210 cells has been conclusively demonstrated. In contrast the 2-$OCH_3$ analogue could not continue to inhibit TS after the removal of extracellular drug (100 μM for 4 h) and this correlates with poorer FPGS substrate activity (Km = 193 μM) although slower transport into the cell may be an additional factor. Synthesis of synthetic polyglutamates of the 2-$CH_3$ compound has shown that they are substantially better inhibitors of TS (~100-fold) than the monoglutamate form (similar to that found with CB3717 polyglutamates). Intracellular metabolism to polyglutamates is an important determinant of cytotoxicity and all 2-desamino-2-substituted CB3717 analogues so far tested for FPGS substrate activity show activity that correlates well with cytotoxicity. Indeed polyglutamation seems to represent an essential activation step for many of the weaker TS inhibitors. For example 2-$CH_3$-5,8-dideazafolate is 112-fold less active than the propargylated compound described above but equally cytotoxic and this may be accounted for by its very good FPGS substrate activity (Km = 10 μM).

### Pharmacokinetic and toxicity studies with $C^2$-desamino $C^2$-substituted analogues of the antifolate $N^{10}$-propargyl-5,8-dideazafolic acid (CB3717)

D.R. Newell[1], R.J. Maxwell[2], J.R. Griffiths[2], G. Bisset[1], L. Hughes[3] & A.H. Calvert[1]

[1]*Institute of Cancer Research, Sutton, Surrey;* [2]*St Georges Medical School, Tooting, London; and* [3]*ICI Plc, Macclesfield, UK.*

In the search for a non-nephrotoxic and non-hepatotoxic antifolate thymidylate synthase (TS) inhibitor a series of $C^2$-desamino-$C^2$ one carbon substituted CB3717 analogues have been synthesised. Of these, the $C^2$-$CH_3$ analogue is only 2 fold less potent than CB3717 as a TS inhibitor yet 40 fold *more* toxic to tumour cells grown *in vitro*. Ther toxicities of CB3717 and analogues substituted at the $C^2$ position with -H, -$CH_3$, -$CH_2F$ and -$CH_2OH$ have been compared in mice. CB3717 (100 mg $kg^{-1}$ i.v.) caused both liver (raised alanine transaminase) and kidney (raised urea and creatinine) damage whilst the analogues induced no toxicity. This may be due to their greatly enhanced (> 100 ×) solubility at physiological pH. The pharmacokinetics of CB3717 (100 mg $kg^{-1}$ i.v.) and the $C^2$-H and $C^2$-$CH_3$ analogues (500 mg $kg^{-1}$) were compared in mice with sample analysis by HPLC. CB3717 was cleared slowly from the plasma (0.31 $h^{-1}$ $kg^{-1}$ $t^{1/2}$ 90 min) whilst the analogues were cleared rapidly ($C^2$-H 4.81 $^{-1}$h $kg^{-1}$ $t^{1/2}$ 21 min, $C^2$-$CH_3$ 2.51 $h^{-1}$ $kg^{-1}$ $t^{1/2}$ 16 min). For all three compounds 0–24 h urinary excretion accounted for 20–30% of the dose, however, faecal elimination of the two analogues was more extensive (50% dose 0–24 h) than with CB3717 itself (20% dose 0–24 h). The enhanced plasma clearance and faecal excretion of the analogues was probably a result of their lack of hepatotoxicity. Pharmacokinetics have also been studied non-invasively in mice (500 mg $kg^{-1}$ i.v.) and rats (100 mg $kg^{-1}$ i.v.) by *in vivo* $^{19}$F-NMR spectroscopy using the 2-$CH_3$-6′-$CH_3$ CB3717 analogue. In both species compound could be detected in the upper abdomen shortly after injection (rats < 2 min). As 70–80% of the dose was excreted in the bile within 1 h the signal was presumably due to compound undergoing biliary elimination. These studies have identified $C^2$-desamino-$C^2$ substituted analogues of CB3717 as non-hepatotoxic and non-nephrotoxic antifolates and have established further the viability of $^{19}$F-NMR in pharmacokinetic monitoring.

### *In vivo* inhibition of thymidylate synthase by $C^2$-desamino-$C^2$-substituted analogues of $N^{10}$-propargyl-5.8 deazafolic acid (CB3717)

G.A. Taylor, A.L. Jackman, K. Balmanno & A.H. Calvert

*Institute Cancer Research, Sutton, Surrey, UK.*

The evaluation of the efficacy of TS inhibitors in murine tumour systems is complicated by the high levels of circulating thymidine in the host mice. The amount of thymidine in mouse plasma is sufficient to circumvent the activity of TS inhibitors such that although TS may be effectively inhibited tumour cells may still, for a limited period, be able to replicate and synthesise new enzyme. Prolonged exposure is therefore essential to induce measurable antitumour response. The properties of $C^2$-desamino-$C^2$-substituted analogues of CB3717 vary with respect to TS inhibition, pharmacokinetics, and polyglutamation (Bishop *et al.*, Newell *et al.* & Jackman *et al.*, accompanying abstracts). The inhibition of TS activity within intact cells from mice following *in vivo* treatment with such analogues has been investigated with respect to dose and time. This has demonstrated the importance of the formation of non-effluxable

(assumed to be polyglutamated) forms of the analogues in determining the inhibitory activity seen *in vivo*. An i.v. dose of 240 mg kg$^{-1}$ of CB3717 was needed in order to achieve 50% inhibition of TS activity at 2 h in ascitic L1210 cells. The $C^2$-desamino (**A**) and $C^2$-CH3 (**B**) analogues were more effectively retained in cells than CB3717 despite their rapid clearance. A similar degree of inhibition was achieved with only 27 and 2 mg kg$^{-1}$ of **A** and **B** respectively. In order to obtain $\geqq$80% inhibition of TS (at 2 h) 430, 96 and 40 mg kg$^{-1}$ was required for CB3717 and the two analogues **A** and **B**. The extension of this degree of inhibition of TS over a 12 h period required a dose >1,000 mg kg$^{-1}$ of **A** but we found that <100 mg kg$^{-1}$ of **B** was sufficient. It is apparent that the multiple daily dosing of mice will not afford inhibition of TS throughout the treatment period. From these studies it is hoped that equipotent (with respect to TS inhibition) protocols may be defined which will permit the rational comparison of antitumour efficacy and toxicity of the analogues.

### A potential new *in vivo* metabolite of VP16 (etoposide)

H.P. Henneberry & G.W. Aherne

*Department of Biochemistry, University of Surrey, Guildford, Surrey GU2 5XH, UK.*

Methanol extracts of plasma from patients treated with VP16, *per os* or *i.v.*, were injected onto a 250 x 4.5 mm HPLC column packed with $C_{18}$, 5 $\mu$ diameter, silica gel and connected to a UV detector. Samples were eluted in methanol–water–glacial acetic acid (50:48:2) at a flow rate of 1 ml min$^{-1}$ and peaks were detected at 254 nm. Retention times ($R_T$) for *cis*-hydroxy acid VP16, VP16, VP16 aglycone and VM26 (Internal Standard) were 6.8, 9.2, 20.2, 22.7 min respectively. All patients' plasma extracts tested were found to have a peak corresponding to hydroxy acid VP16. Also, an unexpected late running peak of $R_T$ 31.6 min, not corresponding to any of the standards or analogues tested, and not present in drug-free plasma extracts, was seen in those plasmas.

Approximately 1 mg of this plasma component was collected and the results of preliminary analyses (organic spot tests, ELISA, IR, MS) revealed the component to be a non-immunoreactive aglycone related to the parent drug. Mass spectrometry indicated that the compound comprised two main fragment molecules of m/z 311 and 185. The compound did not contain quinone or semi-quinone groups but could possibly be a catechol derivative of VP16.

Recent work suggests that, besides its known interactions with topoisomerase II, VP16 may have an alternative mode of action involving conversion of the parent drug to cytotoxic intermediates. Several such potential intermediates have been identified *in vitro* but not yet *in vivo*. It remains to be determined whether our plasma component is an *in vitro* breakdown product or whether it is an as yet unidentified *in vivo* metabolite and, if so, whether or not it is cytotoxic.

### Bioreduction of *m*-Azidopyrimethamine (MZP) in mouse tissue homogenate

F.D. Kamali, A. Gescher & J.A. Slack

*CRC Experimental Chemotherapy Group, Pharmaceutical Sciences Institute, Aston University, Birmingham B4 7ET, UK.*

MZP, a potent lipophilic inhibitor of dihydrofolate reductase, is currently undergoing phase II clinical evaluation. It is an unusual drug molecule in that it contains an azido moiety. Azides are prone to chemical reduction, which in the case of MZP leads to *m*-aminopyrimethamine (MAP) and is accompanied by loss of anti-neoplastic activity. Here the hypothesis has been tested that the liver is the organ in which MZP is deactivated by enzymatic reduction. MZP (53 $\mu$M) was incubated in a final volume of 0.79 ml with hepatic homogenate, 12,000 *g* supernatant, mitochondria or microsomes (equivalent to 0.1 g liver wet weight) obtained from male BALB/c mice. MZP and MAP were measured by HPLC. MZP was rapidly metabolised to MAP in the whole homogenate and the postmitochondrial fraction, but not in microsomes or mitochondria. Within 30 min 79 $\pm$ 7% (mean $\pm$s.d., $n=9$) of MZP was reduced by the homogenate. Homogenates of the following tissues were also able to reduce MZP, values in brackets are the amount $\pm$s.d. ($n=3$) of MAP generated within 30 min as percentage of the amount of MAP found in liver homogenate: Kidneys (31.0 $\pm$ 1.2), heart (17.4 $\pm$ 3.4), intestine (6.7 $\pm$ 3.0), spleen (4.5 $\pm$ 1.0), and lung (3.1 $\pm$ 0.3). Reduction of MZP was also detected in tissue homogenate after heat-inactivation of enzymes, but it did not exceed 10% of the values quoted above. The results suggest that MZP is bioreduced in hepatic and extrahepatic tissue and that this reduction consists of a minor chemical and a major enzyme catalysed component.

### Assessment of serological assays for use in ovarian cancer (OCA)

J.E. Roulston[1], J. Fisken[1], A. Bowman[2] & R.C.F. Leonard[2]

[1]*University Department of Clinical Chemistry, Royal Infirmary, Edinburgh; and* [2]*University Department of Clinical Oncology, Edinburgh, UK.*

PLAP appears to be of little value as a serological marker used alone to monitor OCA (Fisken *et al.*, *Clin. Sci.*, **73**, (suppl. 17), 9, 1987). However its additive value in combined assays has been alluded to by other workers. We have assessed the value of sequential assays for CA125 and PLAP in the management of clinically advanced OCA. Both CA125 and PLAP activity (PLAP-A) and concentration (PLAP-C) were measured in 397 samples over time from 87 patients (FIGO I-7, II-5, III-55, IV-20). Active disease was present at the time of sampling in 261 instances. CA125 detected 73% (190/261) of the true positive (TP) samples with only 6 false positive (FP). When the remaining 71 samples were assayed by PLAP-A 40 were TP. Of the 31 'double negatives' 13 were TP for PLAP-C. Thus sensitivity apparently increases from 73% to 93% using all 3 assays. However, analysis of all 397 samples indicated significant numbers of FP with PLAP-A and PLAP-C. The predictive power of a positive result actually falls from 97% with CA125 alone to 76% by adding in PLAP assays. Similarly the FN rate for PLAP is high therefore predictive power of a negative result rose only from 65 to 76%. These data indicate that the PLAP assay is detrimental to the resolving power of CA125 alone despite attractive figures for apparent sensitivity.

### Urinary pseudouridine (PSU) levels in breast cancer

A. Hardcastle[1], W. Aherne[1], A.Y. Rostom[2] & R. Phipps[3]

[1]*University of Surrey, Guildford GU2 5XH;* [2]*St Luke's Hospital, Guildford GU1 3NT; and* [3]*Queen Alexandra Hospital, Portsmouth PO6 3LY, UK.*

Modified nucleosides such as PSU have been proposed as markers for several common solid tumours (Speer *et al.*, *Cancer*, **44**, 2120, 1979) but its value as a marker in breast cancer has not been extensively investigated. Urinary PSU

was measured by HPLC after preliminary isolation on Affigel columns (Kuo *et al.*, *J. Chromatogr.*, **145**, 383, 1978). We have confirmed that measurement of a random urine sample is comparable with a 24 h urine collection, if the PSU is expressed in relation to creatinine concentration, i.e. nmol PSU $\mu mol^{-1}$ Cr. Our laboratory normal range was 12.6–26.4 units. PSU levels do not display any circadian rhythm or alter through the menstrual cycle. To test the efficiency of PSU as a preclinical cancer marker, urinary levels were measured in 24 women who subsequently developed breast cancer each with 10 matched controls (Bulbrook *et al.*, *Breast Cancer Res. & Treat.*, **7** (Suppl.), 5, 1986). The urinary PSU levels in the 'pre-cancer cases' were not significantly different from the controls. We measured urinary PSU levels in patients before and after primary surgery for breast cancer and compared them with patients undergoing other major surgery. PSU levels were frequently not elevated above normal initially and did not fall after treatment. Also, PSUs were measured in 30 patients receiving post-operative radiotherapy for breast cancer; 43% had elevated urinary PSU (30–71 units). There was a non-significant tendency for higher levels with increased primary tumour size. PSU levels did not correlate with disease state as assessed by metastatic survey.

In other malignancies it has been reported that elevated excretion of PSU is a negative prognostic factor correlating with shorter survival time (Rasmuson *et al.*, *Bull. Mol. Biol. Med.*, **10**, 143, 1985). The importance of markedly increased PSU in individual breast cancer patients can only be determined by continued 'follow-up' of these subjects.

### Characteristics of a breast cancer antigen isolated from patients' sera

C. O'Sullivan & M.R. Price

*Cancer Research Campaign Laboratories, University of Nottingham, Nottingham NG7 2RD, UK.*

The anti-breast carcinoma monoclonal antibody, NCRC-11, defines a family of high molecular weight glycoproteins (>400 kD) associated with malignant cells and specialised normal secretory epithelia. These components have also been found to be elevated in the circulation of breast cancer patients.

Pooled serum from primary breast carcinoma patients was fractionated by affinity chromatography using Sepharose-linked NRC-11 antibodies. Non-specifically bound material, in particular human IgG and IgM, was separated from NCRC-11 defined antigen by gel filtration using Sephacryl S-300 chromatography after reduction of the sample with dithiothreitol, DTT (antigenic material being unaffected by DTT). Similar fractionation of a pool of serum from normal age and sex matched donors failed to produce any detectable NCRC-11 defined antigen.

The purified NCRC-11 defined antigen from patients' serum was characterised by SDS PAGE and Western blotting, by epitope mapping studies using a panel of reactive monoclonal antibodies and by enzyme digestion and lectin binding tests. The characteristics of this antigen from patients' serum were comparable to those of antigen preparations from primary tumour tissue.

### Plasma epirubicin estimation as a predictor of response in breast cancer

D.J. Kerr, G.J. Morrison, N. Lawson, J. Cassidy, E.M. Rankin & S.B. Kaye

*Department of Medical Oncology, University of Glasgow, UK.*

Although epirubicin is structurally similar to doxorubicin, it has a unique and extensive pattern of metabolism involving glucuronidation. There is little work relating the pharmacokinetics and metabolism of cytotoxic drugs to response or toxicity for a number of reasons: inconvenience of carrying out full pharmacokinetic studies; cost; problems with drug assay; insufficient patient numbers; unresponsive disease/ineffective drugs. A prospective randomised trial in advanced breast cancer comparing epirubicin at 2 doses (50 mg $m^{-2}$ and 100 mg $m^{-2}$) is underway locally. A single plasma sample was taken in 43 patients 30 min following bolus administration of epirubicin. Using a sensitive and specific HPLC assay it was possible to detect the parent compound and 5 metabolites. Nineteen patients are assessable for response (CR+PR) at 27 weeks following initiation of therapy; high dose, 6/9 response; low dose, 4/10 responses. Comparing high dose and low dose groups, there was no significant difference in parent drug concentrations (82 ng $ml^{-1}$ *vs.* 67 ng $ml^{-1}$) or in metabolite levels (total fluorescent compounds 241 ng $ml^{-1}$ *vs.* 203 ng $ml^{-1}$). There were no correlations between drug levels and responses although the single patient with a CR had a high epirubicinol level (more than 3 stand deviations from the mean). 9 patients have had multiple samples taken following identical doses and there was wide intra-individual variation in drug and metabolite levels from course to course.

Large patient numbers will need to be recruited to reveal any correlation between plasma drug concentration and response due to intra and inter individual variation in drug handling.

### Changes in proliferative activity in the normal human breast during the menstrual cycle and with age

G.T. Williams[1], R.J. Watson[1], M. Harris[2], C.S. Potten[3] & A. Howell[4]

*Departments of [1]Surgery, [2]Pathology, [3]Epithelial Biology and [4]Medical Oncology, Christie Hospital, Wilmslow Road, Manchester M20 9BX, UK.*

Events which occur early in the reproductive life (e.g. early menarche) of the female are associated with risk of breast cancer (BC) in later life. Korenman (*Cancer.*, **46**, 874, 1980) proposed that luteal phase progesterone (P) was antiproliferative and that the early onset of anovular cycles led to the unapposed stimulation of epithelial cells and thus increased risk. We have tested the hypothesis that P is antiproliferative by relating the labelling (LI), mitotic (MI) and apoptotic (AI) indices of histologically normal breast tissue to the day of the menstrual cycle, age and serum P values in 122 women undergoing opertions to remove fibroadenomas ($n=116$) or reduction mammoplasty ($n=6$). Thirty-three were taking the oral contraceptive pill. Thin tissue slices were incubated with tritiated thymidine (37 kBq $ml^{-1}$) and processed for autoradiography. More than 1,000 cells were counted to determine the LI, MI and AI for each sample. The LI ranged from 0–11.5% was cyclic with a maximum on day 20.8 and declined with age (0.7% per decade). The MI ranged from 0–0.6% with a maximum on day 21.5 and declined at a rate of 0.05% per decade. The AI was unrelated to the menstrual cycle but significantly declined with age. There was no significant effect of the contraceptive pill on the proliferative indices. Maximum LI was associated with high serum P values. These data indicate that the maximum proliferative rate of mammary epithelium occurs in the luteal phase of the cycle and suggests that P is not antiproliferative. They support the studies of Henderson *et al.* (*Cancer*, **56**, 1206, 1985) which show that the early onset of regular (and hence ovular) cycles are related to risk of BC.

**Verapamil resistance in a mouse tumour cell line**

P.R. Twentyman, N.E. Fox & K.A. Wright

*MRC Clinical Oncology and Radiotherapeutics Unit, Hills Road, Cambridge, CB2 2QH, UK.*

The calcium transport blocker, verapamil (VRP) is an effective modifier of resistance to adriamycin (ADM) and vincristine (VCR) in many multi-drug resistant (MDR) cell lines. In order to study the mechanism of such modification we have derived a VRP resistant subline (EMT6/VRP) of the mouse tumour cell line EMT6. Subline EMT6/VRP is 3.9 fold resistant to VRP ($ID_{50} = 94\,\mu g\,ml^{-1}$ cf. $24\,\mu g\,ml^{-1}$) but does not show cross-resistance to ADM or VCR. Similarly an MDR subline (EMT6/AR1.0) is resistant to ADM and VCR but not to VRP. Whereas EMT6/AR1.0 hyperexpresses the mRNA for P-glycoprotein, EMT6/VRP does not. The accumulation of $^3$H-VRP by EMT6/VRP is increased compared with the parent line whereas that by EMT6/AR1.0 is slightly decreased. The accumulation of $^3$H daunomycin on the other hand is increased 2 to 3 fold compared with the parent line in EMT6/VRP but is only 22% of the parent in EMT6/AR1.0. The sensitivity of all 3 cell lines to ADM is enhanced by the addition of 6.6 $\mu$M VRP (parent = 11.0 fold; EMT6/AR1.0 = 8.8 fold; EMT6/VRP = 6.1 fold).

We are currently studying the cross-resistance of EMT6/VRP to other resistance modifiers.

It appears that resistance to VRP occurs via a different mechanism to classical MDR and that VRP-resistance leads to a partial reduction in the ability of VRP to sensitise cells to ADM.

**Verapamil decreases the sensitivity to adriamycin of an adherent small cell lung cancer cell line *in vitro***

R. Milroy[1], J. Plumb[1], S.W. Banham[2] & S.B. Kaye[1]

[1]*University Department of Medical Oncology, Glasgow; and* [2]*Department of Respiratory Medicine, Royal Infirmary, Glasgow, UK.*

We have derived an adherent small cell lung cancer (SCLC) cell line from a skin metastasis of an untreated patient. This cell line grows in culture as a monolayer in Waymouth's medium supplemented with 10% FBS. This cell line has been fully characterised by pathological and immunocytochemical examinations and is tumorigenic in nude mice showing classical features of intermediate small cell lung cancer.

We have determined the chemosensitivity of this cell line to adriamycin by an assay based on the reduction of a tetrazolium dye, MTT. The $ID_{50}$ for a 24 h exposure to adriamycin was $3.0 \times 10^{-8}$ M. However, exposure to adriamycin for 72 h decreased the $ID_{50}$ by $\sim$50 fold to $6.0 \times 10^{-10}$ M.

The calcium antagonist verapamil has been shown to increase the sensitivity of some cell lines to adriamycin. However„ in this cell line exposure for 72 h to adriamycin in the presence of verapamil (6.6 $\mu$M) decreased the sensitivity by about 3 fold. In contrast, incubation of cells for 24 h in the presence of verapamil (6.6 $\mu$M) alone prior to a 24 h exposure to adriamycin and verapamil increased the sensitivity by $\sim$3 fold. Clearly this adherent SCLC cell line will be of value in our studies to elucidate the role of verapamil in the treatment of SCLC.

**Phenotypic stability of a tamoxifen resistant variant of the ZR-75-1 human breast cancer cell line**

H.W. Van den Berg[1], Maria Lynch[1], G.R. Dickson[2] & J. Nelson[3]

*Departments of* [1]*Therapeutics and Pharmacology,* [2]*Anatomy, and* [3]*Biochemistry, The Queen's University of Belfast, UK.*

We have maintained the ZR-751 human breast cancer cell line in the continual presence of tamoxifen (2–4 $\mu$M), for 2 years. After 6 months oestrogen receptor (ER), expression by tamoxifen exposed cells (designated ZR-75-9al), had fallen from $225 \pm 19$ to $56 \pm 12\,fmol\,mg^{-1}$ protein as determined using a whole cell binding assay. After a further 18 months routine culture in the presence of 4 $\mu$M tamoxifen both ER and progesterone (PGR), receptors had fallen to undetectable levels and this was accompanied by a marked reduction in sensitivity to antioestrogens.

Under phase contrast microscopy ZR-75-9al cells appear smaller than the parent line, tending to grow in 'islands' containing mounds of cells. Transmission electron microscopy revealed a marked reduction in intra-cellular lipid. Tonofilaments and desmosomes have not been observed. 9al cells maintained in medium devoid of tamoxifen or oestrogenic activity (phenol red free medium containing dextran charcoal stripped serum), remained ER and PGR negative and tamoxifen resistant for at least 3 months. In contrast, cells transferred to drug free 'complete' medium became ER and PGR positive and tamoxifen sensitive within one month.

We conclude that prolonged exposure of ZR-75-1 cells to tamoxifen does not result in the selective loss of ER positive cells but to a suppression of ER expression with consequent loss of tamoxifen sensitivity. Our data further suggest that recovery of ER and PGR levels only occurs in the presence of oestrogenic stimuli.

**Double-strand break induction and radiosensitivity in human tumour cell lines**

A.M. Cassoni, J. Eady, L.R. Kelland, T.J. McMillan & J.H. Peacock

*Radiotherapy Research Unit, Institute of Cancer Research, Clifton Avenue, Sutton, Surrey SM2 5PX, UK.*

Current evidence suggests that of the lesions induced in DNA by ionising radiation, double-strand breaks (dsb) are the most closely related to cell death. In support of this, recent data suggest that differences in radiosensitivity in rodent cell lines are related to differences in dsb induction (Radford, *Int. J. Radiat. Biol.*, **49**, 611, 1986).

We have investigated the induction of dsb in 3 human lung carcinoma lines and 3 human cervical carcinoma lines, of varying sensitivity. Irradiations were performed using $^{60}$Co $\gamma$-rays at a dose rate of 1–1.5 $Gy\,min^{-1}$ and cell survival measured using monolayer and soft agar clonogenic assays.

Dsb were measured following irradiation at ice-bath temperature using the neutral filter elution technique (pH 9.6). No significant differences were seen in dsb induction up to 45 Gy in tumour cell lines with Do 0.88–1.8 Gy. We conclude that within the limits of the commonly used neutral elution assay, induced dsb may not correlate directly with cell kill in human tumour lines. While this does not invalidate the idea that dsb may be the lethal lesion following exposure to ionising radiation, it does suggest that the processing of these lesions following induction may be the critical factor in determining radiosensitivity.

**Development of resistance in a human bladder cancer cell line by intermittent exposures to thiotepa**

L. Walker[1,2] & J.R.W. Masters[1]

[1]*Department of Pathology, The Institute of Urology, London WC2H 9AE; and* [2]*Department of Urology, St Thomas' Hospital, London SE1 7AE, UK.*

ThioTEPA is the agent most frequently used for intravesical treatment of superficial bladder cancer. One-third of patients enter complete remission; one-third, partial remission; and a third do not respond (Lum, *Rec. Res. Cancer Res.*, **85**, 3, 1983). Drug resistance, either present at the start of treatment or induced as a result is a major limitation of intravesical chemotherapy. We induced drug resistance *in vitro*, using a human urothelial cancer cell line, MGH-U1. A single clone was developed and used for these experiments. Drug response of the clone was measured by clonogenic assay, to select a concentration that produced 10–20% clonogenic cell survival after 1 h exposure. Three separate flasks were then treated by successive 1 h exposures to 40 $\mu g\,ml^{-1}$ of ThioTEPA. Ten exposures were carried out, at intervals of at least one week. The medium in three control flasks was changed in parallel. After 10 exposures, treated cells, parallel controls, and pre-treatment cells were compared in their response to 40 $\mu g\,ml^{-1}$ of ThioTEPA for 1 h. Mean percentage clonogenic cell survival was 22.2% $\pm$ 5.88 (s.d.) for pretreatment cells; 23.7% $\pm$ 7.22 for parallel controls; and 43.3% $\pm$ 3.16 for treated cells. The resistant cells are now being characterised in terms of isozyme phenotype, growth rates, and morphology.

The data indicate that 2 fold resistance was induced in a human urothelial cancer cell line by a regime of ThioTEPA treatment modelled on a course of intravesical chemotherapy.

**Evidence for 06-alkylguanine DNA alkyltransferase gene amplification in chloroethylating agent selected Chinese hamster lung fibroblasts**

J.E.N. Morten & G.P. Margison

*Paterson Institute for Cancer Research, Christie Hospital, Manchester M20 9BX and ICI Diagnostics Group, Gadbrook Park, Northwich, Cheshire CW9 7RA, UK.*

Chinese hamster V79 cells are very sensitive to the toxic effects of chloroethylating agents such as mitozolomide (Mz). The specific activity of 06-alkylguanine-DNA-alkyltransferase (ATase) in extracts of such cells is < $fmol\,mg^{-1}$ protein. In order to examine the extent to which Mz resistant cells within the population expressed higher levels of ATase, cells surviving Mz treatment were expanded and extracts of the cells were assayed for ATase activity. The resistant population was then exposed to a higher dose of Mz and the expansion and selection process repeated with incrementally increasing doses of Mz. ATase activity was found to increase with increasing selection dose and has so far attained >300 $fmol\,mg^{-1}$ protein at a dose of 100 $\mu g\,ml^{-1}$ Mz. No evidence of alkylphosphotriester ATase activity was found in extracts of these cells. The karyotype and DNA fingerprint of the selected population confirmed that they were derived from the parent cells. Double minute chromosomes were observed in Mz-selected cells. These increased in frequency with increasing selection dose and an average of 3 per cell occur in cells surviving 120 $\mu g\,ml^{-1}$ Mz. The stepwise increase in ATase specific activity and the occurrence of double minute chromosomes are consistent with ATase gene amplification.

**Regulation of glutathione S-transferases in rat liver by exogenous compounds**

C. Dolan[1,2], C.R. Wolf[2] & J.D. Hayes[1]

[1]*Department of Clinical Chemistry, Royal Infirmary, Edinburgh; and* [2]*ICRF Laboratory of Mol. Pharmacol., Department of Biochemistry, University of Edinburgh, Edinburgh, UK.*

Changes in the cellular levels of glutathione transferase (GST) enzymes appear to play an important role in preneoplasia and in the resistance of tumour cells to cytotoxic drugs. It is therefore important to understand the factors which regulate the levels of these compounds within cells. To this end we are studying GST regulation by both exogenous and endogenous compounds. Treatment of rats with phenobarbital (PB) and 3-methylcholanthrene (3-MC) leads to significant induction of hepatic Ya/Yc and Yb mRNA levels. As these compounds also induce cytochrome P-450, a multienzyme system involved in foreign compound metabolism, we have looked at the effect of a wide range of cytochrome P-450 inducing agents on GST mRNA levels in rat liver. The compounds used and their relative effect are given below:

| | Inducing agent | | | | |
|---|---|---|---|---|---|
| *GST subunit* | *AF* | *2AAF* | *ETOH* | *PB* | *DEX* |
| Ya/Yc | ++ | ++ | NC | ++++ | ++++ |
| Yf | NC | NC | NC | NC | ↓↓ |
| Yb | ++ | ++ | NC | ++ | ++ |
| *GST subunit* | *ARO* | *BA* | *αNF* | *βNF* | *CLO* |
| Ya/Yc | ++++ | ++ | + | +++ | NC |
| Yf | NC | NC | NC | ++ | NC |
| Yb | ++ | + | ++ | ++ | + |

It can be seen that many of the above compounds cause very significant changes in the levels of GST mRNA within rat liver. The Ya/Yc mRNA being regulated in a similar fashion to the Yb group. Of particular interest is the induction of the Yf subunit by βNF. This is an enzyme used as a preneoplastic marker and is associated with drug resistance. This is the first report of the regulation of this subunit by an exogenous organic molecule.

**Detoxication of peroxidized DNA by human and rat GSH transferases: Isolation of a form having high activity from rat liver nuclei**

K.H. Tan[1], D.J. Meyer[1], N. Gillies[2] & B. Ketterer[1]

[1]*CRC Molecular Toxicology Group, and* [2]*Department of Oncology, University College and Middlesex School of Medicine, Cleveland Street, London W1P 6DB, UK.*

Purified rat and human liver GSH transferases from the soluble supernatant fraction have activity towards DNA peroxidized aerobically by ionizing radiation. GSH transferases 5-5, 3-3 and 4-4 are the most active in the rat (500, 35 and 20 $nmol\,min^{-1}\,mg^{-1}$ respectively) and GSH transferases $\mu$ and $\pi$ the most active in man (80 and 10 $nmol\,min^{-1}\,mg^{-1}$ protein respectively). In rat liver nuclei two GSH transferase fractions were obtained, one extractable with saline/EDTA ('free') and the other with 8.5 M urea ('bound'). The 'free' fraction contained GSH transferase subunits 1 (40%); 2 (25%); 3 (5%); 4 (5%) and a subunit similar to 5 and referred to as 5* (25%). The 'bound' fraction contained principally subunit 5* (95%) and a small amount of subunit 6. A Se-dependent GSH peroxidase fraction from rat liver was also active towards peroxidized DNA, but since it accounts for only 20% of the free GSH peroxidase activity in the nucleus, GSH transferases may be more important. The results implicate a role for GSH peroxidases in DNA repair.

### Glutathione transferase isoenzymes in normal and neoplastic bladder tissue

J. Carmichael[1], A.L. Harris[1], K. Smith[1], D. Neal[1], J.D. Hayes, L.M Forrester[1], M. Glancy[1] & C.P. Wolf[1]

[1]*University Department of Clinical Oncology and Urology, Newcastle upon Tyne;* [1]*Department of Clinical Chemistry, Royal Infirmary, Edinburgh; and* [1]*ICRF Laboratory of Clinical Pharmacology and Drug Metabolism, Edinburgh, UK.*

The glutathione transferases (GST) represent a multigene family of isoenzymes which are important in the handling of xenobiotics including many cytotoxic drugs. They have been implicated in the development of drug resistance, and in addition increased levels of GSTs have been observed in pre-neoplastic states. Three main groups have been identified based on their separation by isoelectric focussing. GST isoenzyme activity was estimated in 32 human bladder samples: 16 invasive carcinomas, 12 superficial carcinomas and 4 normal samples. GST isoenzyme activity was estimated with the use of model substrates, chlorodinitrobenzene (CDNB) for overall activity, ethacrynic acid (EA) for the acidic forms and cumene hydroperoxide (CuH202) as an estimation of basic enzymes. A selection of these samples were further analysed by Western blotting, using antisera to 3 human GSTs. Mean CDNB activity in invasive tumour tissues was 163 (range 50–588), in superficial tumours 380 (26–802) and normal tissue 163 (128–236) nmol CDNB conjugated $min^{-1} mg^{-1}$ protein. There was an increase in CuH202 activity in selected tumours, but no difference with the acidic forms as determined using EA. Western blotting showed that the acidic isoenzymes were present at highest concentration and were present in all samples. A few tumour samples exhibited basic subunits with no significant neutral activity observed. Thus interesting differences in expression of GST isoenzymes were observed in superficial bladder tumours, although it should be emphasised that wide inter-individual variation in levels was observed. Acidic transferases were present in the greatest concentration as has previously been found in lung cancer samples. Basic activity was observed in some tumour samples, with minimal activity in normal tissue, and virtually no neutral isoenzymes seen.

### α-Interferon in combination with cytotoxic drugs against human lung carcinoma cell lines

R.J. Fergusson, S.P. Langdon, M.M. Hawkes & J.F. Smyth

*ICRF Medical Oncology Unit, Western General Hospital, Edinburgh, UK.*

Recent studies of α-interferon (IFN) in combination with certain cytotoxic drugs have indicated synergistic antitumour effects against human xenograft models in immune-deprived mice. This has been demonstrated for *cis*-platinum (CDDP)+IFN against human non-small cell lung carcinomas (NSCLC) (Carmichael *et al.*, *Cancer Res.*, **46**, 4916, 1986) and for adriamycin (ADR)+IFN against a human breast tumour (Balkwill *et al.*, *Cancer Res.*, **44**, 904, 1984). CDDP+IFN is currently under clinical assessment for the treatment of human NSCLC.

To investigate further the interaction between cytotoxic agents and IFN, we have studied the combinations of CDDP+IFN and ADR+IFN against human lung adenocarcinoma cell lines H23, H125 and A549 *in vitro*. Cells were plated on plastic at appropriate cell numbers in RPMI 1640+10% FCS. After 24 h, drugs were added for either 24 h or for 5 days. After 7 days colonies were counted. $IC_{50}$ values for the 3 cell lines were $>10^5\,U\,ml^{-1}$ for 24 h IFN; $10^2$–$10^5\,U\,ml^{-1}$ for 5 day IFN; 0.5–1.5 μM for 24 h CDDP; 25–80 nM for 5 day CDDP; amd 30–50 nM for 24 h ADR. All combinations of CDDP+IFN and ADR+IFN over either a 24 h or 5 day schedule were additive against the three cell lines when assessed by Steel's isobologram method (Steel, *Int. J. Rad. Onc. Biol. Phys.*, **5**, 85, 1979). Further studies are in progress examining these combinations *in vitro* against lung models where synergy has already been demonstrated *in vivo*.

### Phenotypic changes induced in lung adenocarcinoma cell lines by sodium butyrate

S.P. Langdon, M.M. Hawkes, F.G. Hay & J.F. Smyth

*ICRF Medical Oncology Unit, Western General Hospital, Edinburgh, UK.*

We have examined the effects of sodium butyrate (SB), an agent capable of inducing differentiation in many systems, against 3 human lung adenocarcinoma cell lines (H23, H125 and the alveolar line A549). It has recently been shown that the monoclonal antibodies (MoAbs) 123C3 and 123A8, raised against a small cell lung carcinoma (SCLC), react with approximately 100% SCLC (25/25 positive) but are generally unreactive with lung adenocarcinoma sections (1/27 positive) (D. Schol *et al.*, *Br. J. Cancer*, **56**, 519, 1987). SB induced both these markers in these cell lines (Table below) after 4 days exposure at cytostatic doses (>3 mM). Alkaline phosphatase was also induced in these cells at these doses (Table). These phenotypes were determined by (immuno-) histochemical staining.

% Cells +ve

| | *A549* | | | *H23* | | |
|---|---|---|---|---|---|---|
| *Phenotype* | *0 mM* | *3 mM* | *5 mM* | *0 mM* | *3 mM* | *5 mM* |
| 123C3 | 0 | 55 | 68 | 0 | 31 | 35 |
| 123A8 | 0 | 63 | 64 | 0 | 60 | 38 |
| Alk Phos | 2 | 51 | 51 | 1 | 9 | 38 |

| | *H125* | | |
|---|---|---|---|
| *Phenotype* | *0 mM* | *3 mM* | *5 mM* |
| 123C3 | 0 | 75 | 98 |
| 123A8 | 0 | 63 | 93 |
| Alk Phos | 1 | 27 | 36 |

Both 123C3 and 123A8 are members of a group of 11 MoAbs (defined as Cluster 1) active against SCLC which show strong neural activity (R.L. Souhami *et al.*, *Lancet*, **8554**, 325, 1987). Other members of Cluster 1 were not induced in these cell lines after exposure to SB. The effect of SB on other possible differentiation markers in these cell lines is currently being examined.

**Oestrogen receptor expression and effects of tamoxifen against human ovarian carcinoma cell lines**

M.M. Hawkes[1], S.P. Langdon[1], S.S. Lawrie[1], R.A. Hawkins[2], A. McDonald[1], A. Tesdale[2] & J.F. Smyth[1]

[1]*ICRF Medical Oncology Unit, Western General Hospital and* [2]*University Department of Surgery, Royal Infirmary, Edinburgh, UK.*

A number of clinical studies have examined the role of anti-oestrogen (E) therapy for ovarian cancer. While over 50% ovarian tumours possess oestrogen receptors (ER), clinical response rates with the anti-oestrogen tamoxifen (T) are reported to be between 0 and 36%. To assess the importance of ER for growth and as a possible target for therapy in this disease, we have examined the effects of T against a series of 8 ovarian cancer cell lines. The ER content of the cell lines was determined by a dextran–charcoal adsorption assay. The series of cell lines PEO1, PEO4 and PEO6 possessed high values of ER (60–200 fmol mg$^{-1}$ protein), PEA1 and PEA2 had low values (10–30 fmol mg$^{-1}$) and PEO14, TO14, PEO23 were ER − ve. Examination of different passages of PEO4 cells indicated that ER values increased with passage number for this cell line. All 8 cell lines were relatively insensitive to the effects of T. $IC_{50}$ values of the cell lines in a colony forming assay on plastic were all ~8 $\mu$M for a 72 h exposure to T, independent of their ER status. Doses of > 8 $\mu$M T (over 7 days) were cytostatic to all lines (cf. breast ca cell lines sensitive at doses of < 1$\mu$M). The ER + ve line PEO4 grew more rapidly in the presence of added E and more slowly on removal of E.

Vimentin expression (VE) has been suggested to be an indicator of progression to hormone-independence in a hormone-dependent malignancy (Agnor *et al., Proc. AACR*, **28**, 11, 1987). For these 8 cell lines an inverse correlation was found between ER and VE.

**An *in vitro* assay of cell viability based on staining fixed cells with alcian blue**

J.A. Hanson, E.A. Bean & J.L. Moore

*Radiation Sciences Laboratories, Velindre Hospital, Cardiff, UK.*

The differential staining of cells with a vital dye can be carried out on fixed cells (Yip & Auersperg, *In Vitro*, **7**, 323, 1972). This finding along with the publication of the 'DiSc' assay for determining *in vitro* chemosensitivity of haematological malignancies (Bird *et al*, *Leukemia Res.*, **10**, 445, 1986) caused us to consider the possible use of an alcian blue based dye exclusion assay on previously fixed cells.

Following treatment cells (Molt4 and Daudi) were cultured for 4 days when they were fixed in glutaraldehyde in phosphate buffer. They were left in iced water for 1 h and subsequently at 4°C. The samples were stained with alcian blue including duck red cells (internal standard) and slides prepared using a cytospin centrifuge. We found that with cell lines counterstaining was unnecessary and slides could be rapidly prepared by dehydrating in ethanol followed by clearing in xylene and mounting with Canada balsam. Where counterstaining was required we used Giemsa followed by the above procedure.

Using two cell lines we have obtained dose response curves following radiation and drug (Adriamycin and AraC) exposure in our dye exclusion assay, a soft agar clonogenic and MTT assay. We consider this ability to fix cells for examination/processing at a later date to be of general use. It is also preferable to deal with fixed samples where possible. For the specific application of *in vitro* viability studies, it is limited to suspension cultures as clumping may be a practical problem for other cell types. A pilot study with CLL samples to assess cell/drug response is currently in progress.

**Establishment and characterisation of small cell lung cancer cell lines**

R. Milroy[1], J. Plumb[1], W. Candlish[3], M.R. Adamson[3], S.W. Banham[2] & S.B. Kaye[1]

[1]*Departments of Medical Oncology, University of Glasgow; and Departments of* [2]*Respiratory Medicine and* [3]*Pathology, Royal Infirmary, Glasgow, UK.*

Small cell lung cancer (SCLC) is usually chemoresponsive at outset but relapse is a frequent occurrence. Such relapse tumour is often chemoresistant. Comparison of cell lines derived from pre-treatment and from relapse tumours may give an insight into the mechanisms of drug resistance. Thus, we have successfully established five SCLC cell lines *in vitro* from 17 biopsies (1/7 bronchial biopsies taken via the fibre-optic bronchoscope and 4/10 metastases). Three pre-treatment lines have been established from: (a) an endobronchial biopsy from a subsequently chemoresistant patient (LS106), (b) a metastatic node deposit from a subsequently chemosensitive patient (LS111) and (c) a cutaneous metastasis from an untreated patient (LS112). Two further cell lines of biopsies obtained from a patient who relapsed twice following chemotherapy have been established following passage in athymic nude mice. These lines grow as floating aggregates of cells in RPMI 1640 culture medium supplemented with FBS (2.5%), selenium, insulin and transferrin. A sub-line of LS112 grows as a monolayer culture in Waymouth's medium containing FBS (10%). The lines show features of human SCLC on pathological examination, express NSE and CAM 5.2 and contain both dopa-decarboxylase and creatine kinase (BB) activities. Chemosensitivities were measured in these cell lines by an assay based on the reduction of a tetrazolium dye, MTT. We found that the cell lines demonstrate a wide range of chemosensitivities to adriamycin ($10^{-8}$ to $10^{-7}$M) and this suggests that they will prove useful models in the study of drug resistance *in vitro*.

**Effect of Tween 80 on the disposition of thioTEPA given intravesically**

B.J. McDermott[1], J.R.W. Masters[2], P.J.R. Shah[2], E. Fenwick[2], P.M. Loadman[3] & M.C. Bibby[3]

[1]*Department of Therapeutics and Pharmacology, The Queen's University of Belfast;* [2]*Institute of Urology, University College London; and* [3]*Clinical Oncology Unit, University of Bradford, UK.*

The uptake of a number of chemotherapeutic agents and their cytotoxicity are enhanced by Tween 80 *in vitro* and *in vivo*. Added to ThioTEPA, the non-ionic detergent significantly reduced the colony-forming ability of a human bladder cancer cell line (Parris *et al., Urol Res.*, **15**, 17, 1987). Therefore, this combination might be more effective than drug alone for intravesical chemotherapy of superficial bladder cancer, although Tween 80 could increase the systemic absorption of ThioTEPA and so potentiate its myelosuppressive effects. A pharmacokinetic study using a randomised cross-over design was performed in which 8 patients with multiple recurrent stage pTa or pT1 disease were given ThioTEPA (30 mg) in distilled water or in 10% (v/v) Tween 80 (30 ml) for 2 h, followed 3 months later by the alternative treatment. Large variation occurred in the proportion of ThioTEPA absorbed (20–78%), but the rate of absorption was not affected by Tween 80. Peak plasma levels, 101 and 154 ng ml$^{-1}$, were observed within 1 h of administration, concentrations fell with half-lives of 1.83 and 1.25 h and AUC values were 0.376 and 0.496 $\mu$g h ml$^{-1}$ with drug alone and in combination with Tween 80 respectively: the difference between these mean values were all significant ($P<0.05$). These results indicate an apparent reduction in the volume of distribution of ThioTEPA when administered with Tween 80. WBC count decreased significantly 14 days following treatment only after the therapy including Tween 80. It is concluded that the maximum safe dosage would be considerably less than that with drug alone if Tween 80 were added to intravesical chemotherapy with ThioTEPA.

### A phase I–II study of ifosfamide in combination with adriamycin in the treatment of adult soft tissue sarcoma

J.L. Mansi, S. MacMillan, C. Fisher & E. Wiltshaw

*The Sarcoma Unit, The Royal Marsden Hospital, Fulham Road, London SW3 6JJ, UK.*

To evaluate the two most active drugs in the treatment of soft tissue sarcomas 54 patients were treated with a combination of ifosfamide and adriamycin at a dose of 5 g m$^{-2}$ and at least 40 mg m$^{-2}$ respectively at 3 weekly intervals. Of the 50 evaluable patients a response was seen in 11 (22%) patients (3 complete responses and 8 partial responses), stabilisation of disease occurred in 17 patients and the remaining 22 patients progressed whilst on treatment. Of the 22 patients receiving adriamycin at 60 mg m$^{-2}$ 12 (55%) required a dose reduction due to toxicity compared to 11 (39%) of the 28 patients who received 40 mg m$^{-2}$. For the patients who had a response the median relapse-free interval was 7 months (range 2–17+) and the overall median survival was 12 months (range 5–29+). The combination does not appear to increase the response rate above that of single agent chemotherapy.

### Guanidibenzoatase activity of tumour cells and the regulation of tumour invasion

F.S. Steven[1], M.M. Griffin[1] & H. Maier[2]

[1]*Department of Biochemistry and Molecular Biology, University of Manchester, Manchester M13 9PT, UK;* [2]*E.N.T. Clinic, Justus-Liebig University, Gissen, FRG.*

We have examined 55 tumour bearing tissues taken from the head and neck regions as frozen sections. We employed 9-aminoacridine as a fluorescent probe for cells possessing the tumour associated protease guanidinobenzoatase. We observed that most of the cells within the tumour masses in these sections did not bind 9-aminoacridine and therefore possessed inhibited enzyme which was subsequently exposed by displacement of the inhibitor. We noted that the cells at the advancing edge of the tumour masses and individual tumour cells in the stroma bound 9-aminoacridine and fluoresced. Guanidinobenzoatase is known to degrade fibronectin and we believe that those cells possessing uninhibited guanidinobenzoatase *in vivo* are capable of migration and tissue invasion. We observed that guanidinobenzoatase in solution and on the surface of these fluorescent tumour cells was inhibited by $10^{-6}$ M bis-(carbobenzoxy-arginine)-rhodamine in an irreversible manner. This synthetic inhibitor had no action on trypsin or cell-bound guanidinobenzoatase which was already complexed with the normal inhibitor in these frozen sections. We conclude that the activity of tumour cell guanidinobenzoatase is regulated *in vivo* by the naturally occurring inhibitors. Only those cells at the advancing edge of the tumour mass possessed active enzyme and this could be selectively inhibited by a synthetic arginine derivative.

### Provisional assignment of ecto-5′-nucleotidase to human chromosome 6

Y. Hey, M. Fox & J.M. Boyle

*Paterson Institute for Cancer Research, Christie Hospital and Holt Radium Institute, Manchester M20 9BX, UK.*

Ecto-5′-nucleotidase (5′-NT) is a glycosylated protein consisting of a dimer of subunit 70 kD bound by glycosyl phosphatidyl inositol linkage to the external plasma membrane of mammalian cells and to microsome and Golgi membranes. The enzyme converts 5′ purine nucleotides to nucleosides, AMP being the preferred substrate. Specific activity increases during differentiation of B and T cells and consequently the enzyme is used as a marker to distinguish differentiation arrested leukaemias (low 5′-NT e.g. T-acute lymphoblastic leukaemia) from end cell leukaemias (high 5′-NT, common acute lymphocytic leukaemia and blast crisis in chronic myelogenous leukaemia). Elevated 5′-NT has also been observed in cells resistant to purine analogues.

Using a panel of 26 human × hamster hybrids and a rapid assay of 5′-NT on whole cells we observed 92% concordance of 5′-NT with the presence of human chromosome 6. The 2/26 discordant cell lines were subsequently shown to have been erroneously karyotyped with respect to chromosome 6. The HLA Class I genes are carried on human 6p and the Class I antigen is recognised by the murine monoclonal antibody W6/32. Human × hamster hybrids that contain chromosome 6 fluoresce weakly when stained with W6/32 and rabbit anti-mouse FITC enabling HLA$^{+}$ and HLA$^{-}$ cells to be sorted by FACS and cloned. In all hybrids and segregants tested there was 100% concordance in the expression of HLA$^{+}$ 5′-NT$^{+}$ confirming the linkage of a gene controlling 5′-NT expression to human 6.

### Lectin-binding affinities in human primary breast tumours and axillary lymph node metastases

D.T. Rîsniţă

*Institute of Oncology, Department of Immunochemistry, 3400 Cluj-Napoca, Romania*

The role of carbohydrate structures of the cell membrane surface in immunogenicity, cell adhesion and proliferation is well known. Using several fluorescein- or horseradish peroxidase-labelled lectins, we and others have demonstrated their binding in breast carcinoma cells. Recently (Leathem & Brooks, *Lancet*, **8541**, 1054, 1987) the binding of Helix Pomatia (HPA) has been shown to have some value in prognosis. In this study we used a panel of peroxidase-conjugated lectins (Con A, WGA, SBA). On paraffin-embedded sections obtained from 30 primary breast tumours and from their axillary lymph node metastases respectively, we analyzed the difference in lectin-binding between primary tumour and its metastases as well as the significance of this difference. Computerized image analysis was used to evaluate the intensity of positive staining. At the level of the malignant breast, cell membrane alterations were present which allowed specific lectin binding. In some cases this binding had different patterns and was more intense in metastases than in the corresponding primary tumours, suggesting well-marked alterations of cell-surface carbohydrate structures. In breast cancer, lectin histochemistry, useful in micrometastasis identification, may allow us to gain further insight into an understanding of the metastatic process.

**An inducible clonal marker system to investigate mutations and clonal organisation in gut epithelia**

B.A.J. Ponder, M.A. Blount & D.J. Winton

*Institute of Cancer Research, Haddow Laboratories, Clifton Avenue, Sutton, Surrey SM2 5PX, UK.*

C57BL/6J (B6) mice express a binding site for Dolichos biflorus agglutinin (DBA) on gut epithelium, SWR mice do not. This difference is specified by alleles at a single locus. DBA binding is dominant; thus B6 × SWR F1 mice possess a single allele which determines DBA binding, and the gut epithelium of these F1 mice can be stained in whole mounts using DBA-peroxidase conjugate. In animals killed 2 weeks after a single dose of the mutagen ethylnitrosurea (ENU), however, negatively staining ribbons of epithelial cells are seen arising from the gut floor and ascending to the tips of the villi. We interpret these as clones originating from mutated crypt stem cells which have lost the DBA binding allele. The evidence for this is: (1) the putative clonal patterns are similar to those we have previously described in B6↔SWR chimaeric intestine; (2) the DBA −ve clonal patterns can be initiated during foetal development and persist as 'descendent clones' in the adult; (3) no DBA −ve clones are seen in homozygous DBA +ve B6 control mice; (4) a dose-response relationship is observed with increasing doses of ENU; and (5) the number of events observed (e.g. 20–30 per $10^4$ villi 2 weeks after a single dose of ENU 50 mg kg$^{-1}$) are consistent with somatic mutation. Partially or wholly mutated intestinal crypts can also be scored in whole mounts of small intestine and colon. This novel technique can be used to investigate the clonal organisation of the crypt and the occurrence and persistence of 'spontaneous' or induced mutations at different levels of the crypt stem cell hierarchy under different conditions.

**The possible use of serum MHC class II levels for monitoring graft-*versus*-host disease in bone-marrow transplant recipients**

S. Thompson[1], M. Wareham[1], A.D.J. Pearson[2], L. Sviland[2] & G.A. Turner[1]

*Departments of [1]Clinical Biochemistry and [2]Child Health, The Medical School, Framlington Place, Newcastle upon Tyne NE2 4HH, UK.*

Bone-marrow transplantation (BMT) is increasingly being used to treat leukemias and lymphomas. Detection of graft-*versus*-host disease (GVHD) in these patients is a difficult and time-consuming task that requires intensive patient investigation. As HLA Dr is expressed on keratinocytes and enterocytes in GVHD (Sviland *et al.*, *Bone Marrow Transpl.*, **3**, 408, 1987), measurement of blood levels of class II molecules could form the basis for a new test for monitoring GVHD. Class II levels were measured in sera from healthy individuals and from 26 patients who received either an allogeneic or an autologous BMT as part of their therapy. Specimens from the latter group were taken prior to transplantation (A) between one and four weeks after treatment (B) and on two further occasions up to 6 months later (C, D). In 20% of specimens it was impossible to measure class II levels because of interference by rheumatoid factors. Although insufficient serial specimens were available for many of the patients, certain preliminary observations can be made. Class II molecules were detectable in 6/22 healthy individuals (mean − 0.49 U ml$^{-1}$) and 17/26 group A patients (mean = 2.32 U ml$^{-1}$) and these groups were significantly different ($P$ = 0.002). Following grafting, class II levels increased further in 12/15 patients (A *vs.* B, $P$ < 0.05). At later times (C and D), levels changed according to the type of graft. With autologous grafts and allogeneic grafts without GVHD (7 patients), levels progressively decreased to values similar to or less than those in group A. In 4 patients with GVHD following allogeneic BMT, levels tended to remain elevated. It is concluded that measurement of serum class II levels may be useful for monitoring GVHD but further work is needed to establish this approach.

**Lack of free-radical formation by a doxorubicin-oestrogen conjugate**

N.G. Hartman[1], L.H. Patterson[2], A. Suarato[3], F. Angelucci[3], P. Workman[1] & F. Arcamone[3]

*[1]Cambridge University Department of Clinical Oncology and Radiotherapeutics, Cambridge CB2 2QQ; [2]School of Pharmacy, Leicester Polytechnic, Leicester LE1 9BH, UK; [3]Farmitalia Carlo Erba, Milano, Italy.*

Drug-targeting provides a means to reduce drug toxicity. Increased expression of oestrogen receptors on mammary tumour cells affords a tumour-specific drug-targeting strategy. The cardiac toxicity of the anthracyclines could be due to free-radical generation. We have synthesized a novel oestrone carboxymethoxylamine doxorubicin conjugate and evaluated its capability to generate such toxic species, using rat liver microsomes, in comparison with parent doxorubicin. The oestrogen conjugate had a minimal effect on the basal rate of the liver microsomal NADPH-consumption (10.7 ± 1.1 nmol min$^{-1}$ mg$^{-1}$ protein); or liver microsomal superoxide anion generation, determined via acetylated cytochrome c reduction (20.2 ± 0.8 nmol min$^{-1}$ mg$^{-1}$ protein), or adrenochrome formation from adrenaline (30.2 ± 2 nmol min$^{-1}$ mg$^{-1}$ protein). In contrast, doxorubicin displayed a concentration-dependent increase above basal rates for all these parameters. No esr signal was generated in NADPH-fortified microsomes when the conjugate was incubated anaerobically for up to 30 min. Under identical conditions an esr spectrum for the doxorubicin quinone free-radical was observed, which became more anisotropic with time. In addition, the conjugate inhibited the free-radical esr spectrum of parent doxorubicin. These results suggest that the oestrone carboxymethoxylamine doxorubicin conjugate is not metabolically reduced to a free radical intermediate in rat liver microsomes, thus eliminating this as a mechanism of toxicity. In addition, the conjugate inhibits the free-radical formation of doxorubicin, providing a positive guideline towards future anthracycline development or hormone-drug combination therapy.

**Increase in tumour growth after blood transfusion**

P. J. Clarke, K.J. Wood & P.J. Morris

*Nuffield Department of Surgery, John Radcliffe Hospital, Oxford, UK.*

There is debate as to whether peri-operative blood transfusion is associated with a poorer prognosis in patients undergoing tumour resection. The results of retrospective clinical studies are conflicting and difficult to interpret. Preliminary experiments in a mouse tumour model indicated that spontaneous metastases from a subcutaneous primary were augmented when the animals were transfused with allogeneic blood 14 days before tumour inoculation. Further studies have now investigated the effect of both the timing of the transfusion and the strain of the donor blood.

Groups of C3H (H2k) mice were transfused with 0.25 ml of C57/B16 (H2b) blood at days −14, −7, 0 and +7 in relation to an inoculum of UV-2237 fibrosarcoma cells at day 0. Control groups were transfused with syngeneic blood at each time point. Animals were all killed at day +21 and those transfused with allogeneic blood on days −14, −7 and +7 showed a significant increase in the number of pulmonary tumour deposits compared with controls. This effect was dependent on the dose of tumour inoculated, being most marked when the injected dose was near the minimum dose needed for tumour growth (MTD).

In further experiments, groups of mice were inoculated with UV-2237 cells and on day +7 were transfused with blood from donors which varied both at the major and/or the minor histocompatibility antigen loci. At day +21 the mice were killed to assess lung colonisation and the results showed that the augmentation of tumour deposits by blood transfusion was dependent on the donor strain and suggested that differences in both major and minor histocompatibility antigens may be involved.

### Use of a monoclonal antibody-mediated haemadsorption method to identify tumour cell colonies in mixed cultures

M.J. Embleton, K. Affleck & A. Capuano

*Cancer Research Campaign Laboratories, University of Nottingham, Nottingham NG7 2RD, UK.*

A method involving adherence of monoclonal antibody (MoAb)-coated sheep red blood cells (SRBC) was developed to facilitate the identification of adherent colonies growing in cultures of mixed tumour cells, established for the purpose of studying cellular interactions. Anti-tumour MoAbs were purified by affinity chromatography and adjusted to 1 mg ml$^{-1}$ in 0.9% w/v sodium chloride without buffers. A 1% w/v solution of chromic chloride was adjusted to pH 5.0 and left to 'age' for at least 3 weeks. SRBC were washed twice in 0.9% NaCl and spun down, and appropriate volumes of cell pellet were added to an equal volume of antibody, followed immediately by addition of $CrCl_3$ while agitated on a vortex mixer. The SRBC were left for 10 min at 20°C and washed twice in phosphate-buffered saline (pH 7.2) before resuspension in Eagle's MEM+10% calf serum. Mixed cultures containing tumour cell colonies were drained of medium and the MoAb-coated SRBC were added, followed by 20 min incubation at 20°C. The cultures were then rinsed free of unattached SRBC, fixed in methanol, and stained with Leishman's stain.

Using tumour cell lines and MoAbs of known reactivity, it was established that MoAb-coated SRBC adsorbed strongly to colonies of cells bearing the antigen recognised by the MoAb. SRBC treated with saline or a non-cross-reactive MoAb did not bind to cell colonies, although the sensitivity of the assay was such that weak cross-reactions gave a positive result. By careful matching of MoAbs to the cells used, this method provides a useful means of identifying colonies of cells with similar morphology in mixed cultures.

### Quantitation of endocytosis of antibody by a radiolabel method

M.C. Garnett, T.H.A.Y. Huehns, J.R. Martin, C.R.L. Graves & S.L. Smith

*Cancer Research Campaign Laboratories, University of Nottingham, Nottingham NG7 2RD, UK.*

The endocytosis of 791T/36 antibody has been previously demonstrated by the use of fluorescent conjugates (Garnett & Baldwin, *Eur. J. Cell Biology*, **41**, 214, 1986). However, use of such conjugates to quantitate the rate of uptake is difficult due to fluorescence quenching by various factors. Rate of uptake was therefore quantitated using $^{125}$I-labelled antibody.

After binding labelled antibody to cells at saturating levels, endocytosis was allowed to proceed for various times at 37°C. Bound non-endocytosed material was removed from cells by digestion with papain on ice, followed by centrifugation. The percent endocytosis compared to material originally bound at time zero was then calculated by the following equation:

Percent endocytosis $= P_t - S_t \times P_0/S_0 \times 100/P_0 + S_0$

where $P$ = cpm in pellet, $S$ = cpm in supernatant, subscript 0 = time 0 and subscript $t$ = time $t$.

This equation corrects for bound material not completely digested from the cell surface.

Use of this assay to quantitative uptake of $^{125}$I-791T/36 antibody on 791T cells showed 30% uptake after 4 h. The specificity of uptake was demonstrated by washing cells prior to endocytosis, but true rates of endocytosis were given by incubation with antibody throughout the assay. The latter method showed that uptake was linear for a minimum of 4 h, strongly indicative of a pinocytic rather than a receptor-mediated endocytic process.

### Radiosensitivity of peripheral blood lymphocytes in radiotherapy patients

J.O.T. Deeley, J. Marks, I.J. Kerby & J.L. Moore

*South Wales Cancer Research Council, Radiation Science Laboratory, Velindre Hospital, Whitchurch, Cardiff CF4 7XL, UK.*

When the nuclei of human lymphocytes are challenged with 2 M NaCl a histone-free-DNA-protein (HF-DNA) complex is released (Harris *et al.*, *Int. J. Radiat. Biol.*, **47**, 689, 1985; George *et al.*, *Br. J. Cancer*, **55**, 141, 1987). The sedimentation distance of the HF-DNA complex in a linear sucrose gradient (pH 8.0) is less when immediately isolated from *in vitro* irradiated cells than that when isolated from unirradiated cells. Typically, there is a non-linear dose response in the sedimentation behaviour. At low doses, if irradiated cells are incubated at 37°C the sedimentation behaviour approaches that of unirradiated cells ('repair') – generally, repair is complete after an incubation of 1 h at 37°C.

The repair of irradiated lymphocytes derived from 'healthy' control donors was similar to that of lymphocytes derived from patients with carcinoma of the uterine cervix (blood was taken when the patient presented at the first clinic for radiotherapy). Most donors demonstrated complete repair following an incubation of 1 h at 37°C. However, 2/28 (7%) controls and 2/25 (8%) patients were repair deficient showing a less than 30% repair following incubation. In addition, the repair of irradiated lymphocytes derived from patients treated by radiotherapy 2 to 5 years previously for carcinoma of the uterine cervix has been examined. Of those now attending with 'bowel complications' attributed to radiotherapy 7/16 (44%) showed a less than 30% repair. In contrast, all those (11/11) described as 'well and complication-free' showed good repair, that is greater than 80% repair on incubation.

**The activation of CB1954 (2,4-dinitro-5-aziridinyl benzamide) in Walker cells is by bioreduction to a DNA crosslinking agent**

R.J. Knox, F. Friedlos & J.J. Roberts

*Institute of Cancer Research, Sutton, Surrey, SM2 5PX, UK.*

Walker tumour cells were shown to be highly sensitive to the cytotoxic effects of the compound CB 1954, a monofunctional alkylating agent, when compared with other cells *in vitro*. However CB 1954 forms DNA–DNA inter-strand crosslinks (as determined by alkaline sucrose gradient sedimentation analysis) in a time-dependent manner in Walker tumour cells but not in V79 cells, which are not sensitive to this agent. The absence of inter-strand crosslinks in hamster cells was not due to a lack of uptake of the drug but rather to their failure to convert (probably by bioreduction) CB 1954 to a difunctional intermediate, the mechanism for which has now been identified in Walker cells. As Walker cells are at least 10,000 fold more sensitive, on a dose basis, than are hamster cells it is of considerable interest to determine if some human tumour cells are also capable of activating, or can be made to activate CB 1954 to a difunctional agent.

**Independent action of radiation and platinum compounds on human foetal lung cells *in vitro* and human melanoma cells *in vivo***

C. Basham[1], J. Mills[1], E.B. Douple[2] & J.J. Roberts[1]

[1]*Institute of Cancer Research, Sutton, Surrey, SM2 5PX, UK;* [2]*Dartmouth–Hitchcock Medical Center, Hanover, NH, USA.*

Confluent (stationary phase) human foetal lung cells recover from damage induced by either radiation or *cis*-platin but with different kinetics, that from radiation being maximal after 2 days, irrespective of the initial dose, while that from *cis*-platin was continuous for up to 10 days. At late times after treatment of cells with a combination of the two agents the cells exhibited a toxic response equal to the sum of that produced by either agent alone. Irradiation of exponentially growing human foetal lung cells before, during or following a 1 h pulse treatment with *cis*-platin also resulted in a toxic effect equal to the sum of that produced by either agent alone. CBA nude mice implanted with a human melanoma were either irradiated (12.5 Gy), injected with carboplatin ($100\,mg\,kg^{-1}$) or injected with carboplatin 45 min before irradiation. The effect of these treatments on the survival of tumour cells was determined by their colony-forming ability *in vitro* 1 h later. The combination of carboplatin and radiation produced an effect on cell survival equal to the sum of the two separate treatments. Thus radiation and platinum compounds act independently on both normal and tumour human cells, a finding which suggests a common mechanism of action and accounts for the beneficial therapeutic effects obtained when they are used in combination.

**Thymidine kinase activities in pleural effusions**

G.B. Nevin[1], K.L. O'Neill[2] & P.G. McKenna[2]

[1]*Biomedical Sciences Research Centre, University of Ulster, Newtownabbey BT37 0QB;* [2]*Biomedical Sciences Research Centre, University of Ulster, Coleraine BT52 1SA, UK.*

The levels of the nucleotide salvage pathway enzyme thymidine kinase (TK), were assayed in the pleural effusions of 28 patients undergoing cytological examination for lung cancer. Of the 28 patients, 12 had a positive cytological diagnosis of malignancy. The effusions from these affected patients were found to have a mean total TK level of $4681.5 \pm 725.0\,cmp\,ml^{-1}\,min^{-1}$. Patients in whom malignancy was not diagnosed had a mean total TK activity of $4371.1 \pm 787.4\,cpm\,ml^{-1}\,min^{-1}$. TK exists in two forms, TK1 and TK2, which can be distinguished on the basis of phosphate donor specificity. Both TK1 and TK2 can utilize ATP as phosphate donor. TK1 can utilize CTP as phosphate donor with approximately 15% the efficiency found with ATP. TK2 can utilize CTP as phosphate donor with approximately 80% the efficiency found with ATP. The patients with a positive diagnosis of malignancy were found to have a mean TK activity, using CTP as phosphate donor, of $1480.7 \pm 307.4\,cpm\,ml^{-1}\,min^{-1}$. Patients with a negative cytological diagnosis had a mean (CTP) TK activity of $2781.3 \pm 391.1\,cpm\,ml^{-1}\,min^{-1}$. The two groups were significantly different in this regard ($P<0.05$). When the two groups were compared for percent CTP/ATP TK activity the patients with a positive cytological diagnosis for malignancy had a mean value of $35.97 \pm 5.95\%$, while patients with a negative cytological diagnosis for malignancy had a mean percent CTP/ATP TK activity of $66.15 \pm 3.6\%$. The two groups were significantly different in this regard ($P<0.001$). These results indicate that pleural effusions from patients with lung cancer may have increased TK1 levels and decreased TK2 levels relative to patients without malignancy. Measurement of TK levels may be a useful adjunct to cytological diagnosis for malignancy in pleural effusions.

**The combined use of the tumour antigens CEA, CA19–9 and CA50 in discrimination of oesophagogastric carcinomas**

M. Lucarotti, S. Kelly, N.A. Habib, D.J. Leaper, M. Hershman, R.C.N. Williamson & C. Wood

*University Departments of Surgery, Bristol Royal Infirmary, Bristol and Royal Postgraduate Medical School, Hammersmith Hospital, London, UK.*

The tumour-associated antigens CEA, CA19-9 and CA50 defined by monoclonal antibodies have been raised against human colorectal adenocarcinoma cell lines. The aim of this study was to assess their combined use to identify patients with oesophagogastric carcinoma.

An immunoradiometric assay was used for the detection of CEA and CA19-9 and the Delfia system was used for CA50. Serum was collected from 50 control patients (of whom 23 had benign upper gastrointestinal disease and 27 were normal subjects) and 23 patients with oesophagogastric carcinoma. The 'cut-off' discriminant levels for CEA, CA19-9 and CA50 were $5.4\,ng\,ml^{-1}$, $33.2\,U\,ml^{-1}$ and $35.5\,U\,ml^{-1}$ respectively.

The combination of antibodies was positive in only 1 (2%) of the control group and 16 of 23 (70%) with oesophagogastric carcinoma (Table).

| *Antibody* | *% Positive in patients with oesophagogastric carcinomas* |
|---|---|
| CEA | 35 |
| CA19-9 | 52 |
| CA50 | 35 |
| Combined | 70 |

A combination of all three tumour markers may help to identify oesophagogastric malignancy and could be useful in monitoring progression and treatment.

### The clinical use of CA19-9 in the differential diagnosis of benign and malignant disease of the gastrointestinal tract

M. Lucarotti, S. Kelly, N.A. Habib, D.J. Leaper, M. Hershman, R.C.N. Williamson & C. Wood

*University Departments of Surgery, Bristol Royal Infirmary, Bristol; and Royal Postgraduate Medical School, Hammersmith Hospital, London, UK.*

CA19-9 is a tumour-associated carbohydrate antigen from a human colorectal adenocarcinoma cell line. This study investigated the role of CA19-9 in the separation of benign from malignant gastrointestinal disease.

Serum was collected from 50 control patients, 53 with benign gastrointestinal disease and 136 patients with carcinoma of the gastrointestinal tract. An immunoradiometric assay (CIS UK) was used to detect CA19-9 in the serum and a level of $33\,U\,ml^{-1}$ was used as a 'cut-off' to distinguish between benign and malignant disease.

Serum levels in 10 of the 53 patients (19%) with benign disease, 20 of 33 (61%) with upper gastrointestinal carcinoma and 28 of 103 (27%) with colonic carcinoma had levels above $33\,U\,ml^{-1}$ (Table).

| *Carcinoma type* | *No. patients* | *% +ve CA19-9* |
|---|---|---|
| Pancreatic | 10 | 90 |
| Oesophagogastric | 23 | 52 |
| Colonic | 103 | 25 |

Therefore CA19-19 may be of help in distinguishing benign from malignant disease of the upper gastrointestinal tract but does not help recognise colonic carcinoma.

### Predicting response to combined pre-operative radiotherapy and surgery in rectal cancer

D.J. Jones[1,3], J. Zaloudik[3], R.D. James[2], M. Moore[3] & P.F. Schofield[1]

*Departments of [1]Surgery and [2]Radiotherapy; and [3]Paterson Institute for Cancer Research, Christie Hospital, Manchester M20 9BX, UK.*

Local recurrence after resection of rectal carcinomas is reduced by pre-operative radiotherapy and factors which predict response to radiotherapy could help with patient selection. Flow cytometric DNA ploidy status is related to prognosis in colorectal cancer (Jones *et al.*, *Br. J. Surg.*, **75**, 28, 1988) and has been evaluated in relationship to response to radiotherapy in 185 patients with rectal cancer. Ninety-six were randomised to surgery alone, and 89 to pre-operative radiotherapy (2,000 cGy, over 4 days) and surgery.

DNA aneuploidy was detected in the resected specimens of 59 (61%) patients treated by surgery alone, but in only 33 (37%) after radiotherapy ($X^2=11.23$, $P<0.001$). DNA aneuploidy was detected in 30/44 (68%) pre-radiotherapy biopsies but in only 15/44 (34%) of these tumours after radiotherapy.

Preliminary follow-up data show a possible recurrence free survival advantage for patients in the radiotherapy group ($P=0.07$). Patients with DNA diploid tumours had a recurrence free survival advantage ($P=0.052$), but this was confined to the surgery only group. In the radiotherapy group recurrence free survival rates for diploid and aneuploid tumours were similar and comparable to those for diploid tumours in the surgery only group. These preliminary data suggest the improved recurrence free survival after radiotherapy is mainly confined to patients with DNA aneuploid tumours.

### Patterns of epithelial cell proliferation of colorectal mucosa in normal subjects and in patients with large bowel tumours

M. Ponz de Leon, L. Roncucci, C. Sacchetti, P. Di Donato & L. Tassi

*Istituto di Patologia Medica, Policlinico di Modena, 41100 Modena, Italy.*

In order to define the predictive and discriminatory value of autoradiography we evaluated epithelial cell proliferation in different large bowel segments of subjects with a negative pancolonscopy and in rectal mucosa of patients with adenomatous polyps or cancer. Samples of colorectal mucosa were taken during endoscopy at $\sim 10$ cm from the anal verge. In controls, samples were also taken in caecum, ascending, transverse, descending and sigmoid colon. After standard autoradiography and histological preparation, each intestinal hemicrypt was divided into 5 longitudinal compartments from the base (comp. 1) to the surface (comp. 5) and labelled cells (S phase of the replicative cycle) in each compartment were counted. In controls, total Labelling Index (LI = ratio of labelled to total cells) and LI per crypt compartment (ratio of labelled to total cells in each compartment) did not show any significant difference among the various large bowel tracts. Total LI was higher in patients with colorectal neoplasms than in controls (but not significantly). In contrast, LI per crypt compartment in the most superficial parts of the crypt (comp. 3–5) was significantly ($P<0.01$ at least) higher in the two groups of patients than in controls. In the fifth compartment labelled cells were seen in only 15.8% of controls but in 71% and 87.5% of polyps and cancer patients. *In conclusion*, in controls cell proliferation was similar in the various large bowel segments, thus the rectum seems representative of the entire large bowel. In patients with colorectal tumours an upward expansion of the proliferative zone was observed; labelling of compartment 5 showed the highest discriminatory power between subjects with or without intestinal neoplasms.

### Increased thrombin activity in patients with small cell lung cancer

R. Milroy[1], J.T. Douglas[2], J. Campbell[1], R. Carter[1], G.D.O. Lowe[2] & S.W. Banham[1]

*[1]Department of Respiratory Medicine, and [2]University Department of Medicine, Royal Infirmary, Glasgow, UK.*

Abnormalities of haemostasis have been extensively reported in cancer patients. However, their relationship to response to treatment and prognosis are not known. We report a study of 37 patients with small cell lung cancer (SCLC). Estimations of thrombin activity and plasmin-mediated fibrinolysis were obtained by measurement of plasma concentrations of the fibrinopeptides A (FpA) and B$\beta$ 15–42 antigen (B$\beta$) respectively. There was evidence for increased thrombin activity (median FpA was $13.2\,mol\,ml^{-1}$ [normal $<4\,pmol\,ml^{-1}$]) but only modestly increased fibrinolysis (median B$\beta$ was $5.4\,pmol\,ml^{-1}$ [normal $<3\,pmol\,ml^{-1}$]). Furthermore, the ratio of thrombin generation to fibrinolysis (FpA/B$\beta$ ratio) was raised at 2.2 (normal $<1.3$). Twenty-six of these patients completed induction chemotherapy and were evaluable for response: 14 complete responders, 7 partial responders and 5 non-responders. There was a significant association between increased thrombin activity (FpA levels) and lack of response to chemotherapy ($P<0.01$, Mann–Whitney U-test). In addition non-responders had evidence of an increased ratio of thrombin generation to fibrinolysis ($P<0.05$, Mann–Whitney U-test). We studied the same haemostatic parameters in 9 patients who have been in complete remission for at least 2 years after chemotherapy for SCLC. In this group median FpA was normal, at 3.3 pmol ml$^{-1}$, and although median B$\beta$ was modestly elevated (7.1 pmol ml$^{-1}$), the median FpA/B$\beta$ ratio was normal (0.41). The difference in thrombin activity and also in the ratio of thrombin generation to fibrinolysis between these two groups was significant ($P<0.001$, Mann–Whitney U-test). This is the first study in cancer patients to show a relationship between increased thrombin activity and a lack of response to chemotherapy.

### Structural changes in cytokeratin intermediate filaments during *in vivo* epithelial preneoplasia

F.H. White & K. Gohari

*Department of Anatomy, University of Hong Kong, Hong Kong.*

Cytokeratin filaments are intermediate filaments found in epithelial tissues which become concentrated in the superficial squames of keratinised epithelia. During malignant transformation in such epithelia, abnormal intraepithelial keratinisation, or dyskeratosis, is common. In this report, we establish the quantitative characteristics of cytokeratin aggregates in hamster cheek pouch epithelium treated with the carcinogen DMBA. Such treatment produces hyperplastic and dysplastic lesions prior to overt neoplasia.

Tissue samples from 5 animals each with dysplastic and hyperplastic lesions were processed for electron microscopy. Five untreated animals were used as controls. Volume density measurements of cytokeratin filament aggregates were determined by stereological procedures in defined basal, spinous and granular compartments of the epithelium. Determination of cellular volumes for each compartment enabled the density data to be transformed to average cell values.

Volume densities of filaments were similar in carcinogen-treated epithelia when compared with normal epithelia, but average cell volumes of filaments in each compartment increased progressively following carcinogen treatment. We conclude that carcinogen-induced preneoplastic lesions are accompanied by increased synthesis of cytokeratins, which may be related to the premature abnormal keratinisation so frequently seen in dysplastic epithelium.